# Current aviation roadmaps are not within planetary boundaries

**DOI:** 10.1038/s41467-026-77472-6

**Published:** 2026-10-13

**Authors:** Bastien Païs, Alexandre Gondran, Lorie Hamelin, Florian Simatos

**Affiliations:** 1 Fédération ENAC ISAE-SUPAERO ONERA, Université de Toulouse, Toulouse, France; 2 Agence de l’environnement et de la Maîtrise de l’Energie, Angers, France; 3 ENAC, French Civil Aviation University, Université de Toulouse, Toulouse, France; 4 TBI, Université de Toulouse, CNRS, INRAE, INSA, Toulouse, France

**Keywords:** Environmental impact, Environmental sciences

## Abstract

Aviation decarbonization roadmaps predominantly rely on replacing fossil kerosene with alternative jet fuels, yet the systemic impact of these alternatives on planetary boundaries other than climate change has not been quantified. Here we integrate prospective life-cycle inventories with the planetary boundaries framework to evaluate the absolute impacts of aviation roadmaps as time-dependent trajectories, rather than endpoint snapshots. Across scenarios to 2050, we find that fuel-switch-centered roadmaps do not achieve sustainability, persistently transgress the climate change and biosphere integrity core boundaries, and exert large demands for energy, land, freshwater and CO_2_ capture that challenge their feasibility. Slower traffic growth brings aviation closer to its share of the safe operating space in terms of lowering energy demand, but respecting additional planetary boundaries requires air traffic to decline by at least 7.2% yr^−1^. Our results show that aligning aviation with Earth’s safe operating space cannot be achieved through fuel substitution alone and requires governance strategies that combine supply-side solutions with explicit demand-side measures.

## Abstract (web summary)

This study broadens the impact assessment of current aviation roadmaps to the planetary boundaries framework. It finds that none is absolutely sustainable, triggering the need to also consider demand-side solutions for a future sustainable aviation.

## Introduction

Human activity has pushed the Earth system beyond seven of the nine planetary boundaries (PBs), eroding its resilience and highlighting the urgent need to operate within a safe environmental space^1–5^. While climate change mitigation has been central to global policy and scientific discourse, recent analyses reveal a critical gap: climate-focused development pathways often fail to safeguard other PBs^4^.

Aviation exemplifies this systemic challenge. The sector is essential for global connectivity and economic development, yet it exerts substantial climate impacts^6^, and its broader societal value is debated^7–9^. Moreover, emissions from international aviation are not covered by the Paris Agreement^10^, requiring distinct governance mechanisms. In response, institutional and industrial stakeholders have produced aviation-specific modeling scenarios to identify ways forward toward more sustainable futures for the sector, based on assumptions covering future demand, aircraft efficiency, and the carbon intensity of energy sources^11–13^. Transitioning from fossil kerosene to alternative energy carriers with lower fossil carbon content is consistently identified as the most promising technological lever, with some studies attributing up to 70% of their modeled CO₂ emission reductions to this shift^11^. However, these scenarios often overlook critical trade-offs. Life-cycle assessments (LCAs) indicate that replacing fossil kerosene with biofuels or efuels increases impacts related to nutrient availability, water and land use due to pressures on energy, land, and freshwater resources^14–16^. Despite this, the impacts on PBs other than climate change are not considered in recent analyses of aviation transition scenarios^14,15,17,18^, and many studies do not account for the full life cycle of fuel production, leading to unwarranted assumptions such as “zero-carbon” fuels^17,19^.

Here, we address these gaps by developing an absolute environmental sustainability assessment (AESA) of currently proposed aviation roadmaps, which comprise a wealth of alternative technologies combined in a set of scenarios. AESA defines sector-specific budgets for each PB and evaluates scenario impacts against these thresholds to determine absolute sustainability^20^. To perform this AESA, we build on the methodology developed by Ryberg et al.^21^ and Bjørn et al.^20^, which combines Life Cycle Impact Assessment (LCIA) with the PB framework (PB-LCIA). We adapt the PB-LCIA methodology to add new dimensions, including (i) temporality, thereby allowing us to consider the entire 2023–2050 timespan for prospective assessments, (ii) the assessment of biosphere integrity, a core PB, and (iii) allocation principles rooted in ethical considerations for determining the aviation sector’s share of the Earth’s safe operating space for each PB^22^. Our findings demonstrate that none of the currently proposed roadmaps for climate mitigation bring the aviation sector to within the safe operating space of the Earth’s planetary boundaries, with systematic transgression of both climate change and biosphere integrity, the so-called core PBs^2^. We further show that the environmental burdens induced by efuels and biofuels are highly sensitive to production pathways. Our results suggest that technological improvements alone are insufficient for aviation to remain within planetary boundaries, and that combined actions including ambitious demand-side measures are required to navigate back toward a safe operating space.

## Results

### Scenario construction and absolute environmental sustainability assessment

Our work assesses the absolute environmental sustainability of influential roadmaps for commercial aviation proposed recently by institutional and industrial stakeholders, namely: the Long-Term Aspirational Goals (LTAG) scenarios proposed by the International Civil Aviation Organization (ICAO)^12^, the Waypoint 2050 scenarios proposed by the Air Transport Action Group (ATAG)^11^ and the ReFuelEU Aviation initiative adopted by the European Union (EU)^23^. The main lever considered by all these scenarios for climate mitigation is to offset, whether partly or fully, fossil kerosene through mobilizing a variety of alternative energy carriers (AEC) (Table 1). ICAO’s LTAG scenarios^12^ are labeled F1 to F3: F1 emphasizes low-cost greenhouse gas reduction, F2 prioritizes effective greenhouse gas reduction, and F3 maximizes CO_2_ reduction. The ATAG Waypoint scenarios^11^ are labeled S1 to S3: S1 intensifies technology and operations, S2 considers ambitious sustainable fuel deployment, and S3 has an aspirational and ambitious technology perspective.

**Table 1:** Main characteristics of the institutional and industrial roadmaps modeled in this work

Stakeholder	Aviation roadmaps	Traffic growth rate (% yr^−^^1^)	Efficiency gains (% yr^−^^1^)	Categorization of AECs, as labeled in roadmaps (contribution of corresponding AEC to the 2050 aviation fuel demand)	Contribution of fossil kerosene and LCAF to the 2050 aviation fuel mix
ICAO	Mid LTAG scenarios F1-F3^12,25^	2.8–4.2	0.7–1.3	• Efuel from DAC (0–10%) • Efuel from waste CO_2_ (8–45%) • Biofuel (19–53%) • LCAF (0–28%) • Liquefied hydrogen (0–4%)	0–73%
ATAG	Waypoint scenarios S1-S3^11^	2.3–3.3	1.4–1.9	• Efuel (37–51%) • HEFA (2–8%) • Advanced biofuel (FT+AtJ) (32–46%)	11%
European Commission	ReFuelEU mandates^23^	-	-	• Efuel (35%) • Biofuel (35%)	30%

Traffic is measured in passengers.km (pkm) and efficiency in MJ pkm^−^^1^. The alternative energy carriers (AECs) considered are LCAF (Lower-Carbon Aviation Fuel, produced from fossil carbon and hydrogen from water electrolysis); efuel; HEFA (Hydroprocessed Esters and Fatty Acids); FT (Fischer–Tropsch); AtJ (Alcohol-to-Jet); LH₂ (liquefied hydrogen). DAC refers to Direct Air Capture. The roadmaps considered are those of ICAO (International Civil Aviation Organization, Long-Term Aspirational Goal (LTAG) scenarios), ATAG (Air Transport Action Group, Waypoint 2050 scenarios), and the European Commission ReFuelEU mandates.

**Fig. 1:**
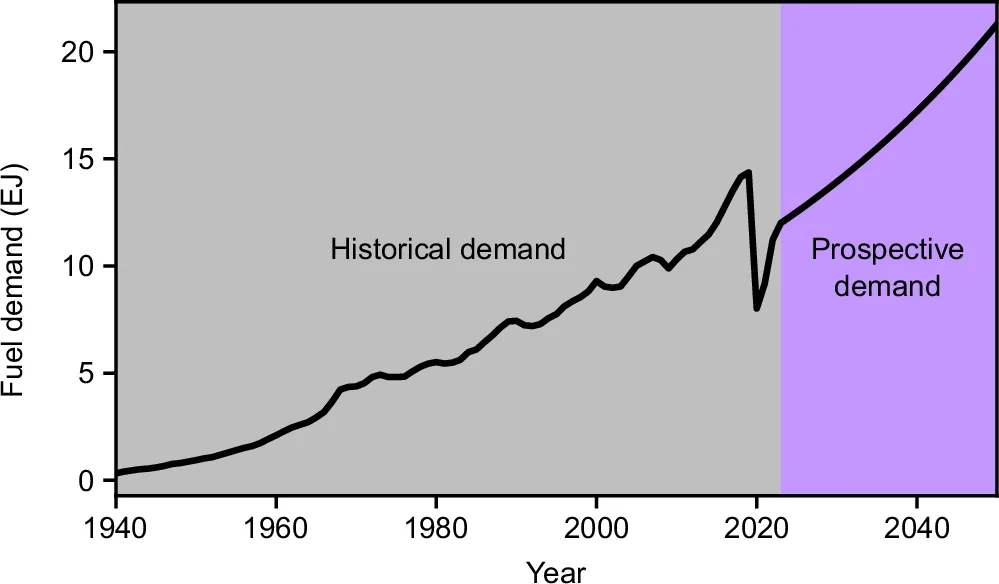
Global aviation fuel demand. Global aviation fuel demand (in exajoule [EJ]), common to the nine scenarios considered, distinguishing between the historical part (1940–2022) and the prospective part (2023–2050). The prospective fuel demand results from assumptions on traffic growth (3.7% yr^−^^1^) and efficiency improvements (1.5% yr^−^^1^). Historical energy demand is retrieved from Lee et al. ^48^ and the International Air Transport Association (IATA)^52^ (see “Methods” section).

These roadmaps are herein represented as contrasted scenarios. Aviation fuel demand (2023–2050) is set to be the same for all scenarios (Fig. 1) and is determined by considering an initial consumption of 12 EJ in 2023^24^, an increase in traffic of 3.7% yr^−^^1^ and improvements in efficiency due to aircraft technology and operations of 1.5% yr^−^^1^. The traffic growth rate corresponds to the central value of the ICAO scenarios^25^, while the efficiency improvements rate lies within the range reported in the Waypoint 2050 scenarios^11^. Together, these assumptions are consistent with industrial and academic forecasts^6,26^. They result in a global fuel demand that increases at a rate of 2.1% yr^−^^1^ between 2023 and 2050. Moreover, to ensure methodological consistency with the PB framework, the historical period 1940–2022, accounting for past impacts of aviation, is also considered (see Fig. 1 and “Methods” section). The values for the traffic growth rate and efficiency gains are varied in the ICAO and ATAG range in the Supplementary Figs. 9 and 16, which also assess the robustness of our results to the uncertainty of historical emissions (Supplementary Fig. 10).

In order to model the ICAO, ATAG and EU scenarios, the categorization of AECs, which differs depending on the stakeholder considered (Table 1), is homogenized by considering five AECs: efuel, three biofuels (Alcohol-to-Jet [AtJ], Fischer–Tropsch [FT] and Hydroprocessed Esters and Fatty Acids [HEFA]), and liquefied hydrogen (LH_2_). The specific production pathway (e.g., which feedstock is used for biofuel or which electrolysis technology for hydrogen production) of a given AEC has a strong influence on its environmental performance^27^. However, the ICAO, ATAG and EU roadmaps do not enter this level of detail, and so further assumptions are necessary. Given that these roadmaps were proposed to mitigate climate change, we assumed, for each AEC, the most favorable production pathway for climate (Table 2 and “Methods” section). To assess the sensitivity of our results to this choice of production pathways, a different choice of biofuel production pathways geared toward mitigating impacts in a balanced way across PBs is also considered in the Supplementary Table 5 and Supplementary Figs. 8, 14 and 15.

**Table 2:** Description of the production pathway (combination of feedstock and conversion pathway) selected based on climate performance for each alternative energy carrier

AEC	Feedstock	Conversion pathway	Source of LCI
Electricity-based AECs			
Efuel	• Grid electricity (except Efuel-RES) • 100% Renewable electricity sources (only scenario Efuel-RES) • Water	• H_2_ production by PEM electrolysis • Atmospheric CO_2_ captured by direct air capture with a solvent-based system • Fischer–Tropsch conversion pathway	Van der Giesen et al.^61^
LH_2_	• Grid electricity (all scenarios except Efuel-RES) • Renewable electricity sources (only scenario Efuel-RES) • Water	• PEM electrolysis	Thonemann et al.^62^
Biomass-based AECs			
AtJ	• Sawdust	• Alcohol-to-Jet	Björnsson and Ericsson^63^
FT	• Pinewood	• Fischer–Tropsch with carbon capture and storage	Almena et al.^64^
HEFA	• Pennycress	• Hydroprocessed Esters and Fatty Acids	Mousavi-Avval and Shah^65^

For each alternative energy carrier (AEC), the table indicates the feedstock, the conversion pathway, and the source of the Life Cycle Inventory (LCI) used in this study.

*PEM* proton exchange membrane, *FT* Fischer–Tropsch, *HEFA* hydroprocessed esters and fatty acids, *AtJ* alcohol-to-jet, *LH₂* liquefied hydrogen, *RES* renewable energy sources, *CCS* carbon capture and storage.

**Fig. 2:**
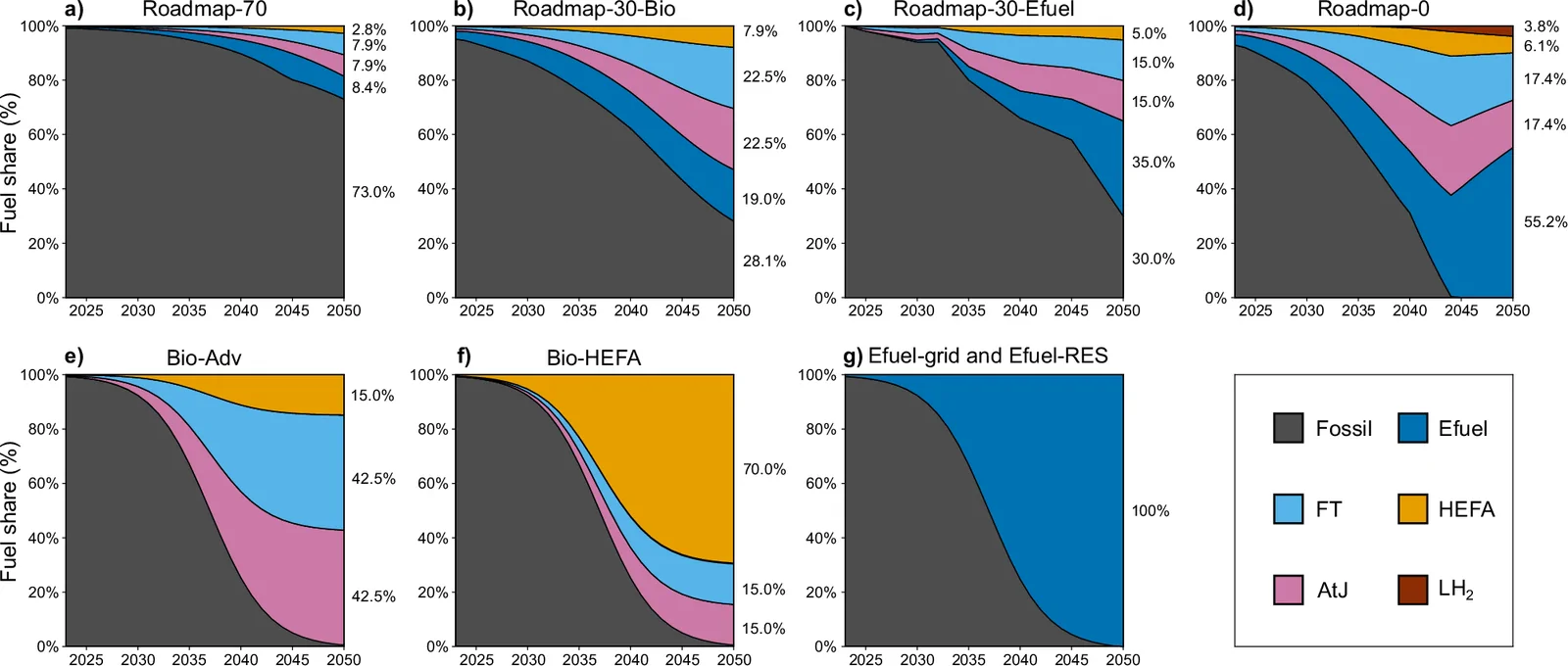
Evolution of the fuel mix in the eight scenarios considered. Each panel shows the evolution between 2023 and 2050 of the relative share of fossil kerosene and of the five alternative energy carriers (AECs) considered (efuel, FT [Fischer–Tropsch], HEFA [Hydroprocessed Esters and Fatty Acids], AtJ [Alcohol-to-Jet], LH_2_ [liquefied hydrogen]) used to fulfill the global aviation fuel demand. **a** Roadmap-70. **b** Roadmap-30-Bio. **c** Roadmap-30-Efuel. **d** Roadmap-0. **e** Bio-Adv. **f** Bio-HEFA. **g** Efuel-grid and Efuel-RES. Efuel-grid and Efuel-RES differ in the source of electricity considered: for Efuel-grid (as well as all other scenarios except Efuel-RES), electricity comes from the grid, while for Efuel-RES it comes from dedicated renewable energy sources (RES). Values on the right-hand axis indicate the proportion of the corresponding AEC in the 2050 fuel demand. Reading example: in 2050, the aviation fuel demand in Roadmap-30-Bio is addressed by a mix of HEFA (7.9%), FT (22.5%), AtJ (22.5%), Efuel (19%) and fossil kerosene (28.1%).

To model the current institutional and industrial roadmaps, we consider four scenarios ranging from Roadmap-70, where fossil kerosene fulfills 73% of the 2050 total energy demand, to Roadmap-0 with a complete phasing out of fossil kerosene in 2045 and an ambitious deployment of electricity-based aviation energy carriers (efuel and LH_2_) supplying nearly 60% of the 2050 energy demand (Fig. 2 and Table 2). LH_2_ is only present in Roadmap-0, where we make the assumption that the cryogenic aircraft has the same efficiency performance as the corresponding conventional aircraft. It is important to note that this is an optimistic assumption, as current forecasts for the gravimetric index of liquid hydrogen tanks would lead to cryogenic aircraft being less efficient than current conventional aircraft^28^. Roadmap-30-Bio and Roadmap-30-Efuel are intermediate scenarios, where fossil kerosene meets around 30% of the 2050 demand. They differ in that Roadmap-30-Bio relies more on biofuels than Roadmap-30-Efuel. Roadmap-70, Roadmap-30-Bio and Roadmap-0 closely follow the three Mid LTAG scenarios F1, F2, and F3 of ICAO^12^, respectively, while Roadmap-30-Efuel considers the evolution of the energy mix of the ReFuelEU Aviation initiative. The Waypoint 2050 scenarios S1, S2, and S3^11^ assume 10% of residual fossil kerosene in 2050 and thus lie between Roadmap-30 and Roadmap-0, and their shares of biofuels and efuels also vary by the ranges covered by the Roadmap scenarios (Supplementary Fig. 1).

In light of recent academic results^14,19^ and institutional roadmaps^29^, we also consider four “mono-type” scenarios where the 2050 demand is met either entirely with efuels or entirely with biofuels (Fig. 2 and Table 3). Although fictitious, these scenarios supply valuable insights into the influence of the primary energy and production pathway used. Two biofuel scenarios consider the case where the 2050 demand is met with biofuels, either with a majority of HEFA biofuels (scenario Bio-HEFA) or with a majority of advanced (FT and AtJ) biofuels (scenario Bio-Adv). In the two efuel scenarios, the 2050 demand is met with efuels where electricity either comes from the grid (scenario Efuel-grid) or from dedicated renewable energy sources (scenario Efuel-RES, see “Methods” section).

**Table 3:** Definition of the nine scenarios analyzed and concordance with existing institutional or industrial roadmaps

Scenario in the present study	Institutional or industrial roadmap on which the scenario is based	Comments
Institutional and industrial roadmaps		
Roadmap-70	LTAG-F1-Medium	Comparison with ICAO scenarios: • Lower-carbon aviation fuel considered as fossil kerosene • “Biofuel” category divided into AtJ, FT, HEFA using Waypoint repartition for HEFA vs FT+AtJ, and then splitting equally between FT and AtJ Comparison with ATAG scenarios: • The Waypoint scenarios are in-between the LTAG-F2-Medium and LTAG-F3-Medium scenarios • “Advanced biofuel” category divided equally into FT and AtJ
Roadmap-30-Bio	LTAG-F2-Medium	
Roadmap-0	LTAG-F3-Medium	
Roadmap-30-Efuel	ReFuelEU	“Biofuel” category divided into AtJ, FT, HEFA using Waypoint repartition for HEFA vs FT+AtJ, and then splitting equally between FT and AtJ
Mono-type scenarios		
Bio-HEFA	N/A	100% biofuel in 2050 (70% HEFA, 15% FT, 15% AtJ)
Bio-Adv	N/A	100% biofuel in 2050 (15% HEFA, 42.5% FT, 42.5% AtJ)
Efuel-Grid	N/A	100% efuel from grid electricity in 2050 (electricity mix determined by underlying development storyline)
Efuel-RES	N/A	100% efuel from renewable energy sources in 2050 (33% PV, 33% wind onshore, 33% wind offshore)
Baseline	N/A	100% fossil kerosene

The first four scenarios (Roadmap-70, Roadmap-30-Bio, Roadmap-0 and Roadmap-30-Efuel) are derived from institutional and industrial roadmaps (ICAO LTAG, ATAG Waypoint 2050, and the European Commission ReFuelEU mandates). The five subsequent scenarios (Bio-HEFA, Bio-Adv, Efuel-Grid, Efuel-RES and Baseline) are mono-type scenarios in which the 2050 aviation fuel mix is assumed to consist entirely of one type of alternative energy carrier (or fossil kerosene for the Baseline).

*PV* photovoltaic. Acronyms of alternative energy carriers are defined in Table 2.

Finally, these scenarios are compared to a “baseline” scenario where all future fuel demand is met with fossil kerosene.

In the prospective range 2023–2050, PB results depend on future technological evolutions, e.g., electricity production methods or the efficiency of production pathways. To account for this, we mobilized the premise (v2.2.0) package^30^, supplying an underlying development storyline provided by Integrated Assessment Models (IAM) that is used to project life-cycle inventories through time (see “Methods” section). Results are reported for a development storyline broadly consistent with the Paris Agreement goals (i.e., SSP2-RCP2.6) while results for three other storylines spanning a wide range of possible futures are considered in the SI (see “Methods” section and Supplementary Figs. 11–13).

The first step in assessing the absolute environmental sustainability of aviation is to compute, for each PB, an allocated share of its safe operating space to the aviation sector. These allocated shares then serve as environmental budgets to which impacts are compared. Eventually, this allows us to determine whether a scenario is sustainable in absolute terms or not, i.e., whether it respects or transgresses its allocated shares.

The allocation necessarily includes ethical value judgments, such that a diversity of principles needs to be considered in order to reflect different possible ethical perspectives^20,31–33^. Here, we consider two allocation principles based on economic and ethical considerations. The economic-based allocation assigns to each PB a share equal to the current contribution of the aviation sector to the global gross domestic product. On the other end of the spectrum, we implement the ‘fulfillment of human needs’ principle^22^, a recently proposed allocation principle based on ethical considerations that computes shares based on the current situation of selected reference countries deemed sustainable from an environmental and social perspective (see “Methods” section and Supplementary Tables 7–9).

Prospective life-cycle inventories are used to translate each scenario’s impacts at the PB level, mobilizing the characterization factors proposed by Ryberg et al.^21^. We consider impacts on six PBs (counting the nitrogen and phosphorus cycles as two PBs): climate change, biosphere integrity, nitrogen and phosphorus cycles, freshwater use and stratospheric ozone depletion (see “Methods” section).

The PB framework does not convey information on the feasibility of the roadmaps in terms of resource availability. For instance, the freshwater use PB only accounts for the net amount of water withdrawn from freshwater sources, so that aviation could be considered sustainable in absolute terms for this PB even if it required all available freshwater on Earth, provided the water used is returned to freshwater sources. To help evaluate the feasibility of these roadmaps, we complement the AESA with an assessment of resource requirements in terms of energy (electricity and bioenergy), land occupation, freshwater withdrawal, and carbon sequestration by direct air capture (DAC), which is required to produce efuels. The absolute amount of these resources required in 2050 is computed for each scenario, and is compared to the global availability of resources in the underlying development storyline in order to provide insight into the significance of these absolute amounts (see “Methods” section and Supplementary Figs. 12–16).

### Ethics-based aviation PB budgets vary substantially and are smaller than those based on economic considerations

Results for the computation of aviation PB budgets under the economic and the ethics-based allocation principles are reported in Table 4. By construction, economic-based shares are the same for all PBs, and are computed to be equal to 2.9%, which corresponds to the direct, indirect, and induced contribution of aviation to the global gross domestic product in 2018^34^. In contrast, the ethics-based allocation leads to shares ranging from 0.002% (phosphorus cycle) to no higher than 2.2% (freshwater use). The ethics-based allocation is based on an application of the conceptual approach developed by Heide et al.^22^ that aims to reflect the importance of the necessities delivered to users. The very small shares observed under the ethics-based allocation for biosphere integrity, nitrogen and phosphorus cycles reflect that aviation currently relies on fossil kerosene with very low impacts on these PBs. This can be seen as a limitation of the method already pointed out in its original conceptual description, which tends to perpetuate a status quo situation potentially adverse to new technologies appearing in the future. Despite this shortcoming, the fulfillment of human needs principle is, to our knowledge, currently the only actionable allocation principle based on ethical considerations.

**Table 4:** Safe operating space and share allocated to commercial aviation for each planetary boundary

Planetary boundary	Control variable^2^	SOS for humanity^2^	Economic-based allocation	Ethics-based allocation
Climate change	Energy imbalance at the top of the atmosphere	1 W m^−^^2^	2.90%	0.9%
Change in biosphere integrity	Biodiversity Intactness Index	10% BII loss		0.012%
Biogeochemical flows - Nitrogen	Biological fixation of N, global	62 Tg N yr^−^^1^		0.02%
Biogeochemical flows - Phosphorus	P flow from freshwater to the ocean, global	9.9 Tg P yr^−^^1^		0.002%
Freshwater use	Maximum consumptive blue water use, global	4000 km^3^ yr^−^^1^		2.2%
Stratospheric ozone depletion	Stratospheric O_3_ concentration	15 DU		0.4%

For each planetary boundary (PB), the table shows the control variable, the full safe operating space (SOS) for humanity, and the relative share of SOS allocated to commercial aviation under each of the two allocation principles considered (economic-based and ethics-based, see “Methods” section).

*BII* Biodiversity Intactness Index, *DU* Dobson units.

### Key insights from the absolute environmental sustainability assessment of commercial aviation

Four key insights can be drawn from the AESA of commercial aviation. The sensitivity of these results to the choice of biofuel production pathways, assumptions on future traffic growth rate and efficiency gains, uncertainties in historical impacts, the underlying development storyline and background technosphere is assessed in Supplementary Figs. 7−11, and show that our results are robust to these variations.

**Fig. 3:**
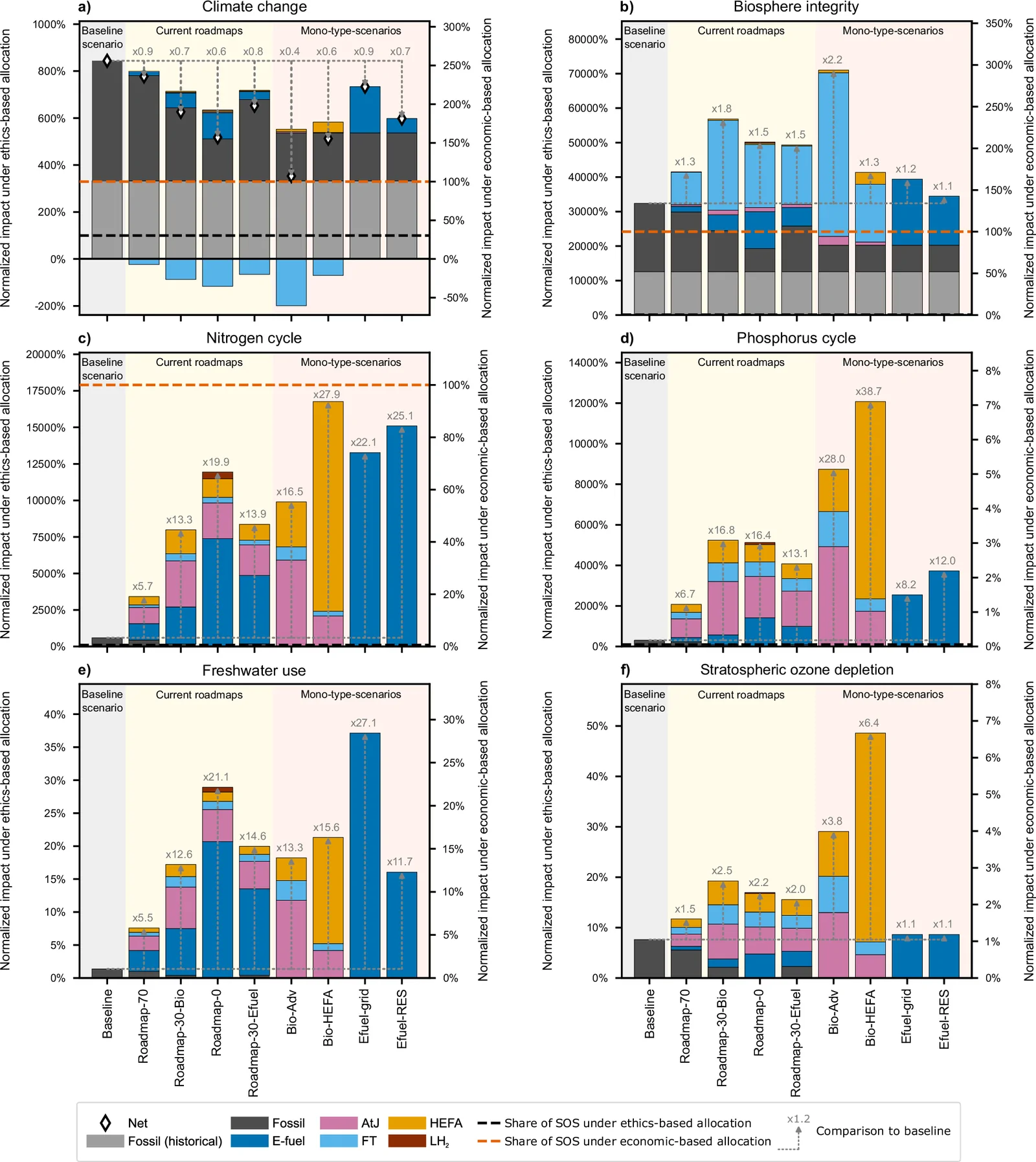
Absolute environmental sustainability assessment (AESA) across scenarios and planetary boundaries. Results of the absolute environmental sustainability assessment for the nine scenarios and six planetary boundaries (PBs) considered: **a** Climate change. **b** Biosphere integrity. **c** Nitrogen cycle. **d** Phosphorus cycle. **e** Freshwater use. **f** Stratospheric ozone depletion. The height of each bar represents the ratio between the absolute impact on the given PB and the allocated share of safe operating space for aviation. Values are reported for the ethics-based allocation on the left-axis, and for the economic-based allocation on the right-axis. Black and orange dotted horizontal lines represent the 100% threshold (black for ethics-based allocation and orange for economic-based allocation), so that a bar above a line means that the PB is transgressed for the corresponding allocation. The dotted black line for the ethics-based allocation coincides with the X-axis for (**b**), (**c**), and (**d**). In (**e**) and (**f**), the dotted lines are not represented because impacts are well below thresholds. The number reported at the top of each bar represents the ratio with the baseline fossil scenario. Reading example: impacts on biosphere integrity of the Roadmap-70 scenario are 1.28 times higher than in the baseline scenario, and represent 41 515% of the ethics-based allocation and 172% of the economic-based allocation.

First, current roadmaps are not absolutely sustainable. The respect or transgression of a PB is independent of the type of aviation roadmap and only weakly depends on the allocation principle (Fig. 3). More precisely, for both allocation principles, the climate change and biosphere integrity PBs are transgressed for all scenarios, while the stratospheric ozone depletion and freshwater use PBs are respected. The situation is more nuanced for the nitrogen and phosphorus cycles, which are respected under the economic-based allocation but transgressed under the ethics-based one. In particular, we conclude that the transgression or respect of these two planetary boundaries depends on the allocation principle considered. The transgression of the nitrogen and phosphorus cycles under the ethics-based allocation is due in part to the very small share assigned to these PBs for aviation (0.02% and 0.002%, respectively; Table 4). Nevertheless, the magnitude of the transgression is so large that they would remain transgressed even if the allocated shares were increased tenfold. Given the prime importance of climate change and biosphere integrity, these being core PBs, we thus conclude that current roadmaps are not sustainable in absolute terms. Moreover, under the ethics-based allocation, a further two PBs are transgressed substantially.

Second, historical impacts are a significant driver of the transgression of the climate change and biosphere integrity PBs. The climate change and biosphere integrity PBs deserve special scrutiny, as they have been identified as core PBs with broad and systemic interrelations^2,35^. The studied aviation roadmaps systematically transgress these two core PBs, independently of the underlying storyline, allocation principle, or biofuel production pathways considered. Under the ethics-based allocation, this is explained by the fact that historical impacts of aviation between 1940 and 2022 already result in the transgression of these PBs. Under the economic-based allocation of the safe operating space for these PBs, historical impacts also represent a major driver for the transgression, representing 103% (climate change) and 52% (biosphere integrity) of the budget allocated to aviation. Beyond 2022, fossil kerosene drives these PBs outside their allocated share, except for biosphere integrity under Roadmap-30-Bio and Roadmap-0 (Fig. 3).

This insight has potentially profound implications for the governance of the aviation sector: if aviation has exhausted its climate and biosphere integrity budgets (or is close to these limits), the question becomes how to return to the safe operating space. The discussion is akin to deploying negative emission technologies in order to reach given temperature targets, as further elaborated in the Discussion.

Third, mitigating climate change comes at the expense of increased impacts on other PBs. Beyond analyzing whether a PB is transgressed or not, the magnitude of the potential transgression is of utmost importance. For climate change, for instance, every unit of global warming matters, and this caveat also holds for other PBs^3^. In that respect, we note that the reduced climate impacts in all scenarios relative to the fossil baseline scenario come at the expense of increasing impacts for all other PBs (Fig. 3). Moreover, this impact-shifting problem is more pronounced for scenarios with a high climate mitigation potential (e.g., Roadmap-0). Conversely, less ambitious roadmaps in terms of climate mitigation (e.g., Roadmap-70), are also those with the smallest impacts on all other PBs. These results highlight the difficulty in meeting ambitious environmental goals across all PBs.

Fourth, the relative merit of efuels over biofuels depends on the electricity source and the bioenergy feedstock. Roadmap-30-Efuel has a climate impact slightly larger than Roadmap-30-Bio (by 4%), while its impacts on the other PBs are on average 10% lower. Recalling that these scenarios only differ regarding their proportion of efuels and biofuels, this suggests that efuels may be preferable from a systemic perspective, with a small deterioration of climate benefits outweighed by larger benefits for all other PBs. However, this conclusion is sensitive to the choice of biofuel pathways and electricity production method.

Indeed, when biofuel pathways are selected based on overall merits across all PBs instead of just climate change, the impacts of the Roadmap-30-Bio and Roadmap-30-Efuel become identical in terms of climate change, while the average impact on the other PBs becomes only 2% lower for Roadmap-30-Efuel than for Roadmap-30-Bio (Supplementary Fig. 8). Thus, under this choice of biofuel pathway, efuels and biofuels lead to comparable average impacts, although with a different distribution across PBs, with biofuels having greater impacts on phosphorus and stratospheric ozone depletion and less impact on biosphere integrity, nitrogen and freshwater use.

Likewise, comparing Efuel-grid and Efuel-RES shows that efuel performance is significantly affected by the electricity production method. In Efuel-grid and under the underlying development storyline considered, electricity is mostly produced from photovoltaic (56%) and wind onshore (26%) in 2050, while in Efuel-RES it is equally split between photovoltaics, wind onshore, and wind offshore. This difference in the electricity mix leads Efuel-grid to have larger impacts than Efuel-RES on freshwater use (+131%) but smaller impacts on nitrogen (−12%) and phosphorus (−32%). When the underlying development storyline is varied to capture a wider range of possible electricity production methods, the relative merits of Efuel-grid over Efuel-RES remain the same but the absolute differences increase (Supplementary Fig. 11): for instance, impacts on climate change, biosphere integrity, freshwater use and stratospheric ozone depletion remain of the same order for Efuel-RES when the underlying storyline is varied, whereas for Efuel-grid these impacts are typically doubled.

### Roadmaps with ambitious climate targets require significant amounts of resources in 2050

**Fig. 4:**
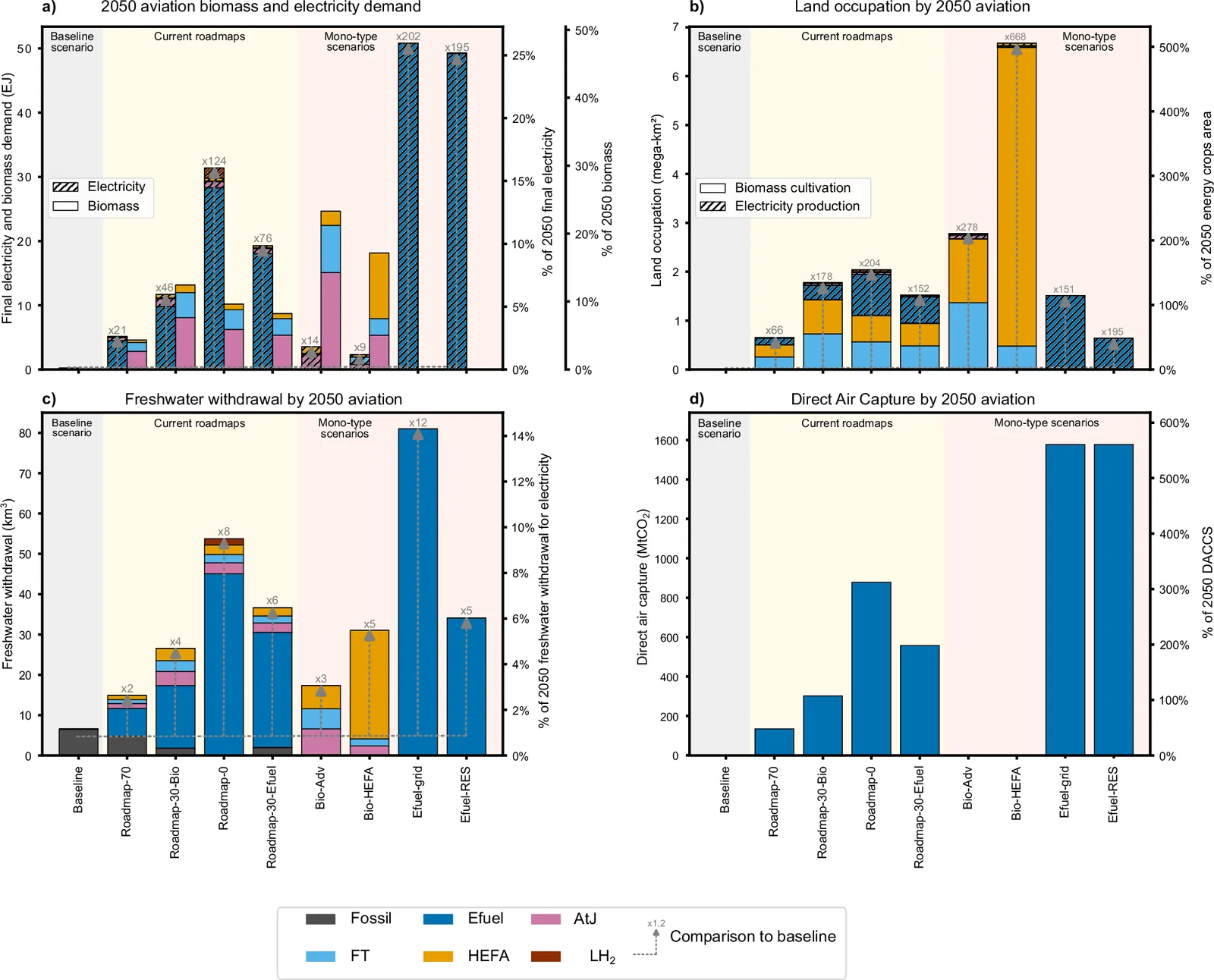
Resource requirements in 2050 across scenarios. Aviation resource requirements in 2050 are shown both in absolute values (left-hand axis) and relative values normalized by the total availability in the underlying development storyline (right-hand axis) for **a** biomass and electricity (in EJ); **b** land occupation (in Mkm²); **c** freshwater withdrawal (in km^3^); **d** Direct Air Capture (DAC, in MtCO_2_). Absolute values are normalized by the total availability in the underlying development storyline (REMIND SSP2-RCP2.6): biomass is compared to bioenergy, electricity to electricity production, land occupation to land occupation for energy crops, freshwater withdrawal to freshwater withdrawal for electricity, and DAC to DACCS (Direct Air Carbon Capture and Storage). The number reported at the top of each bar represents the ratio with the fossil baseline scenario. Reading example**:** in Roadmap-0, aviation in 2050 would require approximately 2 Mkm² of land, which represents 154% of the 2050 energy crop area in the underlying development storyline, and 204 times more land than in the baseline scenario.

Resource requirements, in terms of energy, land occupation, freshwater withdrawal and DAC, are significantly higher across all alternative aviation roadmaps to 2050 than in the baseline scenario (Fig. 4). For example, electricity demand associated with these roadmaps is increased by a factor of 20–120 and land occupation by a factor of 60–200 relative to the baseline.

To determine whether such increases represent significant challenges, we compare the absolute resource requirements to the amount of resources available in the underlying development storyline (REMIND SSP2-RCP2.6; Supplementary Fig. 5). The aviation demand for electricity, bioenergy and freshwater represents between 3 and 16% of available electricity, bioenergy and freshwater withdrawal for electricity in 2050, respectively. Specifically, the least ambitious roadmap for climate, Roadmap-70, represents 3–4% of available electricity, bioenergy and freshwater withdrawal for electricity, while the most ambitious scenario, Roadmap-0, represents 16% of electricity and around 10% of bioenergy and freshwater withdrawal for electricity. Demand for land and DAC is much more substantial and represents, on average, more land than would be dedicated to grow energy crops worldwide and more DAC than the total DACCS capacity in 2050. The least demanding scenario, Roadmap-70, is at the lower end of the spectrum but still requires half the total energy crop land area and half the total DACCS capacity. At the other extreme, the most demanding scenario, Roadmap-0, requires 1.5 times the total energy crop land area and 3 times the total DACCS capacity. Considering that aviation demand for these resources was not accounted for in REMIND SSP2-RCP2.6, this means that ambitious aviation roadmaps would simultaneously require increasing electricity production capacities by 16%, bioenergy production and freshwater withdrawal for electricity by 10%, while mobilizing a land area larger than the total area dedicated to growing energy crops, and quadrupling the DAC capacity. Varying the underlying development storyline from REMIND SSP2-RCP2.6 to other existing storylines leads to even higher relative requirements (except, in some cases, for bioenergy and land occupation for which the relative requirement can be decreased by 10%), which confirms the robustness of these findings (Supplementary Figs. 12–16).

Although we concluded above, based on systemic environmental performance, that efuels may be preferable over biofuels, we also show that aggressive efuel deployment leads to questionable resource requirements. In mono-type scenarios where all the demand is met through efuel (scenarios Efuel-grid/RES), aviation needs to mobilize around 25% of the total electricity produced and almost six times the amount of DACCS in the underlying storyline. This corroborates previous findings where efuel scenarios were studied at the European level^14^, and seriously calls into question the feasibility of current roadmaps.

### Demand-side measures can reduce the magnitude of impacts but not the number of PBs transgressed

**Fig. 5:**
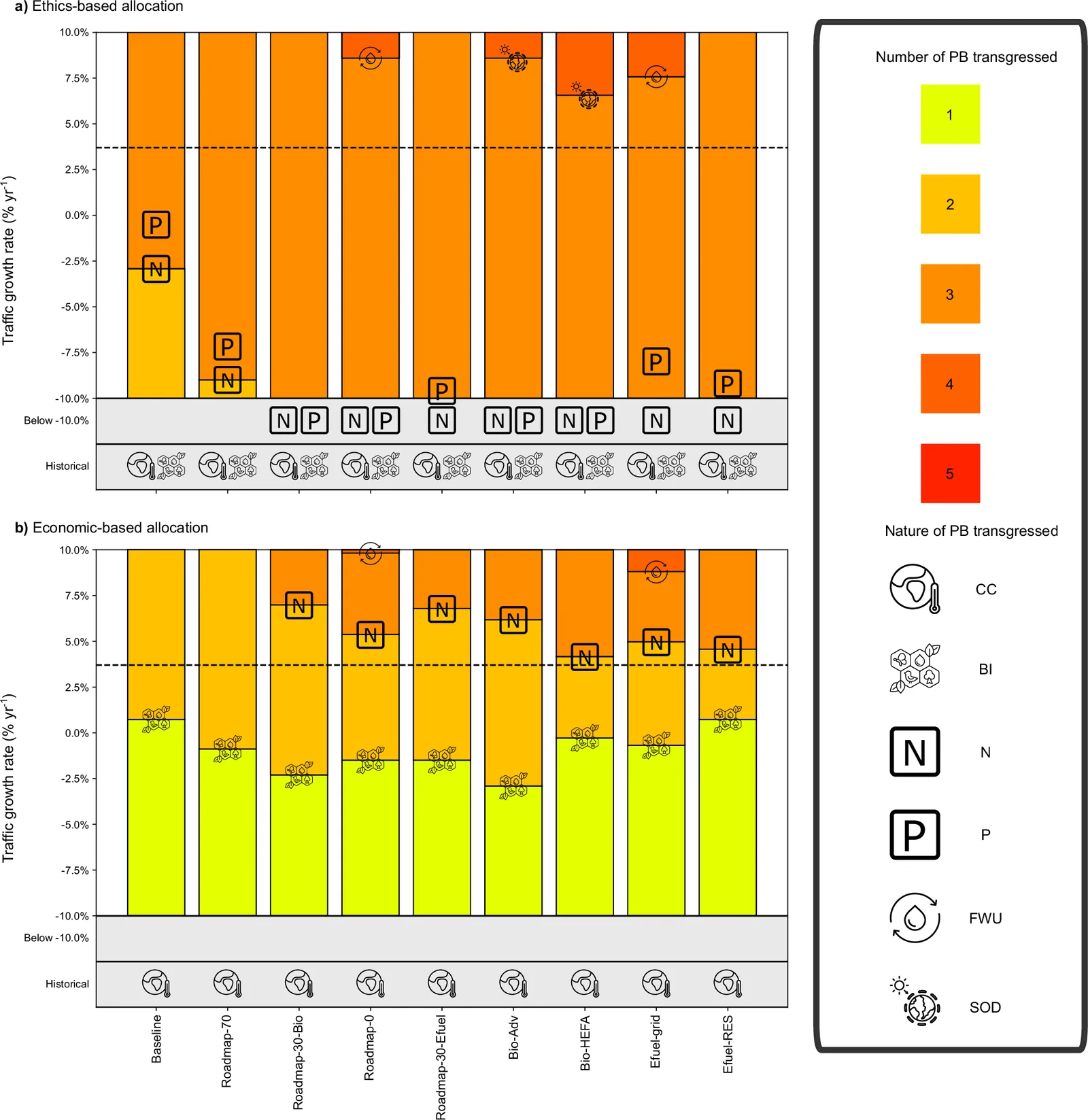
Sensitivity of the number of transgressed planetary boundaries to the traffic growth rates. The traffic growth rate is varied between −10% yr^−1^ and +10% yr^−^^1^, and the corresponding number of planetary boundaries (PBs) transgressed is indicated for each scenario under **a** the ethics-based allocation and **b** the economic-based allocation. When a traffic growth rate corresponds to the respect/transgression of an additional PB (change of color), the corresponding PB is indicated. Dashed black lines represent the baseline traffic growth used (3.7% yr^−^^1^).

Under the ethics-based allocation, no additional PB becomes respected or transgressed when traffic growth rate varies between −7.2% yr^−^^1^ and +8.6% yr^−^^1^, and at the extremes of this range, changes only occur under some roadmaps (Fig. 5). More precisely, if traffic grows at +8.6% yr^−^^1^, freshwater use becomes transgressed for Roadmap-0, and if traffic reduces by −7.2% yr^−^^1^, the phosphorus cycle PB becomes respected for Roadmap-70. Although such high growth rates are envisaged locally for some geographic regions (e.g., in Asia^26^), they are not considered likely in prospective scenarios at the global scale (Table 1). Thus, realistic growth rates higher than the value of +3.7% yr^−^^1^ used herein would only lead to increased impacts within PBs but not to the transgression of additional PBs. Similarly, highly ambitious degrowth rates of −7.2% would be required to reduce the number of PBs transgressed by the aviation sector, equivalent to a tenfold decrease of traffic by 2050. Although demand-side mitigation measures gain increasing attention in the scientific literature and in some public discussions^36^, such high degrowth rates seem unlikely even though they are of the same order of magnitude as other degrowth rates found in ambitious mitigation scenarios, for instance the annual reduction in anthropogenic CO_2_ emissions necessary to limit global warming to +1.5 °C^37^.

The situation is different under the economic-based allocation. The nitrogen cycle PB becomes transgressed for Roadmap-0 at traffic growth rates of +5.4% yr^−^^1^, while the biosphere integrity PB becomes respected under moderate degrowth rates ranging between −0.9% yr^−^^1^ for Roadmap-70 and −2.3% yr^−^^1^ for Roadmap-30-Bio.

## Discussion

A distinctive feature of the aviation sector is the large number of roadmaps and prospective scenarios produced by industrial and institutional stakeholders in recent years^11,13,25,38,39^. As new technologies have emerged, these roadmaps have become increasingly ambitious. For instance, by incorporating efuels in its 2021 roadmap, ATAG strengthened its previous goal, which was to halve CO_2_ emissions by 2050^40^, to reaching net-zero within the same timeframe^11^. These roadmaps contribute to shaping aviation governance, particularly in the case of the ICAO eventually adopting the industry’s objectives^6^. However, these roadmaps have received strong criticisms. Concerns include those relating to the historical perspectives (or lack thereof) applied in environmental analyses and aviation technological discourse, the use of industry target-setting to block stringent climate policies^41^, and evidence showing the incompatibility of SAF production with climate goals of limiting global warming to 1.5 °C^16^. By performing a systemic environmental assessment of such roadmaps, our work fills an important knowledge gap that provides critical scientific evidence to inform these debates, with significant implications for the governance of the aviation sector.

Our findings reveal that current aviation decarbonization strategies risk shifting environmental burdens from climate change to other PBs, and that they generate significant challenges in terms of resource use. This is especially true for ambitious climate roadmaps: for instance, the most ambitious roadmap analyzed for climate, Roadmap-0, reduces climate change impacts by 25% compared to the other three roadmaps considered, but at the expense of increasing impacts across all other PBs by an average of 64%. In terms of resources, this roadmap requires a more than 10% increase in electricity production, bioenergy production, and freshwater withdrawal for electricity, and further necessitates more land area to be mobilized than the total area dedicated to growing energy crops, and a quadrupling of the DAC capacity compared to the underlying storyline.

Based on our finding that no current aviation roadmap is absolutely sustainable, combined with the evidence indicating that core climate change and biosphere integrity PBs are systematically transgressed mostly due to historical impacts, we conclude that the most viable roadmaps for sustainable aviation consist of: (1) the prioritization of electricity production by renewable energy sources, which consistently demonstrate superior environmental performance; (2) a careful choice of biofuel feedstocks to optimize trade-offs across PBs, since the relative benefits of HEFA versus advanced biofuels depend on the PB and resource type considered; (3) the deployment of carbon capture and storage (CCS) technologies to compensate for historical impacts; and (4) the incorporation of demand-side mitigation measures to alleviate remaining environmental impacts and resource requirements. Designing such roadmaps requires addressing key remaining knowledge gaps, in particular, understanding how biofuel feedstocks can be adjusted in order to balance impacts across PBs, and assessing the environmental and resource implications of large-scale CCS deployment, especially regarding electricity and storage capacity, both of which warrant further investigation.

The priorities we highlight for sustainable roadmaps have potential for significant policy implications. First, they imply that aviation stakeholders should prioritize and support the deployment of renewable electricity production. Second, our results challenge the way CCS is currently considered in aviation roadmaps. Indeed, CCS has recently been considered for offsetting non-CO_2_ effects, but our results suggest that it should also be considered for offsetting historical CO_2_ emissions from aviation, since they are a significant driver for the transgression of the two core PBs. This aligns with recent evidence suggesting that the dominating global challenge has become how to navigate human activities back to within limits rather than simply stabilizing the Earth system. However, the mere requirement of carbon capture to produce efuels is already very significant, and so requiring more carbon capture to offset historical impacts exacerbates the pressure on accelerating DAC deployment.

While our finding that current aviation roadmaps are not sustainable aligns with that of Sacchi et al.^14^ at the European level, it challenges the recent results of Millward-Hopkins et al.^42^. The authors proposed a bottom-up global scenario, grounded in the theory of human needs, that aims to provide decent living to every human being with minimum energy consumption. In this scenario, air travel amounts to 1000 km.cap^−^^1^.yr^−^^1^, which is close to the current average distance flown per capita. This suggests that current levels of air traffic are within safe environmental boundaries, but our results indicate otherwise, as even with constant traffic, none of our scenarios can be considered sustainable in absolute terms. A stepping stone toward flying within PBs therefore lies in deepening this understanding of how behavioral changes can complement energy transitions in reducing aviation’s environmental impacts.

The transgression of the climate change PB is all the more significant because our work underestimates climate impacts: non-CO_2_ effects are not taken into account, and we selected the climate-optimal production pathways without considering physical and economic constraints that would limit feedstock availability. Accounting for non-CO_2_ effects would not change our conclusions regarding the transgression of the core planetary boundaries, since they would only increase impacts. However, quantifying the magnitude of the transgression would help in assessing the amount of carbon capture technologies needed to offset aviation’s climate impact and reach climate goals. Quantifying non-CO_2_ effects in prospective scenarios faces significant challenges because these effects depend on spatially-specific traffic distribution^43^ and on the evolution of ambient atmospheric composition^44^. Furthermore, the impacts of alternative energy carriers on non-CO_2_ effects currently face significant uncertainties^45^. Thus, addressing these limitations presents a key avenue for future research.

We note finally that, as in any AESA, our results depend on the allocation methods considered. By considering two methods rooted in different principles, our results cover a large span of possible allocations, which makes them robust, but there are still shortcomings. First, both approaches suffer from the issue of being centered in the status quo, whereby the current situation is used for allocation in the future. Especially for the ethics-based approach, this can neglect the potential development of new technologies. Moreover, the two approaches ultimately rely on metrics (gross domestic product [GDP] for the economic-based approach and revenue-passenger kilometer [RPK] for the climate change allocation in the ethics-based approach) designed to be proxies of a notion of “utility” for the aviation sector. However, these metrics are debatable: GDP does not reflect the necessity or equity of different economic activities, while RPK merely measures the scale of transport activity, ignoring key aspects such as travel purpose, substitutability, and distributional inequality. In a sector like aviation, the use and benefits of which are highly unequal and partially characterized by luxury demand, these indicators may systematically overestimate its justifiable environmental share. Therefore, designing allocation principles that account for these key aspects also represents an important research direction.

## Methods

### Production pathway selection

The production pathway (i.e., combination of feedstock, including electricity sources, and conversion pathway) for a given alternative energy carrier can have a large influence on its environmental performance. However, information on the precise production pathways is largely missing from the ICAO, ATAG and EU roadmaps. To fill this gap, an exhaustive literature overview was performed, leading to the selection of 123 suitable life-cycle inventories (LCIs) corresponding to 123 potential production pathways: 6 for HEFA, 10 for FT, 7 for AtJ, 84 for efuels and 16 for LH_2_ (Supplementary Tables 1–3). Whilst there is abundant life-cycle assessment (LCA) literature on alternative energy carriers’ environmental performance, more comprehensive LCIs are needed to assess impacts at the PB level. Most available LCAs focus on climate impacts and rely on partial LCIs, which makes these inventories unsuitable for our study. In particular, no exhaustive LCI was found for the lower-carbon aviation fuel considered in the ICAO LTAG scenarios, which explains why it was not considered in the present study.

The PB-LCIA methodology^21^ was then applied to these LCIs (Supplementary Fig. 2), and production pathways were then selected based on these results. For LH_2_ and efuel, we considered H_2_ produced from water electrolysis (proton exchange membrane). For efuels, we considered CO_2_ from direct air capture with a solvent-based system. For all the scenarios except Efuel-RES, in which electricity is produced in equal shares from photovoltaics, wind onshore and wind offshore, electricity reflects the world grid for which the characteristics are governed by the underlying development storyline. For biofuels (HEFA, FT, and AtJ), we considered two choices: one that minimized climate impacts and one that balanced impacts across PBs (Supplementary Tables 4 and 5).

### Dynamic AESA methodology

**Fig. 6:**
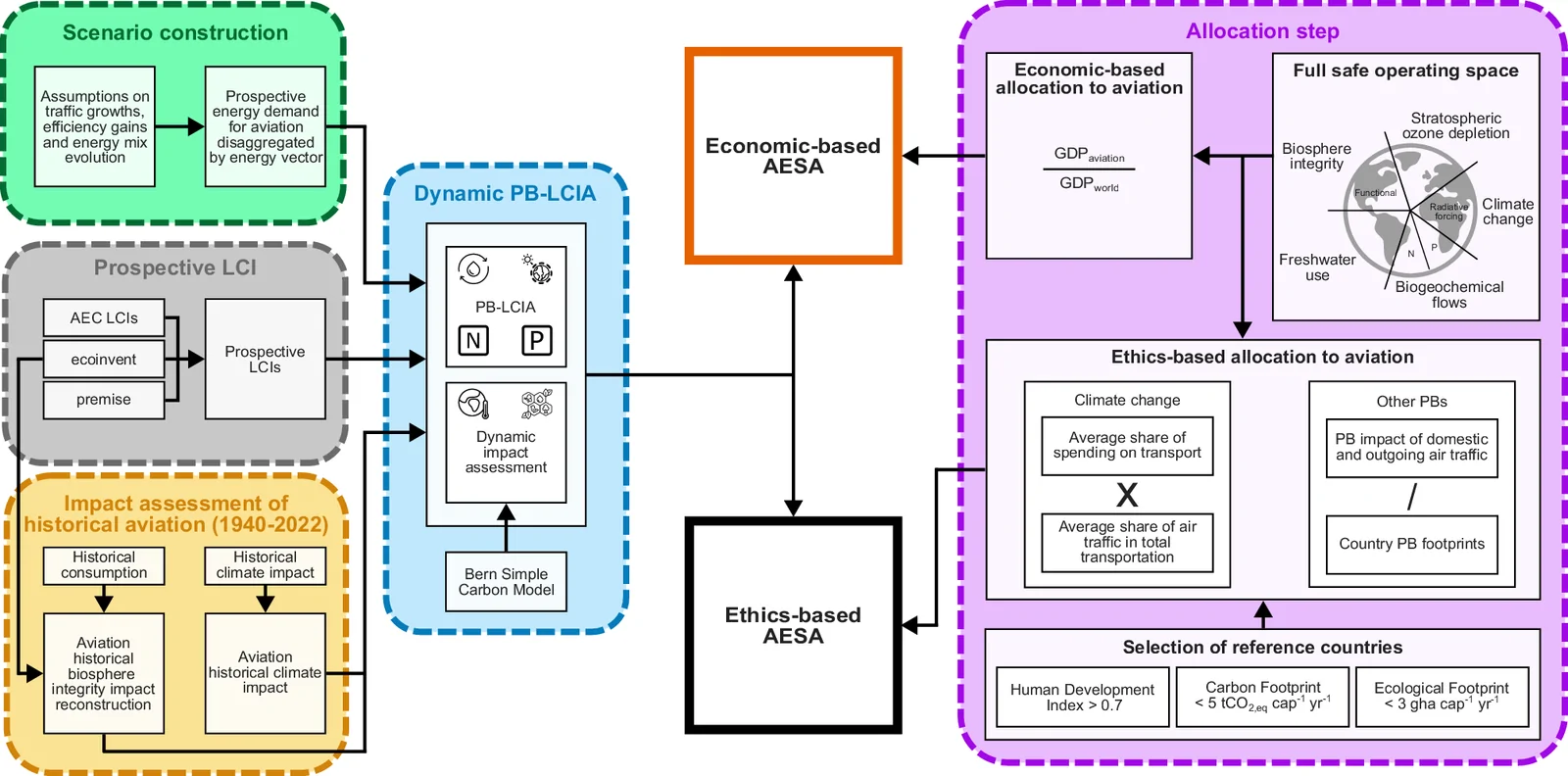
Graphical description of the modeling framework. The modeling framework combines four main steps to produce the final absolute environmental sustainability assessment (AESA). Prospective life-cycle inventories (LCIs) are built for each alternative energy carrier (AEC) considered in the study. These LCIs are combined with the aviation scenarios to compute absolute environmental impacts using the developed dynamic PB-LCIA methodology. In parallel, a share of the safe operating space for each planetary boundary (PB) is allocated to the aviation sector using two allocation principles: an ethics-based allocation (based on the fulfillment of human needs methodology^22^) and an economic-based allocation (based on the sector’s contribution to global economic activity through gross domestic product [GDP]). The final AESA results from the comparison between the absolute impacts and the allocated shares.

AESA consists of three steps (Fig. 6). First, the impact on each PB is assessed. Second, a downscaling step assigns to each PB a share of the safe operating space for the sector under study. Third, the normalized impact (i.e., the absolute impact divided by the full safe operating space) is compared to the allocated share.

For the impact assessment step, we built on the PB-LCIA methodology, which we extended (compared to the original publication^21^) in several directions by: (i) adapting it to a dynamic setting; (ii) assessing impacts on biosphere integrity in this dynamic context; (iii) accounting for historical impacts; (iv) coupling it with prospective LCIs. More precisely, in the original publication, PB-LCIA was designed to evaluate a static situation (in particular, historical impacts were not accounted for) and biosphere integrity was not included in the analysis^21^. Our main methodological contribution is to extend this framework so that it can be applied to the analysis of dynamic scenarios in other sectors. This methodological development relies on the use of the Bern simple carbon model for climate change, and the extension of the approach of Galan-Martin et al.^46^ to include biosphere integrity in this dynamic context (see “Dynamic PB-LCIA with biosphere integrity” section). Analyzing a dynamic trajectory allows for the incorporation of historical impacts in the assessment (see “Impact assessment of historical aviation (1940–2022)” section). For the prospective range 2023–2050, we strengthened its consistency by coupling it with prospective LCIs (see “Prospective LCIs (2023–2050)” section). We finally detail how to compute allocated shares under the economic and ethics-based allocation principles (see “Computing the ethics-based allocated shares” section). Note that, despite the inclusion of biosphere integrity, our results do not cover the ocean acidification and land-system change PBs for reasons elaborated on below.

### Dynamic PB-LCIA with biosphere integrity

Characterization factors of the PB-LCIA methodology were developed to assess the absolute environmental sustainability of an activity remaining stable until 2300^21^. The time range does not matter for flow PBs, and so these characterization factors can be used to assess impacts of a dynamic scenario for such PBs, namely nitrogen and phosphorus cycles, stratospheric ozone depletion and freshwater use. However, they are unsuitable for cumulative PBs such as land-system change, climate change, ocean acidification and biosphere integrity.

To assess impacts on climate change in this dynamic context, we use the approach of Ryberg et al.^47^ based on the Bern simple climate model (Supplementary Note 2). Note that non-CO_2_ effects are not taken into account in this study, which means that these results should be considered as an underestimation of aviation’s climate impacts^48^.

As we did not find a corresponding short-term dynamic model for ocean acidification, we do not assess impacts on this PB. Moreover, to the best of our knowledge, data is not available to compute the ethics-based share of the safe operating space for this PB (see “Computing the ethics-based allocated shares” section). However, it is important to note that ocean acidification is strongly correlated with climate change^49,50^.

Biosphere integrity is not assessed in the original PB-LCIA publication, but characterization factors were later developed by Galan-Martin et al.^46^. These characterization factors result from a combination of impacts on climate change and on direct land use, which are two of the main drivers for biodiversity loss. To assess impacts on biosphere integrity in a dynamic context, we use the same approach as Galan-Martin et al.^46^ but replace the impact assessment step on climate change by the Bern simple climate model (Supplementary Note 3).

Impacts on land-system change are not assessed due to highly uncertain LCIs. While land occupation data are generally robust and consistent for specific technologies or processes, translating these into impacts on land-system change such as deforestation requires assumptions about land type, location, and baseline conditions that are highly uncertain at the global level. Partial data to link land occupation to deforestation is available for photovoltaic farms, but it is not enough to develop a full analysis.

### Impact assessment of historical aviation (1940–2022)

The climate change PB has already been transgressed at the global level, and therefore a central question becomes how to allocate shares of a safe operating space that no longer exists. In the classical PB-LCIA method, this question is addressed by performing the analysis over a longtime range (up to 2300) and assuming that climate change will go back to its safe operating space in the meantime, leaving room in the future for activities to share this renewed safe operating space^35^. As our goal is to assess the sustainability of current roadmaps on shorter time scales, this approach would not be suitable. Instead, we develop a backward-looking approach by assessing the impact of commercial aviation since 1940 and comparing it to the downscaled preindustrial safe operating space. This historical approach is consistent with the standard climate assessment method through radiative forcing, which also accounts for historical impacts^48^. It is also common in the literature on sharing carbon budgets^51^ as it complies with the ethical principle of differentiated responsibility^10^. Within this backward-looking approach, it is logical only to assess historical impacts on the two cumulative PBs considered in the dynamic PB-LCIA method developed (section above), namely climate change and biosphere integrity. However, since the cumulative nature of the biosphere integrity PB is only due to its climate component (whereas direct land use, the other component of the control variable, is non-cumulative), only historical climate impacts need to be considered.

The past energy demand of aviation between 1940 and 2018 is given in Lee et al.^48^, while for the 2019–2022 period we rely on data from the International Air Transport Association (IATA)^52^. The pre-2018 data poses some methodological challenges in terms of assessing impacts of comparable scope to those in the rest of the paper. Historical data has greater coverage in some respects as it includes military and private aviation alongside commercial aviation. On the other hand, these historical data lack emissions associated with kerosene production, which makes it difficult to assess emissions across the whole life-cycle. Notwithstanding this concern, data suggests that these two contradictory effects are of the same order of magnitude and thus can be considered to roughly cancel each other out, with military and private aviation representing around 20% of global aviation emissions^8^ and emissions from kerosene production also representing around 20% of emissions from combustion^53^. Sensitivity analysis performed in the SI suggests that our results are robust to these uncertainties in historical impacts (Supplementary Fig. 10).

### Prospective LCIs (2023–2050)

In the prospective range 2023–2050, the consistency of the dynamic PB-LCIA method is strengthened by using prospective LCIs^30^. These LCIs are built from ecoinvent 3.9.1 using the premise (v2.2.0) package^30^, which adjusts the background life-cycle database to align it with the socioeconomic and climate mitigation assumptions of a given SSP-RCP (Shared Socioeconomic Pathway and Representative Concentration Pathway), as modeled by an Integrated Assessment Model (IAM). This ensures that the resulting inventories are consistent with the selected scenario’s projections. Thanks to this approach, our results account for future improvements in the background technosphere and how this influences the environmental performance of alternative energy carriers (efficiency of processes, carbon intensity of electricity production, etc.). This choice of IAM and SSP-RCP scenario is referred to in this paper as the (underlying) development storyline.

Besides REMIND SSP2-RCP2.6, three other development storylines are considered in the SI: REMIND SSP2-NPi, REMIND SSP1-RCP1.9 and IMAGE SSP2-RCP2.6. Results in the main text are presented for REMIND SSP2-RCP2.6 because SSP2-RCP2.6 projects nondisruptive socioeconomic trends and a climate goal consistent with the objectives of the Paris Agreement^10^, and because REMIND models a shift in the electricity mix toward a majority of renewables (photovoltaic and wind, both onshore and offshore) by 2050, which aligns with the assumptions of current roadmaps on the source of electricity used. Compared to REMIND, the IMAGE IAM brings relevant information as the electricity mix defossilizes more slowly (Supplementary Fig. 6).

### Computing the ethics-based allocated shares

The ethics-based allocation implements the fulfillment of human needs (FHN) principle. FHN operationalizes the sufficientarianism principle of distributive justice theory (everyone should get enough) by allocating environmental burdens according to how essential the needs satisfied by different products and sectors are to users. For the computation of the ethics-based shares, we followed the original description of the fulfillment of human needs principle^22^, which required us to leverage new data. First, we selected reference countries in terms of their social and environmental sustainability, assessed based on whether they simultaneously met three thresholds: a human development index above 0.7, an ecological footprint below 3 gha cap^−^^1^ yr^−^^1^, and a carbon footprint below 5 tCO_2_eq cap^−^^1^ yr^−^^1^.

As explained in the original publication^22^, those thresholds correspond to proposed values for sustainability (for instance, a human development index above 0.8 corresponds to the “very high human development group”), which have been relaxed in order to get a sufficient number of countries simultaneously passing the bar for the three indicators. These criteria lead to the selection of 24 countries (Supplementary Table 7). It has to be noted that these countries are not representative of countries worldwide, nor do they capture changes occurring in the aviation industry. Rather, the selection process from the three social and environmental indicators aims to get countries representative of ‘sustainable’ countries, from a social and environmental perspective. The resulting reference countries tend to be developing countries due to the fact that developed countries usually fail to satisfy both environmental criteria: the performance of developed vs developing countries is analyzed in more detail in Supplementary Fig. 18. From these reference countries, two approaches were considered to compute allocated shares: a specific approach for climate change and another approach for the other PBs. This distinction follows the original framework of Heide et al.^22^, who propose an expenditure-based sharing for climate change because CO₂ emissions are strongly correlated with consumption across most sectors, while impacts on the other planetary boundaries exhibit large sectoral heterogeneity and therefore require impact-based sharing to reflect these differences.

The share for climate change is given as Eq. (1):

1 $${{\rm{S}}}{{{\rm{hare}}}}_{{{\rm{CC}}}}={{{\rm{SC}}}}_{{{\rm{mean}}},{{\rm{transport}}}}*\frac{{{{\rm{RPK}}}}_{{{\rm{aviation}}}}}{{{{\rm{RPK}}}}_{{{\rm{transport}}}}}$$

where ${{{\rm{SC}}}}_{{{\rm{mean}}},{{\rm{transport}}}}$ is the mean share of household and government spending allocated to transport, where the average is taken over the reference countries, and ${{{\rm{RPK}}}}_{{{\rm{aviation}}}}/{{{\rm{RPK}}}}_{{{\rm{transport}}}}$ represents the proportion of Revenue-Passenger Kilometer realized through air transport (Supplementary Note 7). This Share_CC_ share is meant to capture the utility of the aviation sector to individuals. For climate change, the original FHN publication recommends computing the share based on household and government spending. However, these data are only available for the whole transport sector (which facilitates computation of the term ${{{\mathrm{SC}}}}_{{{\mathrm{mean}}},{{\mathrm{transport}}}}$) and so the ratio ${{{{\rm{RPK}}}}}_{{{{\rm{aviation}}}}}/{{{{\rm{RPK}}}}}_{{{{\rm{transport}}}}}$ is here to evaluate the proportion of this share that is allocated to the aviation sector specifically, within the larger transport sector. Considering RPKs for this is in line with the aim of capturing utility to individuals, since for the transport sector, this metric can be seen as the end-product delivered.

For the other PBs, the share is obtained as an average over the reference countries of the relative impact of aviation on each PB for each country. Thus, the share of a given PB is obtained as an average over countries i of the values given as Eq. (2) (a sensitivity analysis based on the dispersion around the mean is conducted in Supplementary Fig. 19):

2 $${{{\rm{S}}}}{{{\rm{hare}}}}_{i,{{{\rm{PB}}}}}=\frac{{{{\rm{Impact}}}}_{i,{{\rm{aviation}}},{{{\rm{PB}}}}}}{{{{\rm{Impact}}}}_{i,{{\rm{PB}}}}}$$

with ${{{{\rm{Impact}}}}}_{i,{{{\rm{aviation}}}},{{{\rm{PB}}}}}$ the impact of aviation of country i on given PB and ${{{{\rm{Impact}}}}}_{i,{{{\rm{PB}}}}}$ the global impact of country i on the given PB. To compute ${{{{\rm{Impact}}}}}_{i,{{{\rm{aviation}}}},{{{\rm{PB}}}}}$, we run the standard PB-LCIA methodology on the 2019 air traffic of country i (Supplementary Table 8). To compute ${{{{\rm{Impact}}}}}_{i,{{{\rm{PB}}}}}$, Heide et al.^22^ recommend using the input-output data from EXIOBASE. However, only one of the 24 selected countries has available data in EXIOBASE, and only on three PBs (nitrogen, freshwater, and land-system change)^33^. We fill those gaps by conducting bibliographic research for input-output footprint data for each PB and for each country (Supplementary Table 7). Note that all the shares computed above are constant with time: considering dynamic allocation aligned with assumptions of the underlying storyline could be interesting to ensure consistency with the prospective LCA approach. However, no such allocation schemes have been proposed to date, and so this represents an interesting venue for future research.

### Resource analysis

To assess resource requirements, we calculate the prospective LCI for each underlying development storyline. The amount of electricity and bioenergy required in 2050 is calculated using the corresponding LCIs and is then compared to the amount of electricity (secondary energy) and bioenergy (primary energy) in the storyline^54^.

To evaluate the land occupation associated with aviation-related electricity production, we extract from the corresponding LCI the required amount of electricity disaggregated by the production method. We then use the work of van Zalk and Behrens^55^ to calculate the land occupation linked to this electricity production using power density values. A similar approach is applied to biomass cultivation: we extract the quantity of bioenergy used by each scenario in 2050 from the LCI and multiply it by the surface yield of the corresponding crop^56,57^. The results are compared with the 2050 energy crop area^54^.

The evaluation of freshwater withdrawal complements the assessment of the freshwater use PB. Evaluating freshwater withdrawal allows us to assess the demand for freshwater and compare it to the overall availability. Results are obtained directly from the corresponding LCI and are compared with freshwater withdrawal for electricity^58^.

Scenarios using efuels require Direct Air Carbon Capture and Usage (DACCU), which can potentially compete with Direct Air Carbon Capture and Storage (DACCS)^59^ used in integrated scenarios to meet climate targets. The amount of DACCU in each aviation scenario (the CO_2_ captured and used as feedstock for efuels) is compared with the DACCS capacity in the underlying storyline^54^.

## Supplementary information

Supplementary Information

Transparent Peer Review file

## Data Availability

All data supporting the findings of this study and the numerical values underlying Figs. 2–5 and all Supplementary Figs., are openly available in the Zenodo repository at https://doi.org/10.5281/zenodo.14186426^60^. Source data for Figs. 2–5 and Supplementary Figs. are also provided with this paper. The data are released under a Creative Commons Attribution 4.0 International license (CC BY 4.0). The aviation traffic data, the Integrated Assessment Model outputs used to build the prospective technosphere, and the country-level environmental data used to compute the FHN allocation shares are all obtained from openly available third-party sources. Each of these sources is cited in the “Methods” section and Supplementary Information, with the corresponding references and access URLs given in the reference list.The ecoinvent life-cycle inventory database (v3.9.1) used as the background inventory in this study is available under restricted access from the ecoinvent Association (https://ecoinvent.org/). Access requires a paid license; the licensing terms and access procedures are described on the ecoinvent website. This restriction is imposed by the third-party data provider and is not under the authors’ control.

## Code availability

All code used to perform the analyses and generate the figures of this study is openly available in the same Zenodo repository^60^. The code is released under the MIT license.
