# Supplementary material for: Current aviation roadmaps are not within planetary boundaries
Source: Nat Commun. 2026 Oct 13;17:10736. doi: 10.1038/s41467-026-77472-6 (PMC13662196; doi:10.1038/s41467-026-77472-6)
Supplement: Supplementary file 1 [file 41467_2026_77472_MOESM1_ESM.pdf]

Supplementary Information for

Current aviation roadmaps are not within planetary boundaries

Bastien Païs[1,2], Alexandre Gondran[1,3], Lorie Hamelin[4], Florian Simatos[1*]

[1]Fédération ENAC ISAE-SUPAERO ONERA, Université de Toulouse, France

[2]Agence de l'environnement et de la Maîtrise de l'Energie, 20, avenue du Grésillé- BP 90406 49004 Angers Cedex 01 France

[3]ENAC, French Civil Aviation University, Université de Toulouse, Toulouse, France

[4]TBI, Université de Toulouse, CNRS, INRAE, INSA, Toulouse, France

*Corresponding author: florian.simatos@isae.fr

Contents

Supplementary notes

Supplementary Note 1 - Acronyms 4

Supplementary Note 2 - Dynamic climate change impact assessment........ 10

Supplementary Note 3 - Dynamic biosphere integrity impact assessment........ 11

Supplementary Note 4 - Precision on land-related metrics 13

Supplementary Note 5 - Prospective Technosphere 13

Supplementary Note 6 - Comparison of aviation traffic assumptions with IAM scenarios 16

Supplementary Note 7 - Sharing the SOS: Fulfilement of Human Needs 28

Supplementary figures

Supplementary Figure 1 - Mix evolution between 2023 and 2050 in current roadmaps. 5

Supplementary Figure 2 - Impacts of AECs relative to impacts of fossil kerosene 8

Supplementary Figure 3 - Evolution of radiative forcing between 2023 and 2050. 11

Supplementary Figure 4 - Evolution of biosphere integrity impact between 2023 and 2050. 12

Supplementary Figure 5 - Evolution of resources availability in the considered IAMs. 15

Supplementary Figure 6 - Evolution of electricity production in the considered Integrated Assement Models (IAMs). 16

Supplementary Figure 7 – Monte Carlo uncertainty analysis of the AESA results. 17

Supplementary Figure 8 - Sensitivity analysis of AESA to the biofuel selection. 18

Supplementary Figure 9 - Sensitivity analysis of AESA to traffic growth rates and efficiency gains. 19

Supplementary Figure 10 - Sensitivity analysis of AESA to historical impact uncertainties. 20

Supplementary Figure 11 - Sensitivity analysis of AESA to underlying development storyline. 21

Supplementary Figure 12 - Sensitivity analysis of 2050 aviation absolute resource requirements to underlying development storyline........ 22

Supplementary Figure 13 - Sensitivity analysis of 2050 aviation relative resource requirements to underlying development storyline........ 23

Supplementary Figure 14 - Sensitivity analysis of 2050 aviation relative resource requirements to the biofuel selection........ 24

Supplementary Figure 15 - Sensitivity analysis of 2050 aviation absolute resource requirements to the biofuel selection........ 25

Supplementary Figure 16 - Sensitivity analysis of 2050 aviation absolute resource requirements to traffic growth rates and efficiency gains. 26

Supplementary Figure 17 - Estimates of impacts on climate change and biosphere integrity when contrail effects are taken into account by applying factors of Lee et al. 27

Supplementary Figure 18 - Performance of countries worldwide on the three indicators used for selecting reference countries in FHN. 31

Supplementary Figure 19 - Sensitivity analysis of the FHN share allocated to aviation........................... 32

Supplementary tables

Supplementary Table 1 - List of carbon capture pathways considered. 6

Supplementary Table 2 - List of hydrogen production pathways considered. 6

Supplementary Table 3 - List of biofuel Life Cycle Invetories (LCIs) found in the literature. 7

Supplementary Table 4 - Ranking of biofuel environmental performances by pathway. 9

Supplementary Table 5 - Biofuel selection used in the main analysis (climate-based) and in the sensitivity analysis (PB-based). 10

Supplementary Table 6 – Comparison of current roadmaps and Integrated Assessment Models (IAMs) assumptions. 17

Supplementary Table 7 - Footprint data for each PB and for each reference country. 29

Supplementary Table 8 - 2019 traffic by country (domestic + flights departing from the country). 30

Supplementary Table 9 - Share of household and government spendings allocated to the transport sector in the reference countries, adjusted with the food share from EAT-Lancet report. 31

Supplementary references

Supplementary References.. 33

Supplementary Note 1 - Acronyms

AEC=Alternative Energy Carriers

AESA=Absolute Environmental Sustainability Assessment

ATAG=Air Transport Action Group

AtJ=Alcohol-to-Jet

CR=Current Roadmaps

DAC=Direct Air Capture

EU=European Union

FHN=Fulfilment of Human Needs

FT=Fischer-Tropsch

GDP=Gross Domestic Product

HEFA=Hydroprocessed Esters and Fatty Acids

IAM=Integrated Assessment Model

ICAO=International Civil Aviation Organization

LCA=Life Cycle Assessment

LCAF=Lower Carbon Aviation Fuel

LCI=Life Cycle Inventory

LCIA=Life Cycle Impact Assessment

LH_2=Liquefied hydrogen

MTS=Mono-Type Scenario

PB=Planetary Boundary

PEM=Proton Exchange Membrane

RCP=Representative Concentration Pathway

RES=Renewable Energy Sources

SOS=Safe Operating Space

SSP=Shared Socioeconomic Pathway

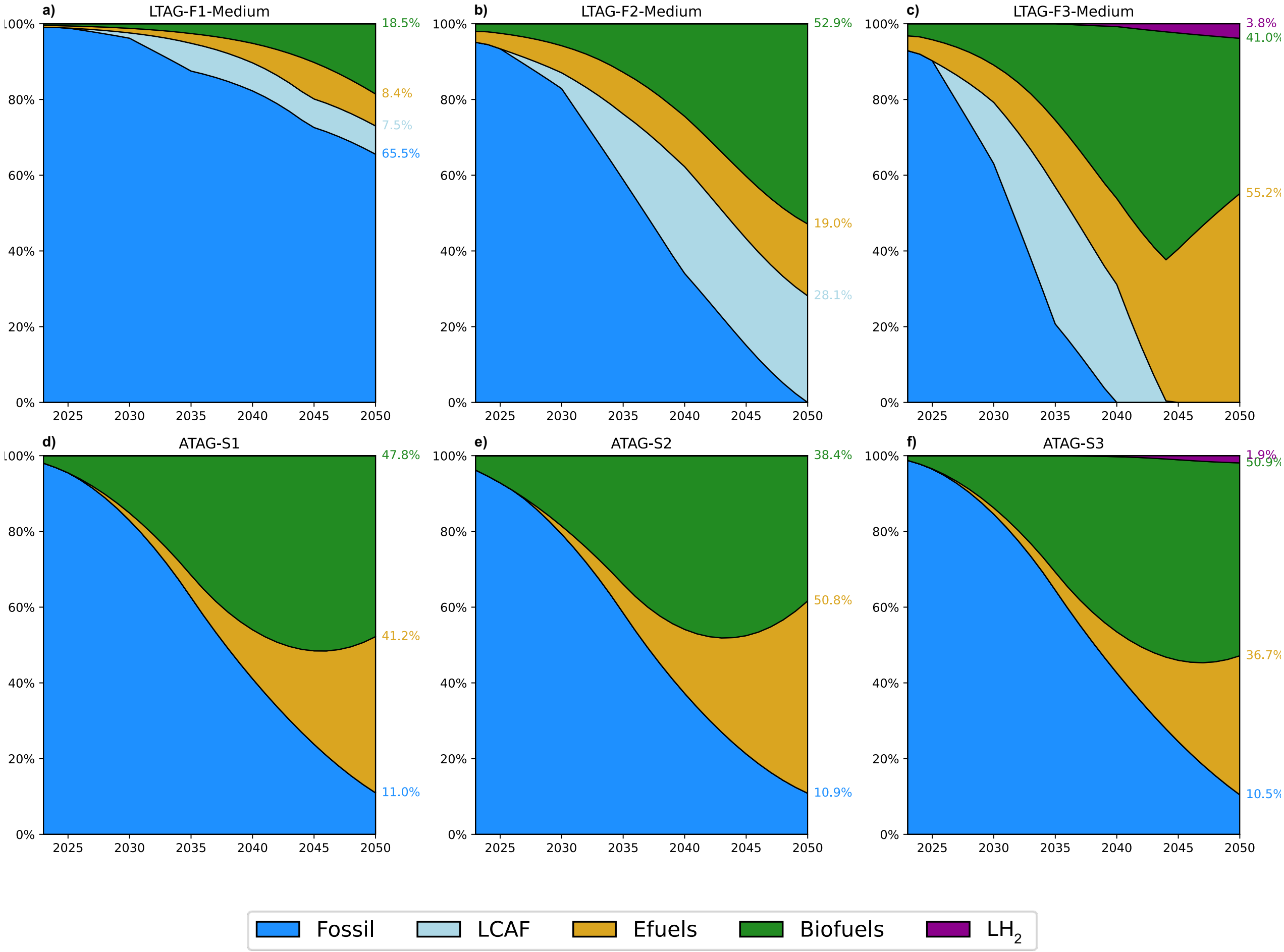

Supplementary Figure 1 - Mix evolution between 2023 and 2050 in current roadmaps.
Each panel represents the relative composition (in percentage) of the fuel mix used by aviation from 2019 to 2050 for different scenarios proposed by different aviation stakeholders: the International Civil Aviation Organization (ICAO) and the Air Transport Action Group (ATAG): **a**. ICAO LTAG-F1-Medium; **b**. ICAO LTAG-F2-Medium; **c**. ICAO LTAG-F3-Medium; **d**. ATAG S1; **e**. ATAG S2; **f**. ATAG S3. This figure complements Figure 2 in the main text in two ways: (i) it displays the ATAG scenarios and (ii) although the ICAO roadmaps correspond to Roadmap-70, Roadmap-30-Bio and Roadmap-0 in the main text (Table 2), they bring additional information by displaying the evolution of LCAF which, in the main text, is assimilated with fossil kerosene. The data for the ICAO scenarios are directly available on their website[1]. These scenarios consider international commercial aviation. The data for the ATAG scenarios were reconstructed from the 2nd "Waypoint 2050" report[2] and the "Fueling net zero" report[3] for use in the AeroMaps tool[4]. These scenarios consider the entire commercial aviation. The distribution between biofuel types (FT, AtJ and HEFA) is based the Waypoint 2050 scenarios[2,3], which project that approximately 85% of biofuels will be produced from advanced feedstocks, with the remaining 15% from the HEFA pathways. Advanced feedstocks encompass both FT and AtJ pathways; their relative shares are assumed to be evenly split for the purposes of this study.

Supplementary Table 1 - List of carbon capture pathways considered.
Direct Air Capture (DAC) with solvent-based system (with both grid and RES [Renewable Energy Sources] electricity depending on the scenario considered) is selected for the scale-up.

Pathway	DAC system	Electricity	Hydrogen	Reference
DAC	Sorbent-based	Grid	See Supplementary Table 2	Qiu et al.[5]
DAC	Sorbent-based	RES		
DAC	Solvent-based	Grid		
DAC	Solvent-based	RES		
Captured at cement production plant	-	-	-	Meunier et al.[6]
Capture at municipal solid waste incineration plant	-	-	-	Bisinella et al.[7]

Supplementary Table 2 - List of hydrogen production pathways considered.
Proton Exchange Membrane (PEM) electrolysis (with both grid and RES [Renewable Energy Sources] electricity depending on the scenario considered) is selected for the scale-up.

Pathway	Feedstock	CCS	Electricity	Comment	Reference
Electrolysis	Water	-	Grid	Alkaline Electrolysis Cell	Gerloff et al.[8]
Electrolysis	Water	-	RES	Alkaline Electrolysis Cell	
Electrolysis	Water	-	Grid	Proton Exchange Membrane (PEM)	
Electrolysis	Water	-	RES	PEM	
Electrolysis	Water	-	Grid	Solid Oxide Electrolyzer Cell (SOEC)	
Electrolysis	Water	-	RES	SOEC	
Gasification	Wood	No	-	-	Antonini et al.[9]
Gasification	Wood	Yes	-	-	
Pyrolysis	Natural gas	-	-	-	Al-Qahtani et al.[10]
Steam Methane Reforming (SMR)	Natural gas	No	-	-	Antonini et al.[11]
SMR	Natural gas	Yes	-	-	
SMR	Biogas	No	-	-	
SMR	Biogas	Yes	-	-	
Thermochemical water splitting	Water	-	-	-	Zhang et al.[12]

Supplementary Table 3 - List of biofuel Life Cycle Invetories (LCIs) found in the literature.
These LCIs were selected from an exhaustive literature overview based on the following criteria: the LCIs need to be exhaustive, modeled following the attributional approach, recent (published since 2020) and open source.

Feedstock	Reference
**Alcohol-to-Jet (AtJ)**	
Sawdust	Björnsson et al.[13]
Sugarcane residues	Capaz et al.[14]
Forestry residues	Capaz et al.[14]
Forestry residues	Bedin G.[15]
Corn cob	Wang et al.[16]
Corn cob	Wang et al.[16]
Corn cob	Wang et al.[16]
**Fischer-Tropsch (FT)**	
Pinewood	Almena et al.[17]
Logging residues	Björnsson et al.[13]
Sugarcane residues	Capaz et al.[14]
Forestry residues	Capaz et al.[14]
Forestry residues	Cavalett et Cherubini[18]
Forest products	Cavalett et Cherubini[18]
Forestry residues	Rojas Michaga et al.[19]
Forestry residues	Lai et al.[20]
Black liquor	Lai et al.[20]
Sugarcane bagasse	Real Guimaraes et al.[21]
**Hydroprocessed Esters and Fatty Acids (HEFA)**	
Used cooking oil	Barbera et al.[22]
Used cooking oil	Capaz et al.[14]
Beef tallow	Capaz et al.[14]
Jatropha	Liu et al.[23]
Castor	Liu et al.[23]
Pennycress	Mousavi-Avval et al.[24]

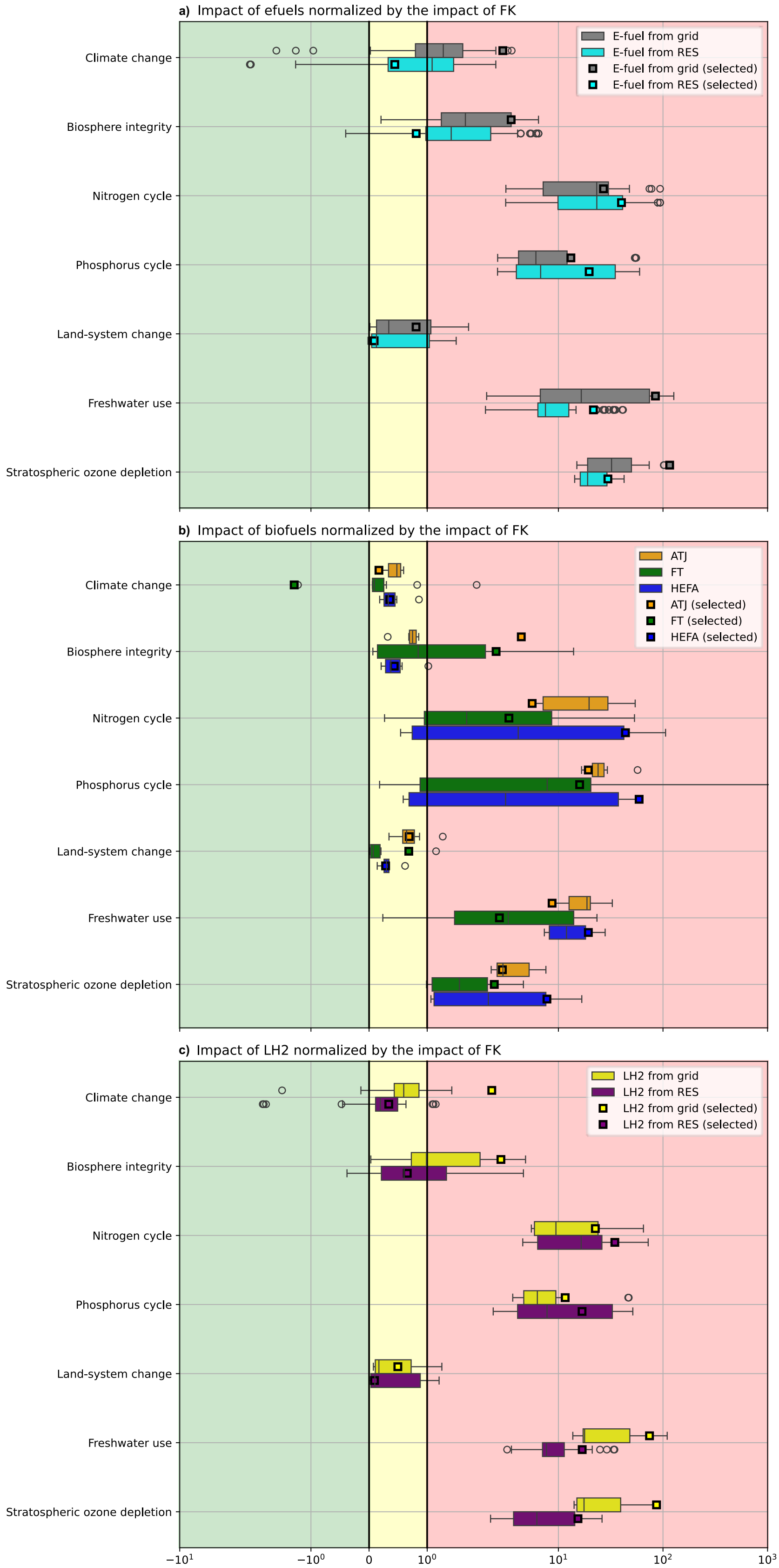

Supplementary Figure 2 - Impacts of AECs relative to impacts of fossil kerosene

The standard PB-LCIA methodology is used to assess impacts of burning 1 MJ of the AECs corresponding to the 123 LCIs analyzed under current technosphere. Impacts are then normalized by the impacts of burning 1 MJ of fossil kerosene to evaluate the improvement or degradation of the AEC with respect to fossil kerosene (Supplementary Figure 2). For each PB, the impact relative to the impact of fossil kerosene is reported for the 123 AECs considered in this work. Points in the right-hand zone (resp. middle zone) indicate that the AEC increases (resp. decreases) the impact relative to fossil kerosene. Points in the left-hand zone indicate that the AEC has a negative impact on the PB considered. The

climate-based selection of AECs is represented by a black square. **a.** Efuels: efuels produced with grid electricity are represented in gray and with RES electricity in blue. **b.** Biofuels: AtJ are represented in orange, FT in green and HEFA in blue. **c.** Liquid hydrogen: LH_2 produced with grid electricity are represented in yellow and with RES electricity in purple. In the box-plots, the centre line represents the median, box limits the 25th and 75th percentiles, whiskers extend to 1.5 times the interquartile range, and individual points beyond the whiskers are shown as outliers.

Supplementary Table 4 - Ranking of biofuel environmental performances by pathway.

The impact of each biofuel is assessed using the PB-LCIA methodology[25]. CC=climate change, BI= biosphere integrity, N=nitrogen cycle, P=phosphorus cycle, FWU=freshwater-use, SOD=stratospheric ozone depletion.

Reference	Feedstock	Rank CC	Rank BI	Rank N	Rank P	Rank FWU	Rank SOD		Sum of ranks
**Alcohol-to-Jet**									
Björnsson et al.[13]	Sawdust	**1**	7	1	2	1	3		**15**
Capaz et al.[14]	Sugarcane residues	3	4	2	4	6	1		20
Capaz et al.[14]	Forestry residues	4	5	3	3	5	2		22
Bedin G.[15]	Forestry residues	2	1	4	6	4	4		21
Wang et al.[16]	Corn cob	6	3	5	1	2	5		22
Wang et al.[16]	Corn cob	5	2	6	5	3	6		27
Wang et al.[16]	Corn cob	7	6	7	7	7	7		41
**Fischer-Tropsch**									
Almena et al.[17]	Pinewood	**1**	8	7	7	5	8		36
Björnsson et al.[13]	Logging residues	10	9	8	5	10	10		52
Capaz et al.[14]	Sugarcane residues	9	6	3	8	7	7		40
Capaz et al.[14]	Forestry residues	4	2	1	2	1	3		**13**
Cavalett et Cherubini[18]	Forestry residues	6	3	2	1	2	5		19
Cavalett et Cherubini[18]	Forest products	7	7	6	6	3	6		35
Rojas Michaga et al.[19]	Forestry residues	2	10	9	9	8	4		43
Lai et al.[20]	Forestry residues	3	1	5	4	4	2		19
Lai et al.[20]	Black liquor	8	4	4	3	6	1		26
Real Guimaraes et al.[21]	Sugarcane bagasse	5	5	10	10	8	9		47
**Hydroprocessed Esters and Fatty Acids**									
Barbera et al.[22]	Used cooking oil	3	3	2	1	1	2		12
Capaz et al.[14]	Beef tallow	1	1	1	2	2	1		8
Capaz et al.[14]	Used cooking oil	2	2	3	3	3	3		16
Liu et al.[23]	Jatropha	5	5	4	4	4	4		**26**
Liu et al.[23]	Castor	6	6	6	5	6	6		35
Mousavi-Avval et al.[24]	Pennycress	**4**	4	5	6	5	5		29

Supplementary Table 5 - Biofuel selection used in the main analysis (climate-based) and in the sensitivity analysis (PB-based).

For each biofuel pathway (AtJ, Alcohol-to-Jet; FT, Fischer-Tropsch; HEFA, Hydroprocessed Esters and Fatty Acids), the table shows the specific feedstock and life-cycle inventory retained under two selection criteria: (i) climate-based selection, which retains the biofuel with the lowest impact on climate change; and (ii) PB-based selection, which retains the biofuel with the lowest sum of ranks across the six planetary boundaries (used for the sensitivity analysis shown in Supplementary Figures 8, 14 and 15). CCS: carbon capture and storage. For HEFA, used cooking oil and beef tallow have the best climate performance, but they would be redundant with sawdust which is also a residue, and their availability is severely limited[26,27]. For this reason, the choice sawdust/pinewood+CCS/pennycress is more representative as would be the choice sawdust/pinewood+CCS/used cooking oil which would be entirely based on climate performance. The same arguments explain the PB-based selection.

	**AtJ**	**FT**	**HEFA**
**Climate-based**	Sawdust[13]	Pinewood + CCS[17]	Pennycress[24]
**PB-based**	Sawdust[13]	Forestry residues[14]	Jatropha[23]

Supplementary Note 2 - Dynamic climate change impact assessment

The calculation of the additional concentration of greenhouse gases (GHGs) in the atmosphere follows the approach of Ryberg et al.[25] based on the Bern Simple Climate Model (SCM):

$$C_{\mathrm{GHG}}(t)=m_{\mathrm{GHG}}(t)*\frac{(10^6\ \mathrm{ppmv})/m_{\mathrm{air}}}{M_{\mathrm{GHG}}/M_{\mathrm{air}}} \quad (1)$$

with $m_{\mathrm{GHG}}(t)$ the additional mass of GHG in the atmosphere in year t, m_{air} the mass of the atmosphere (5.15E+18 kg)[28], M_{GHG} the molar mass of the GHG and M_{air} the molar mass of air.

The additional mass of GHGs in the atmosphere is given as supplementary equation (2):

$$m_{\mathrm{GHG}}(t)=\sum_{t_n=0}^{t} E_{\mathrm{GHG}}(t_n)\times fr_{\mathrm{GHG}}(t-t_n) \quad (2)$$

where $E_{\mathrm{GHG}}(t_n)$ represents the emission of GHGs in year t_n (with t_n ranging from the beginning to the end of the time span of our scenario) and $fr_{\mathrm{GHG}}(t-t_n)$ denoting the fraction of GHGs emitted in year t_n remaining in the atmosphere in year t.

$fr_{\mathrm{GHG}}(t)$ is estimated using the methods of Shine et al.[29] for methane and nitrous oxide and Joos et al.[30] for CO_2:

$$fr_{\mathrm{GHG}}(t) = \exp^{\left(-t/\alpha_{\mathrm{GHG}}\right)} \quad (3)$$

$$fr_{\mathrm{CO_2}}(t) = a_0 + \sum_{i=1}^{4} a_i * \exp^{\left(-t/\beta_i\right)} \quad (4)$$

where α_{GHG} represents the lifetime of the GHG (12.4 years for methane and 121 years for nitrous oxide) in the atmosphere and the factors a_0, a_i and β_i denote the specific coefficients and time constants of the Bern SCM (a_0=0.212, a_1=0.244, a_2=0.336, a_3=0.207, β_1=336.4 years, β_2=27.89 years, and β_3=4.055 years). The evolution of radiative forcing is calculated as supplementary equation (5)[31]:

$$\Delta\mathrm{RF}_{\mathrm{CO_2}}(t) = \gamma_{\mathrm{CO_2}} \times \ln\left(C_{\mathrm{CO_2}}(t)/C_{\mathrm{CO_2}}(t_0)\right) \quad (5)$$

$$\Delta\mathrm{RF}_{\mathrm{CH_4}}(t) = \gamma_{\mathrm{CH_4}} \times \left(\sqrt{C_{\mathrm{CH_4}}(t)} - \sqrt{C_{\mathrm{CH_4}}(t_0)}\right) - \left(f\left(C_{\mathrm{CH_4}}(t), C_{\mathrm{N_2O}}(t_0)\right) - f\left(C_{\mathrm{CH_4}}(t_0), C_{\mathrm{N_2O}}(t_0)\right)\right) \quad (6)$$

$$\Delta RF_{\mathrm{N_2O}}(t) = \gamma_{\mathrm{N_2O}} \times \left(\sqrt{C_{\mathrm{N_2O}}(t)} - \sqrt{C_{\mathrm{N_2O}}(t_0)}\right) - \left(f\left(C_{\mathrm{CH_4}}(t_0), C_{\mathrm{N_2O}}(t)\right) - f\left(C_{\mathrm{CH_4}}(t_0), C_{\mathrm{N_2O}}(t_0)\right)\right) \quad (7)$$

where

$$f(M,N) = 0.47 * \ln[1 + 2.01 * 10^{-5} * \ (MN)^{0.75} + 5.31 * 10^{-15} * M * (MN)^{1.52}] \quad (8)$$

with $\Delta \mathrm{RF_{GHG}}(t)$ the radiative forcing induced by GHG emissions from t_0 to t, γ_{GHG} a constant specific to GHGs (5.35 for CO_2, 0.036 for CH_4, and 0.12 for N_2O) and $C_{\mathrm{CO_2}}(t_0)$ (ppm), $C_{\mathrm{CH_4}}(t_0)$ (ppb), and $C_{\mathrm{N_2O}}(t_0)$ (ppb) the atmospheric concentrations of GHGs at the beginning of the scenario under study (410 ppm, 1866 ppb, and 332 ppb, respectively, for 2019)[32].

These equations allow us to calculate the evolution of the CO_2 concentration in the atmosphere and the radiative forcing induced by aviation since the beginning of the scenario under consideration. To obtain the total impact of aviation, we need to add its historical impact. For this purpose, data from Lee et al.[33] are used.

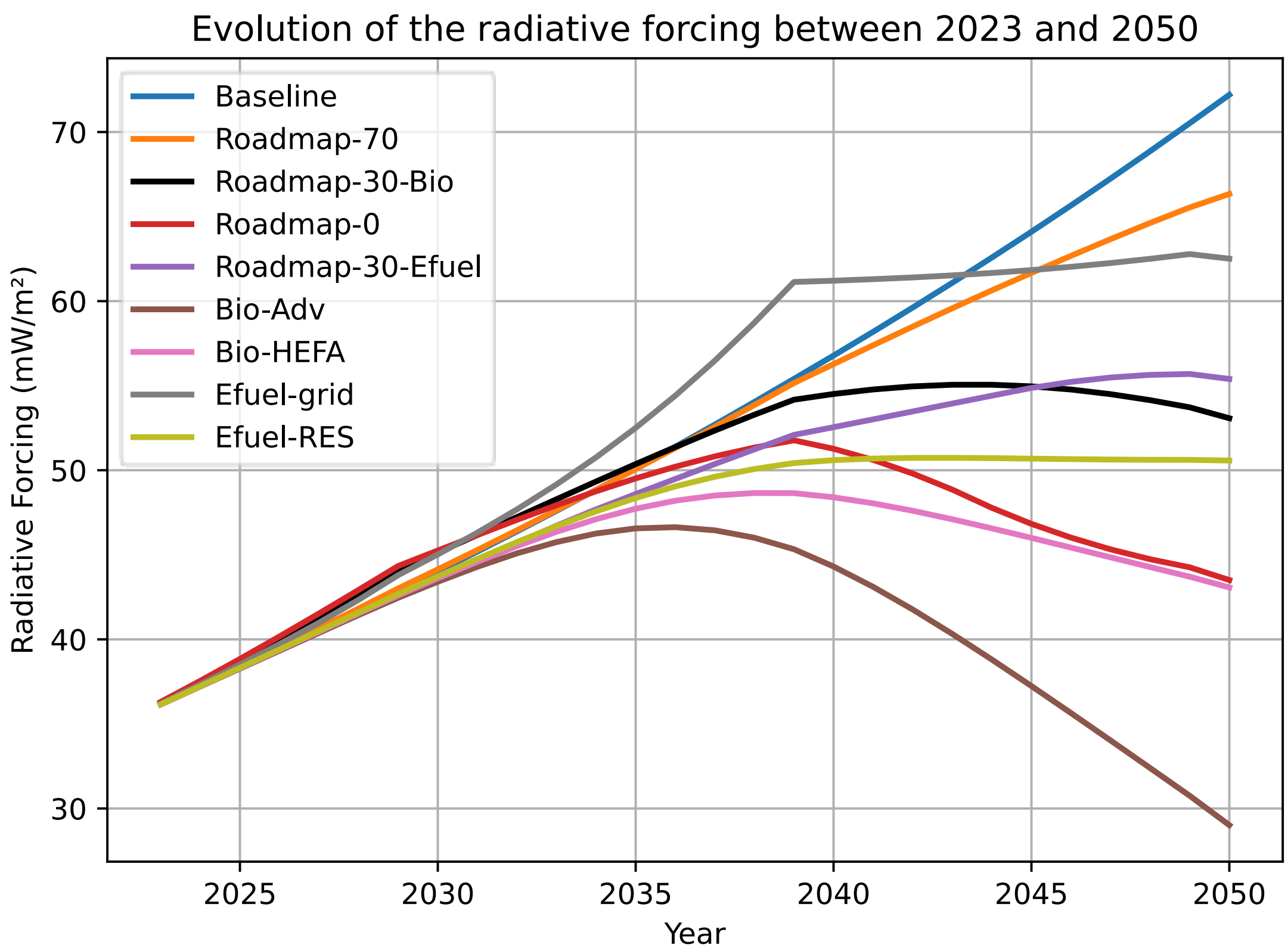

Supplementary Figure 3 - Evolution of radiative forcing between 2023 and 2050.
This figure represents the evolution of the impact of aviation on the climate change planetary boundary over time (2023-2050) for each scenario using REMIND SSP2-RCP2.6.

Supplementary Note 3 - Dynamic biosphere integrity impact assessment
Biosphere integrity is the other core PB, affected mainly by five key drivers: land-use and sea-use, direct exploitation, climate change, pollution, and invasive non-native species[34].

The impact on biosphere integrity is assessed through the biosphere intactness index (BII). Galan-Martin et al.[35] developed a methodology to assess the impact on BII accounting for two drivers: direct land use and the increase in average temperature. The total impact on BII is the sum of impacts owing to direct land use and to climate change:

$$\mathrm{BII}_{\mathrm{loss}_{\%,\,\mathrm{total}}}(t) = \mathrm{BII}_{\mathrm{loss}_{\%,\,\mathrm{DLU}}}(t) + \mathrm{BII}_{\mathrm{loss}_{\%,\,\mathrm{CC}}}(t) \quad (9)$$

where $\mathrm{BII}_{\mathrm{loss}_{\%,\,\mathrm{total}}}(t)$ is the total BII loss (%) at year t, $\mathrm{BII}_{\mathrm{loss}_{\%,\,\mathrm{DLU}}}(t)$ the BII loss (%) due to direct land-use at year t and $\mathrm{BII}_{\mathrm{loss}_{\%,\,\mathrm{CC}}}(t)$ the BII loss (%) due to climate change at year t.

The biosphere integrity impact due to direct land-use is given as supplementary equation (10):

$$\mathrm{BII}_{\mathrm{loss}_{\%,\,\mathrm{DLU}}}(t) = \sum_{i} Occupation_i(t) * \mathrm{CF}_{\mathrm{BII}_{\mathrm{loss}_{\mathrm{DLU},i}}} \tag{10}$$

where $\mathrm{Occupation}_i(t)$ is the annual occupation of surface of type *i* by aviation at year *t* (m^2 yr^1) and $\mathrm{CF}_{\mathrm{BII}_{\mathrm{loss}_{\mathrm{DLU},i}}}$ the characterization factor developed by Galan-Martin et al.[35] expressing the percentage of BII loss per square-meter-year of type *i* occupied (%BII loss m^{-2} yr^{-1}).

The characterization factors related to the climate change driver of BII loss developed by Galan-Martin et al.[35] are not suitable for similar reasons as the climate change characterization factor of PB-LCIA, namely, they are computed assuming a constant emission of 1 kg of GHG over 100 years. Galan-Martin et al.[35] propose the characterization factor given as supplementary equation (11):

$$\mathrm{CF}_{\mathrm{BII,\,CC}} = \mathrm{TR}_{\mathrm{GES,\,100}} * F_{\mathrm{MSA\ loss\ °C^{-1}}} \tag{11}$$

with $\mathrm{TR}_{\mathrm{GES,\,100}}$ representing the temperature increase associated with a constant emission of 1 kg of GHG over 100 years and $F_{\mathrm{MSA\ loss\ °C^{-1}}}$ denoting the loss of mean species abundance (considered as a good proxy for BII, see Hanafiah et al.[36]) per degree of temperature increase[36]. To account for the time-dependency, we consider the impact assessment method given as supplementary equation (12):

$$\mathrm{BII}_{\mathrm{loss}_{\%,\,\mathrm{CC}}}(t) = 100 * \left(\lambda * \mathrm{RF}_{\mathrm{aviation}}(t) * F_{\mathrm{MSA\ loss\ °C^{-1}}}\right) \tag{12}$$

where λ (=1.06 $K(Wm^{-2})^{-1}$)[37,38] is a climate sensitivity parameter indicating the change in surface temperature per unit of additional radiative forcing and $\mathrm{RF}_{\mathrm{aviation}}(t)$ is the radiative forcing induced by aviation, as calculated previously for the climate change PB.

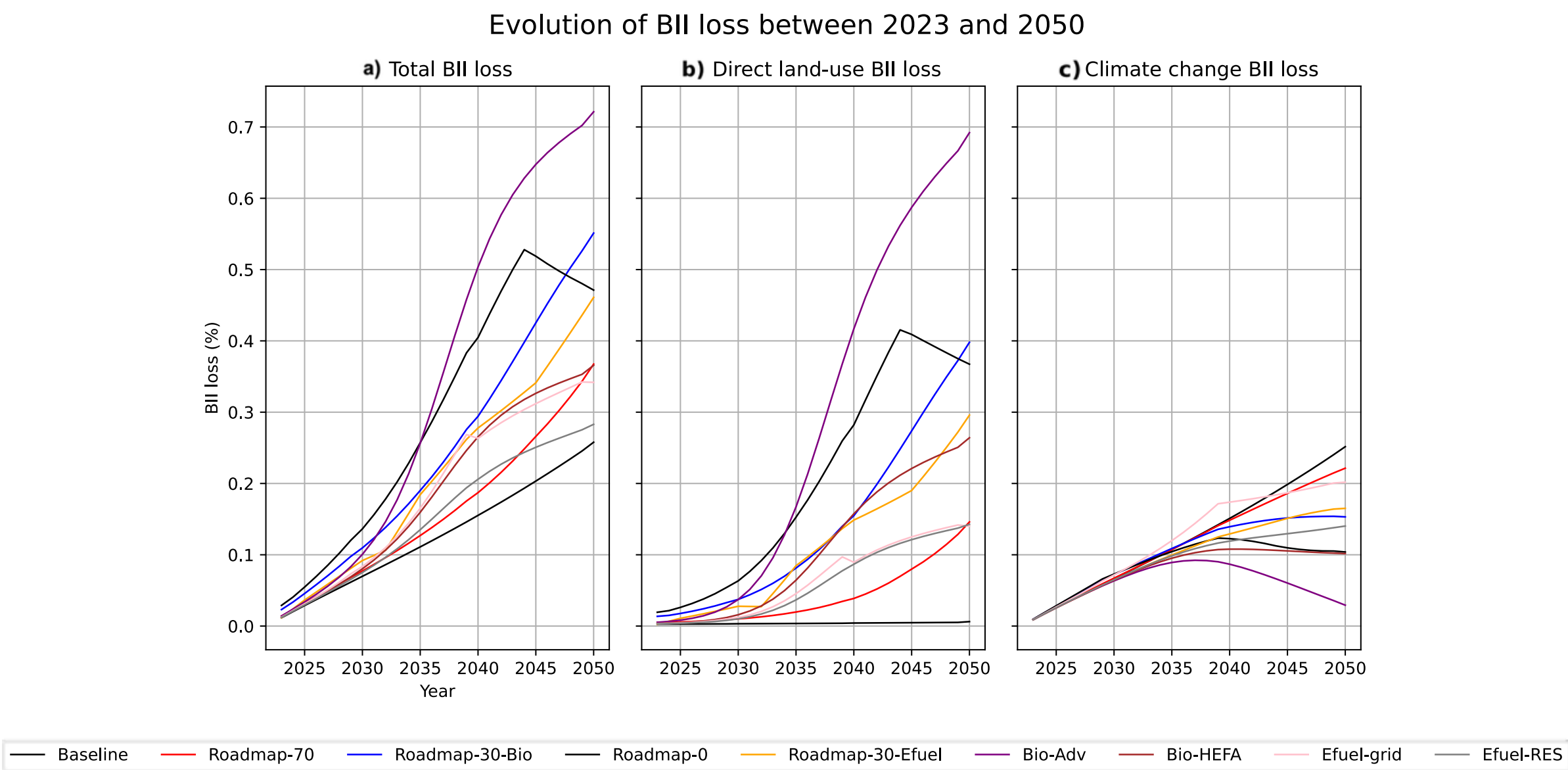

Supplementary Figure 4 - Evolution of biosphere integrity impact between 2023 and 2050. These three panels illustrate the evolution of aviation's impact on the biosphere integrity planetary boundary under the REMIND SSP2-RCP2.6 scenario. Results are disaggregated into two components: **b.** direct land use and **c.** climate change. **a.** presents the total impact on biosphere integrity as the linear sum of these two components.

Supplementary Note 4 - Precision on land-related metrics

Two land-related metrics are considered in this study direct land-use and land occupation.

Direct land-use

This metric is used to assess the impact of land occupation on the biosphere integrity PB. In LCIs it includes only elementary flows starting with *"Occupation…"*. The corresponding characterization factors[35,36] convert land occupation (in square meter-years) into a percentage loss of the Biodiversity Intactness Index, on which the control variable for the biosphere integrity PB is computed. This is a non-cumulative metric.

Land occupation

This is a resource-use metric. In the context of our study, it represents the land area occupied annually (non-cumulative metric) for two purposes: (i) electricity production, calculated by multiplying the electricity generated by the power density of the production pathway; and (ii) biomass cultivation, estimated by multiplying the biomass requirements for biofuel production by the land-use intensity of the corresponding feedstock.

Supplementary Note 5 - Prospective Technosphere

REMIND SSP2-RCP2.6

The Shared Socioeconomic Pathway (SSP) 2, often described as the "middle of the road" scenario, assumes global development trends that largely follow historical patterns, presenting moderate challenges for both mitigation and adaptation. When combined with the Representative Concentration Pathway (RCP) 2.6 (a stringent climate mitigation scenario aimed at limiting global warming to below 2°C by 2100 through rapid decarbonization and strong climate policies) it aligns with the objectives of the Paris Agreement.

REMIND (Regional Model of Investment and Development) is an Integrated Assessment Model (IAM) developed by the Potsdam Institute for Climate Impact Research (PIK).
Supplementary Figure 5 illustrates the evolution of electricity production (final energy), biomass production (primary energy), energy crops area, freshwater withdrawal for electricity and DACCS for REMIND SSP2-RCP2.6 in blue. The evolution of electricity mix is displayed in Supplementary Figure 6-a.

IMAGE SSP2-RCP2.6

IMAGE is an Integrated Assessment Model (IAM) developed by Utrecht University (UU) and the PBL Netherlands Environmental Agency.
Supplementary Figure 5 illustrates the evolution of electricity production (final energy), biomass production (primary energy), energy crops area and freshwater withdrawal for electricity for IMAGE SSP2-RCP2.6 in black (blue for freshwater). The evolution of electricity mix is displayed in Supplementary Figure 6-b.

It should be noted that no DACCS is considered in IMAGE SSP2-RCP2.6.

REMIND SSP1-RCP1.9

SSP1, also referred to as the "Sustainability" or "Green Growth" scenario, envisions a world transitioning toward sustainable development, characterized by strong international cooperation, rapid technological advancements, and low population growth. RCP 1.9, one of the most ambitious climate mitigation pathways, aims for net-zero CO_2 emissions by around 2050 and seeks to limit global warming to below 1.5°C by 2100, aligning with the Paris Agreement.

Supplementary Figure 5 illustrates the evolution of electricity production (final energy), biomass production (primary energy), energy crops area, freshwater withdrawal for electricity and DACCS for REMIND SSP1-RCP1.9 in orange. The evolution of electricity mix is displayed in Supplementary Figure 6-c.

REMIND SSP2-NPi

NPi (National Policies Implemented) represents currently enacted national climate and energy policies without incorporating additional future mitigation measures. REMIND SSP2-NPi models a world where existing policies influence emission trajectories but fall short of achieving stringent climate targets, resulting in continued global warming.

Supplementary Figure 5 illustrates the evolution of electricity production (final energy), biomass production (primary energy), energy crops area and freshwater withdrawal for electricity for REMIND SSP2-NPi in red (blue for freshwater). The evolution of electricity production is displayed in Supplementary Figure 6-d. It should be noted that no DACCS is considered in REMIND SSP2-NPi.

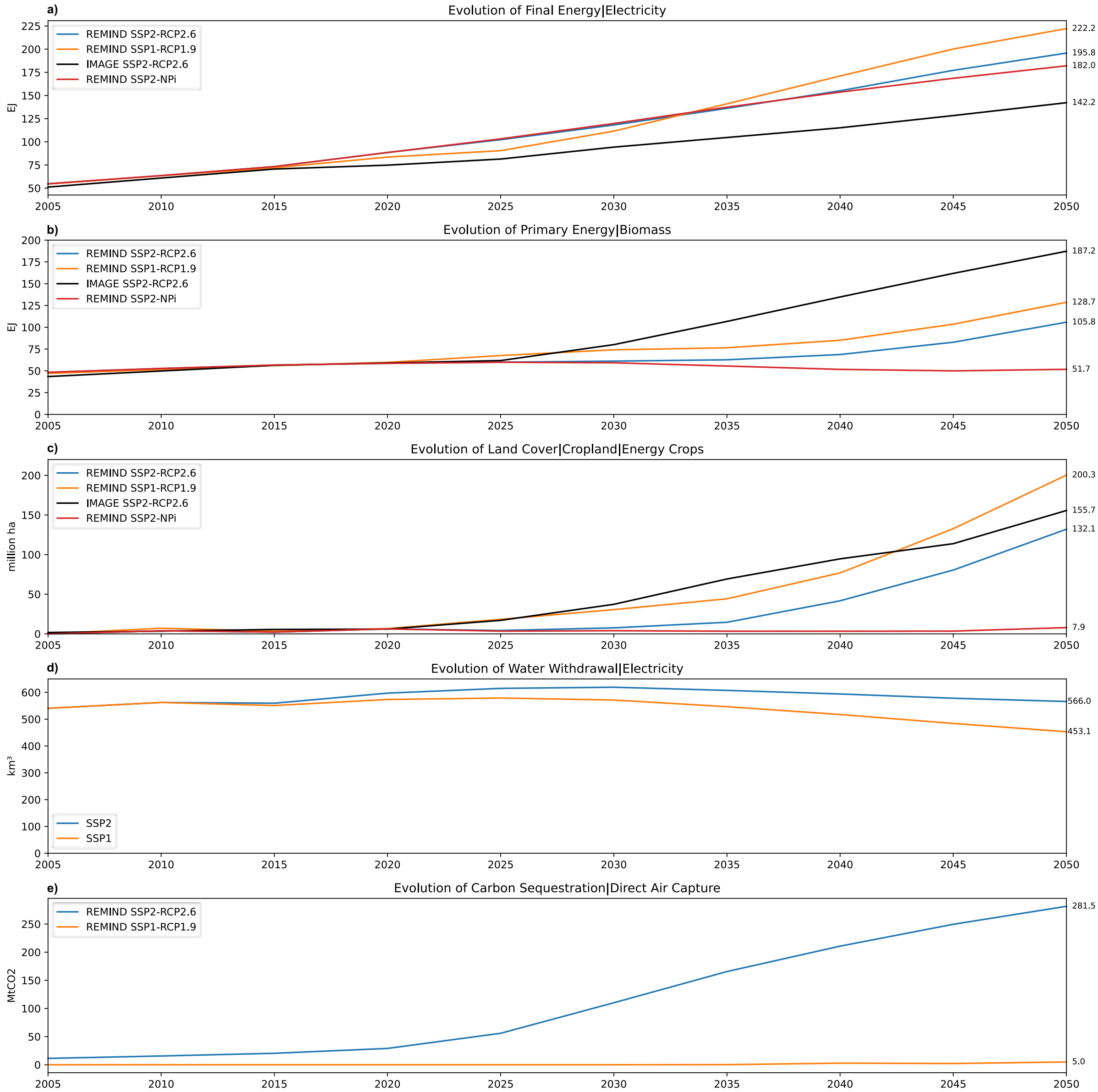

Supplementary Figure 5 - Evolution of resources availability in the considered IAMs.

Panels show the evolution of **a.** electricity; **b.** biomass; **c.** energy crops; **d.** freshwater withdrawal; **e.** direct air capture. Data on electricity, biomass, energy crops and Direct Air Carbon Capture and Storage (DACCS) were sourced from the AR6 Scenario Explorer hosted by IIASA[39]. Freshwater withdrawal data were obtained from Graham et al. [40]. The values used to normalize resource requirements (that is, the total global amount produced or used in the corresponding IAM scenario) are indicated to the right of each figure. As the exact combination of IAM version, SSP, and RCP used in premise is not publicly available, we assumed consistency with the closest available scenarios in the AR6 Scenario Explorer. Specifically, resource requirements modeled with REMIND SSP2-RCP2.6 in premise were normalized using values from REMIND-MAgPIE 2.1-4.3 DeepElec_SSP2_def_Budg1300; those modeled with REMIND SSP1-RCP1.9 were normalized using REMIND-MAgPIE 2.1-4.2 SusDev_SSP1-PkBudg900; those from REMIND SSP2-NPi were normalized using REMIND 2.1 R2p1_SSP2_NPi; and results from IMAGE SSP2-RCP2.6 were normalized using IMAGE 3.2 SSP2_SPA2_26I_D.

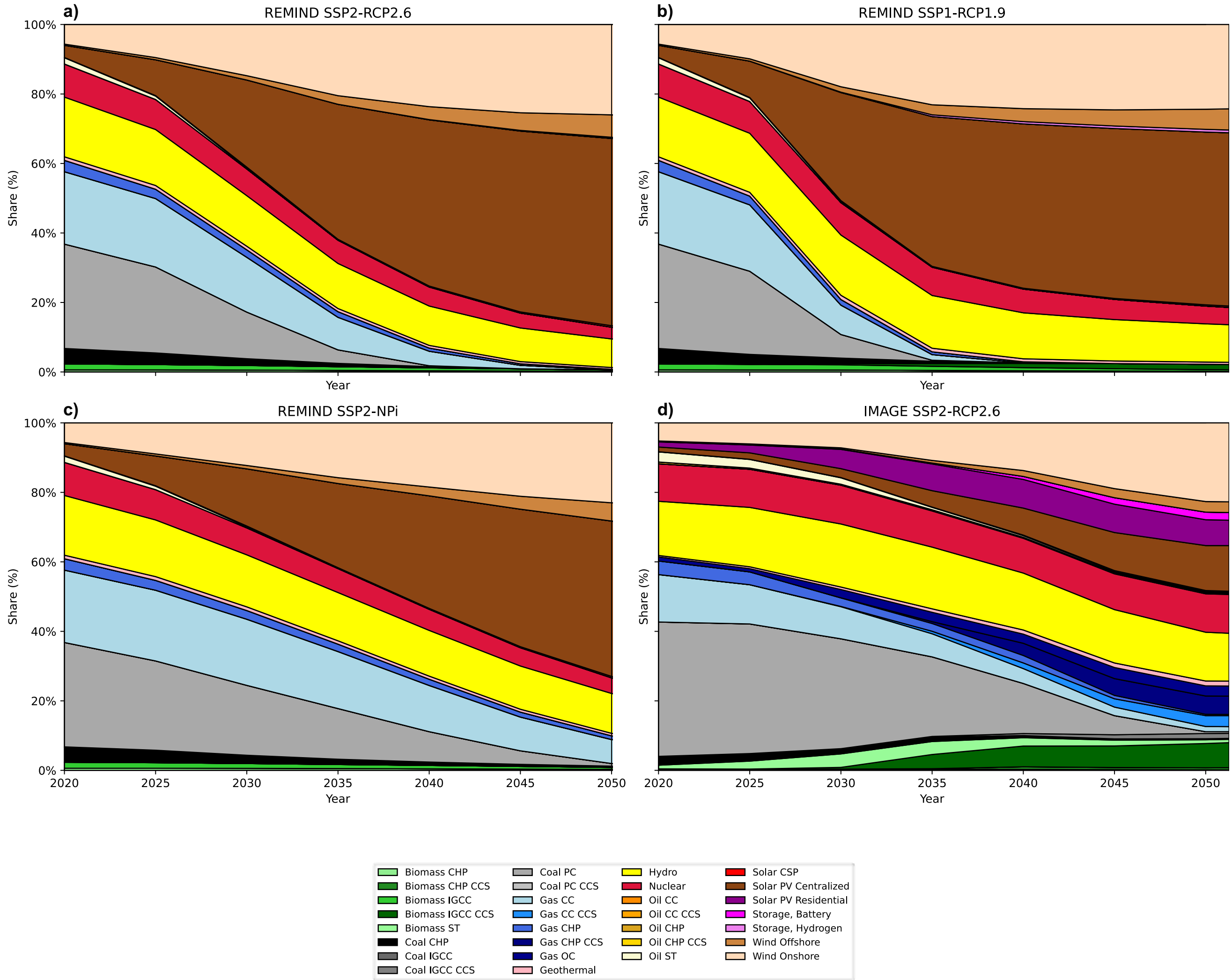

Supplementary Figure 6 - Evolution of electricity production in the considered Integrated Assement Models (IAMs).

Panels show the electricity mix evolution under **a.** REMIND SSP2-RCP2.6; **b.** REMIND SSP1-RCP1.9; **c.** REMIND SSP2-NPi; **d.** IMAGE SSP2-RCP2.6. Data are taken from the *premise* dashboard (v2.2.0)[41].

Supplementary Note 6 - Comparison of aviation traffic assumptions with IAM scenarios

In order to evaluate the consistency between the aviation demand trajectories assumed in this study and those implied by IAMs, we compared our traffic growth rates with aviation traffic projections from the AR6 Scenario Explorer[39]. Supplementary Table 7 summarizes the corresponding aviation traffic growth rates and the implied differences in traffic and energy demand over the period 2023–2050. All traffic and energy indicators are expressed relative to 2023 = 1.

In IMAGE SSP2-2.6 (named SSP2_SP12_26I_D) and SSP2-baseline, aviation traffic grows at average rates of 3.32% yr^{-1} and 3.59% yr^{-1}, respectively, compared to 3.7% yr^{-1} assumed in this study based on industry roadmaps. In contrast, in the stringent mitigation pathway IMAGE SSP1-1.9 (named SSP1_SPA1_19U_D), aviation traffic growth is substantially lower, at 1.94% yr^{-1}.

As shown in Supplementary Table 7, although SSP2 growth rates are close in annual terms, compounding over 2023–2050 leads to non-negligible differences in end-point traffic levels. Relative to identical traffic in 2023, the assumed growth rate of 3.7% yr^{-1} results in a traffic level in 2050 about 10% higher than in SSP2-2.6 and about 3% higher than in SSP2-baseline, while it is

about 59% higher than in SSP1-1.9. Assuming identical efficiency gains of 1.5% yr^{-1} in all scenarios, these differences translate directly into differences in aviation energy demand.

The column "Cumulative energy 2023–2050" in Supplementary Table 7 reports the time-integrated aviation energy demand over the period 2023–2050, expressed in units of the annual demand in 2023 (2023 = 1). It therefore represents the total energy consumed over the transition period relative to the 2023 reference year and captures the cumulative pressure on energy resources induced by different aviation demand trajectories. The cumulative energy demand modelled in this study is 5.5% higher than in SSP2-2.6, 1.6% higher than in SSP2-baseline, and 28% higher than in SSP1-1.9.

Supplementary Table 6 – Comparison of current roadmaps and Integrated Assessment Models (IAMs) assumptions.

For each roadmap and IAM scenario considered, the table indicates traffic growth rates and cumulative energy demand over 2023-2050, assuming identical efficiency gains of 1.5% yr^{-1} in all scenarios.

Scenario	Traffic growth rate (% yr^{-1})	Traffic in 2050 relative to 2023	Global aviation fuel demand in 2050 relative to 2023*	Cumulative fuel demand 2023-2050 relative to 2023*	Source
This study	3.7	2.67	1.78	37.96	-
IMAGE SSP2_2.6	3.32	2.42	1.62	35.97	Byers et al.[39]
IMAGE SSP2-baseline	3.59	2.59	1.73	37.34	
IMAGE SSP1-1.9	1.94	1.68	1.12	29.70	

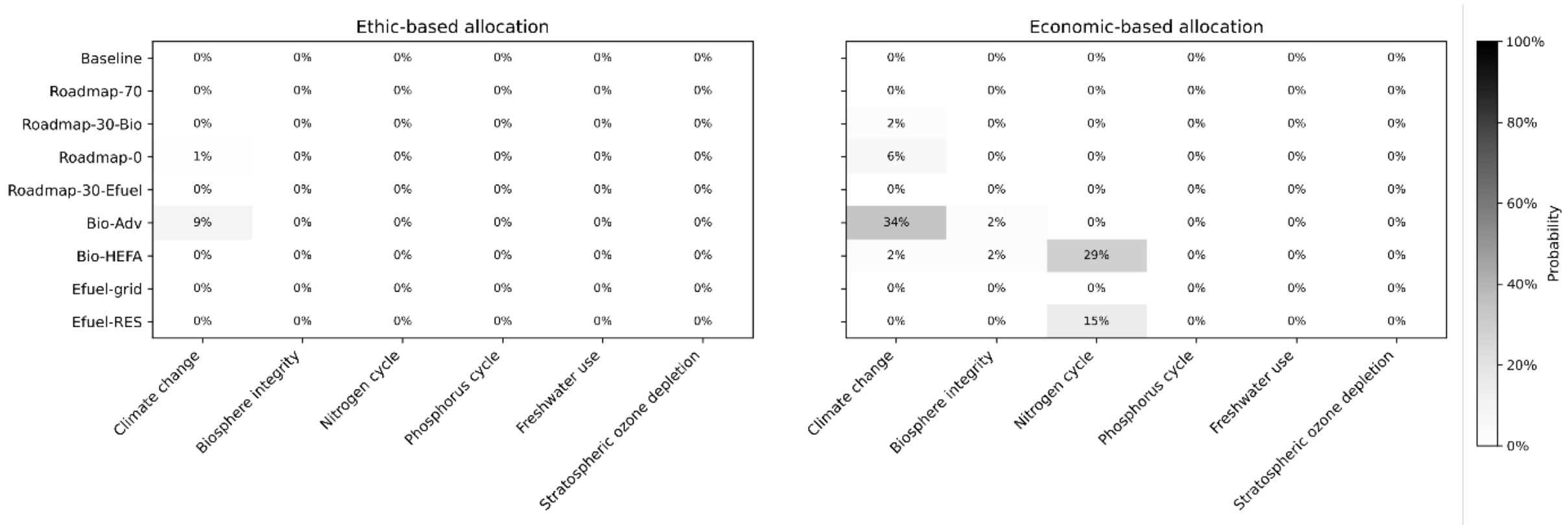

Supplementary Figure 7 – Monte Carlo uncertainty analysis of the AESA results.

Numbers indicate, for each aviation scenario and each planetary boundary (PB), the probability that uncertainty in background technosphere life-cycle inventories alters the transgression outcome (transgressed vs. non-transgressed), based on 1,000 Monte Carlo simulations, relative to the deterministic absolute environmental sustainability assessment (AESA) result for 2050 shown in Figure 3 of the main text. Uncertainty analysis is conducted with the same assumptions (biofuel selection, underlying storyline, traffic growth rate, energy efficiency gains and historical impacts) than in the main manuscript. Results are shown for the ethic-based allocation (left panel) and the economic-based allocation (right panel). For instance, the 9% in the Bio-Adv/Climate Change case means that out of 1,000 Monte-Carlo draws, only 9% led to a conclusion different than in the main article. Overall, most values are close or equal to 0%, meaning that uncertainty in the background technosphere life-cycle inventories does not affect the qualitative conclusion in the main text. Only three cases exceed 10%: Bio-Adv/Climate Change (34%), Bio-HEFA/Nitrogen (29%) and Efuel-RES/Nitrogen (15%).

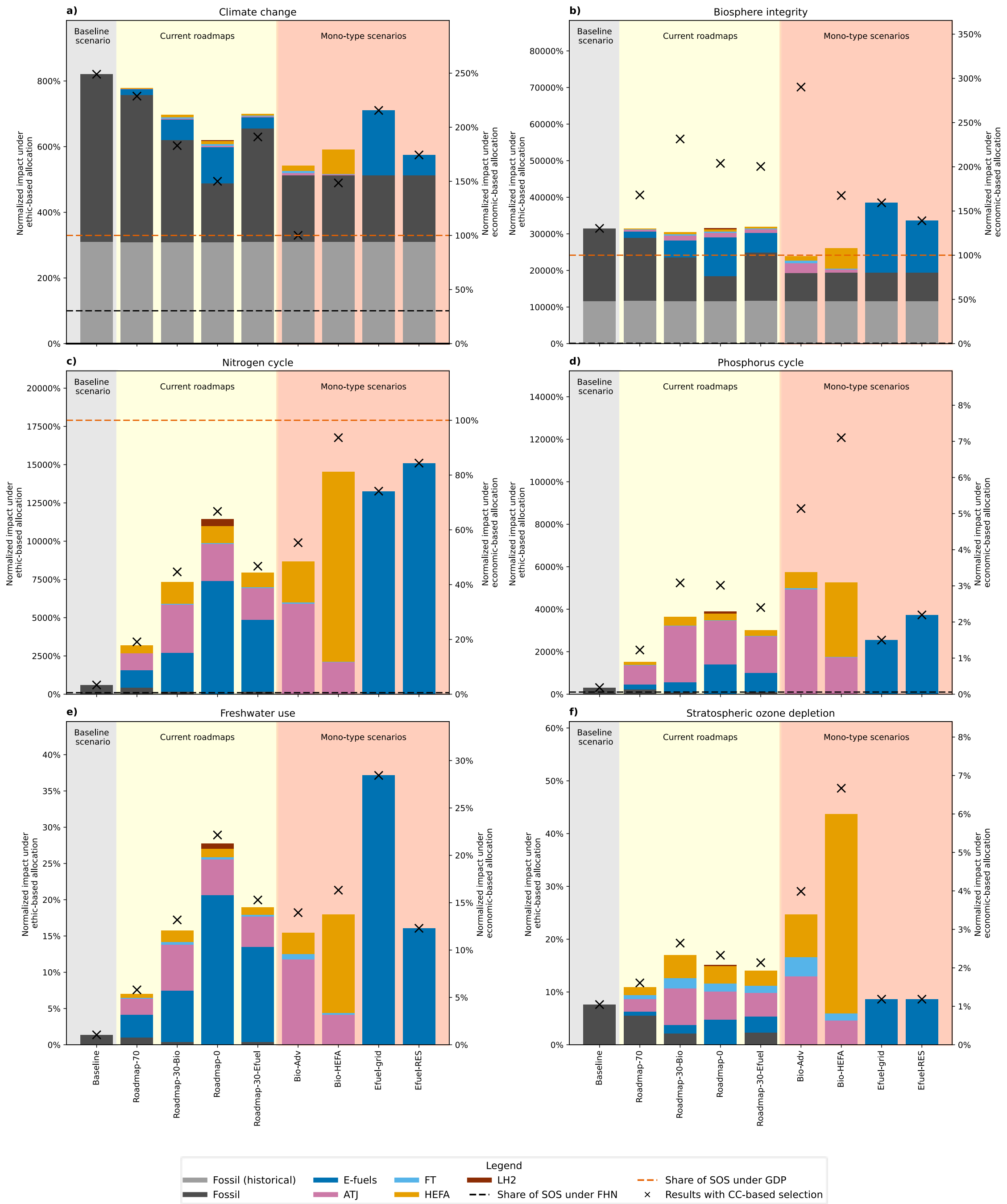

Supplementary Figure 8 - Sensitivity analysis of AESA to the biofuel selection.

Sensitivity of the results of Figure 3 in the main text is assessed for a different biofuel selection. Here the same analysis as in Figure 3 is carried out but using the PB-based biofuel selection instead of the climate-based selection. Black crosses represent results with the climate-based biofuel selection. Panels show results for: **a**. Climate change. **b**. Biosphere integrity. **c**. Nitrogen cycle. **d**. Phosphorus cycle. **e**. Freshwater use. **f**. Stratospheric ozone depletion.

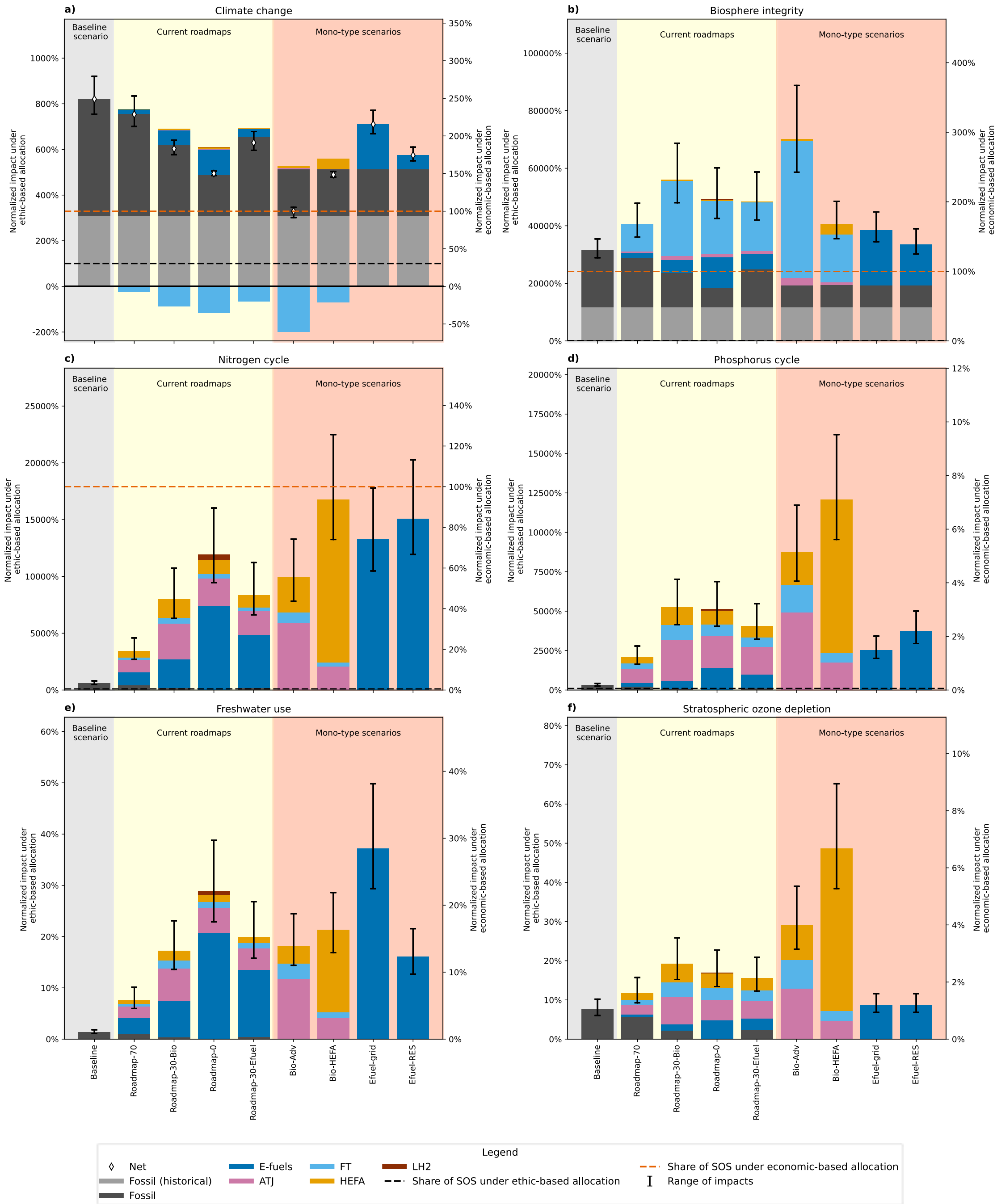

Supplementary Figure 9 - Sensitivity analysis of AESA to traffic growth rates and efficiency gains.

Sensitivity of the results of Figure 3 in the main text is assessed when the annual traffic growth rate (set at 3.7% yr^{-1} in the main text) and efficiency gains (set at 1.5% yr^{-1} in the main text) are varied. Traffic is varied between 2.8% yr^{-1} and 4.2% yr^{-1} and efficiency gains between 0.9% yr^{-1} and 1.5% yr^{-1}. Intervals represent the resulting variation of the impact. Panels show results for: **a**. Climate change. **b**. Biosphere integrity. **c**. Nitrogen cycle. **d**. Phosphorus cycle. **e**. Freshwater use. **f**. Stratospheric ozone depletion.

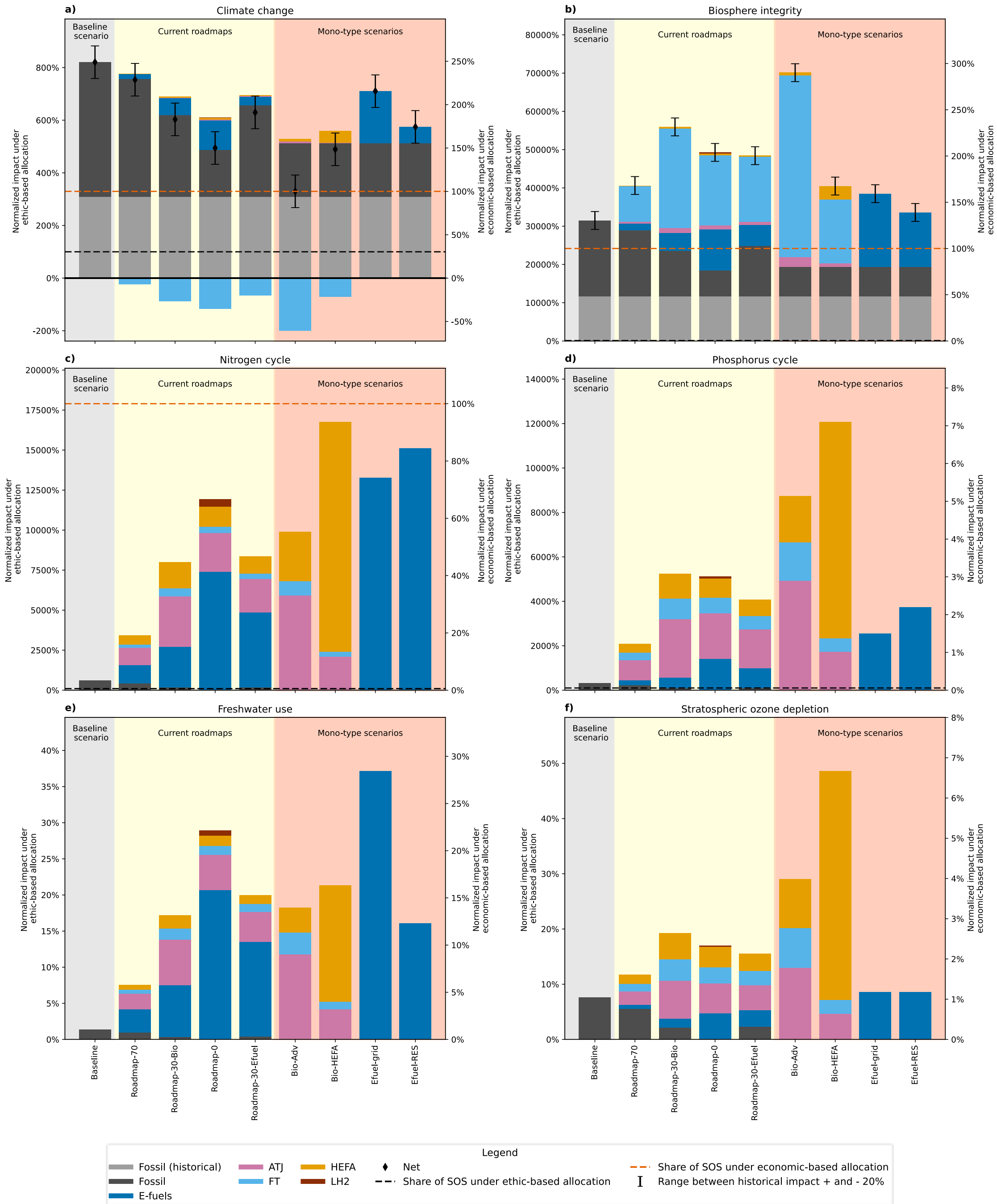

Supplementary Figure 10 - Sensitivity analysis of AESA to historical impact uncertainties. Sensitivity of the results of Figure 3 in the main text is assessed with respect to uncertainties on historical emissions. Values of historical emissions used for Figure 3 are varied by +/-20% and intervals capture the resulting variations in impacts. Historical emissions only impact "stock" PBs (climate change and biosphere integrity) whose state depends on past emissions/impacts. Panels show results for: **a**. Climate change. **b**. Biosphere integrity. **c**. Nitrogen cycle. **d**. Phosphorus cycle. **e**. Freshwater use. **f**. Stratospheric ozone depletion.

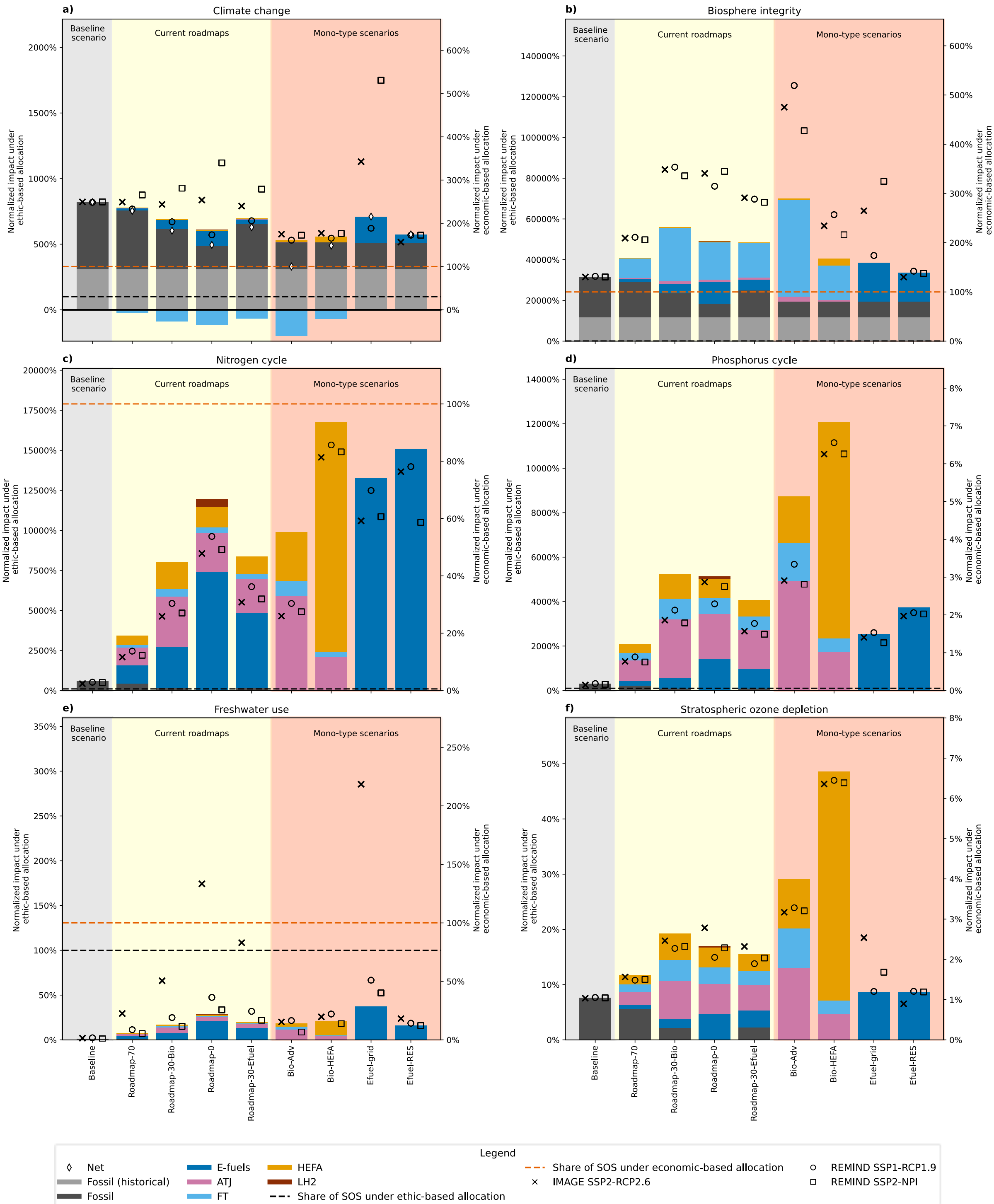

Supplementary Figure 11 - Sensitivity analysis of AESA to underlying development storyline.

Sensitivity of the results of Figure 3 in the main text is assessed with respect to the choice of the underlying story line. Panels show results for: **a**. Climate change. **b**. Biosphere integrity. **c**. Nitrogen cycle. **d**. Phosphorus cycle. **e**. Freshwater use. **f**. Stratospheric ozone depletion.

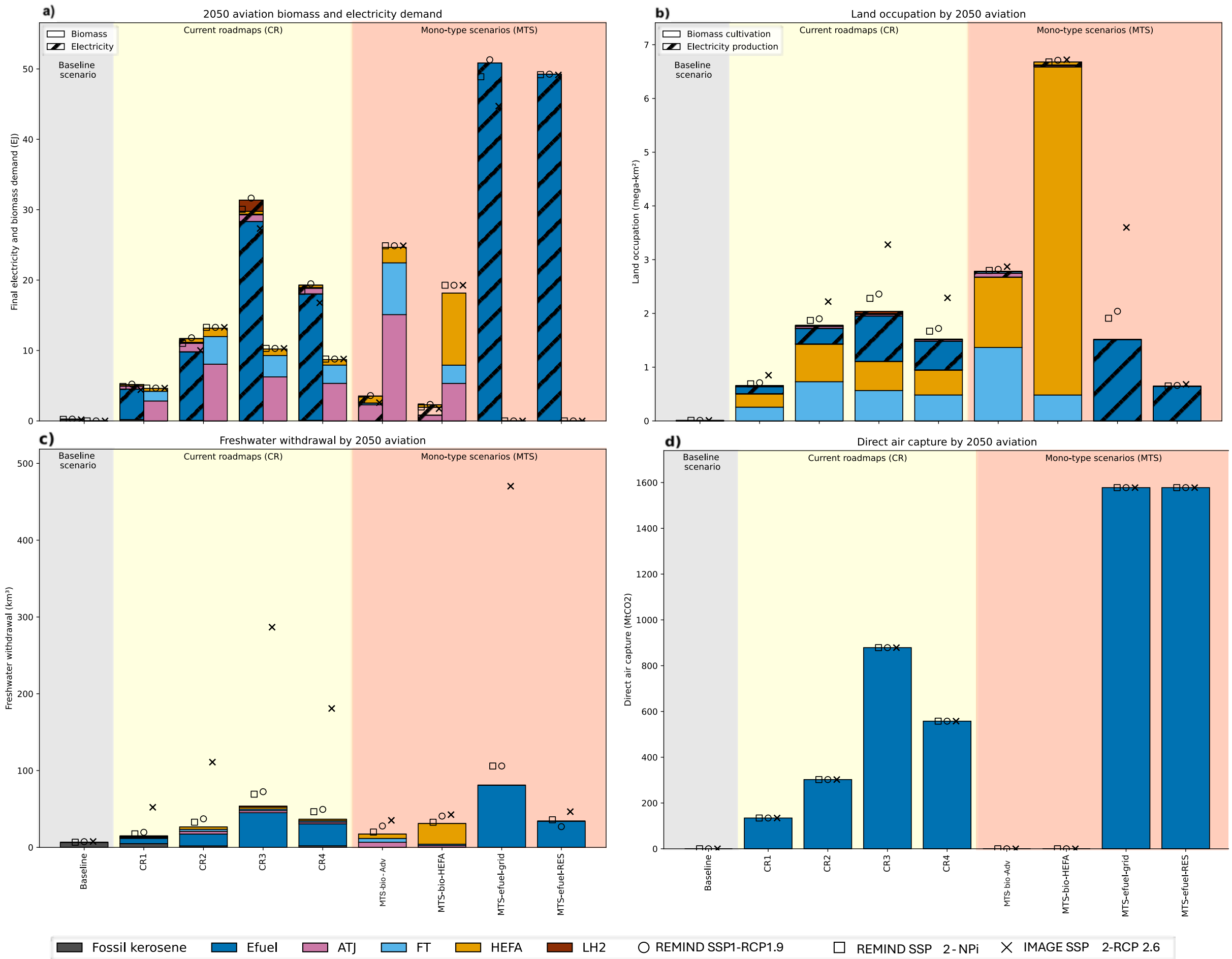

Supplementary Figure 12 - Sensitivity analysis of 2050 aviation absolute resource requirements to underlying development storyline.

The same analysis than in Figure 4 in the main text is performed for different underlying story lines. Because the absolute amount of resources differs in different storylines, only absolute values are displayed: relative results are provided in Supplementary Figure 13. Panels show results for **a.** biomass and electricity (in EJ); **b.** land occupation (in Mkm^2); **c.** freshwater withdrawal (in km^3); **d.** Direct Air Capture (DAC, in $MtCO_2$).

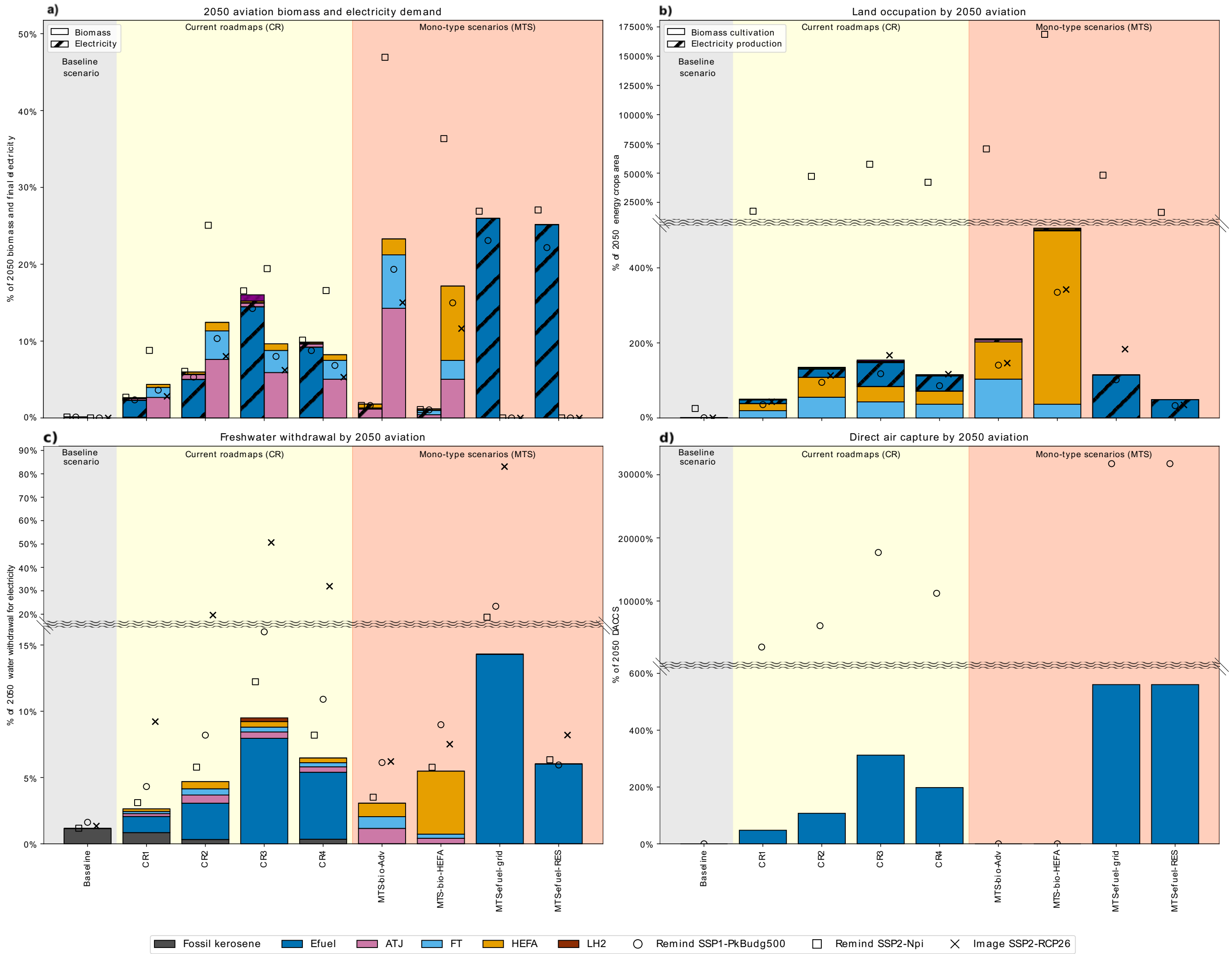

Supplementary Figure 13 - Sensitivity analysis of 2050 aviation relative resource requirements to underlying development storyline.

Same as Supplementary Figure 12, but relative resource requirements are displayed. Panels show results for **a.** biomass and electricity (in EJ); **b.** land occupation (in Mkm^2); **c.** freshwater withdrawal (in km^3); **d.** Direct Air Capture (DAC, in $MtCO_2$).

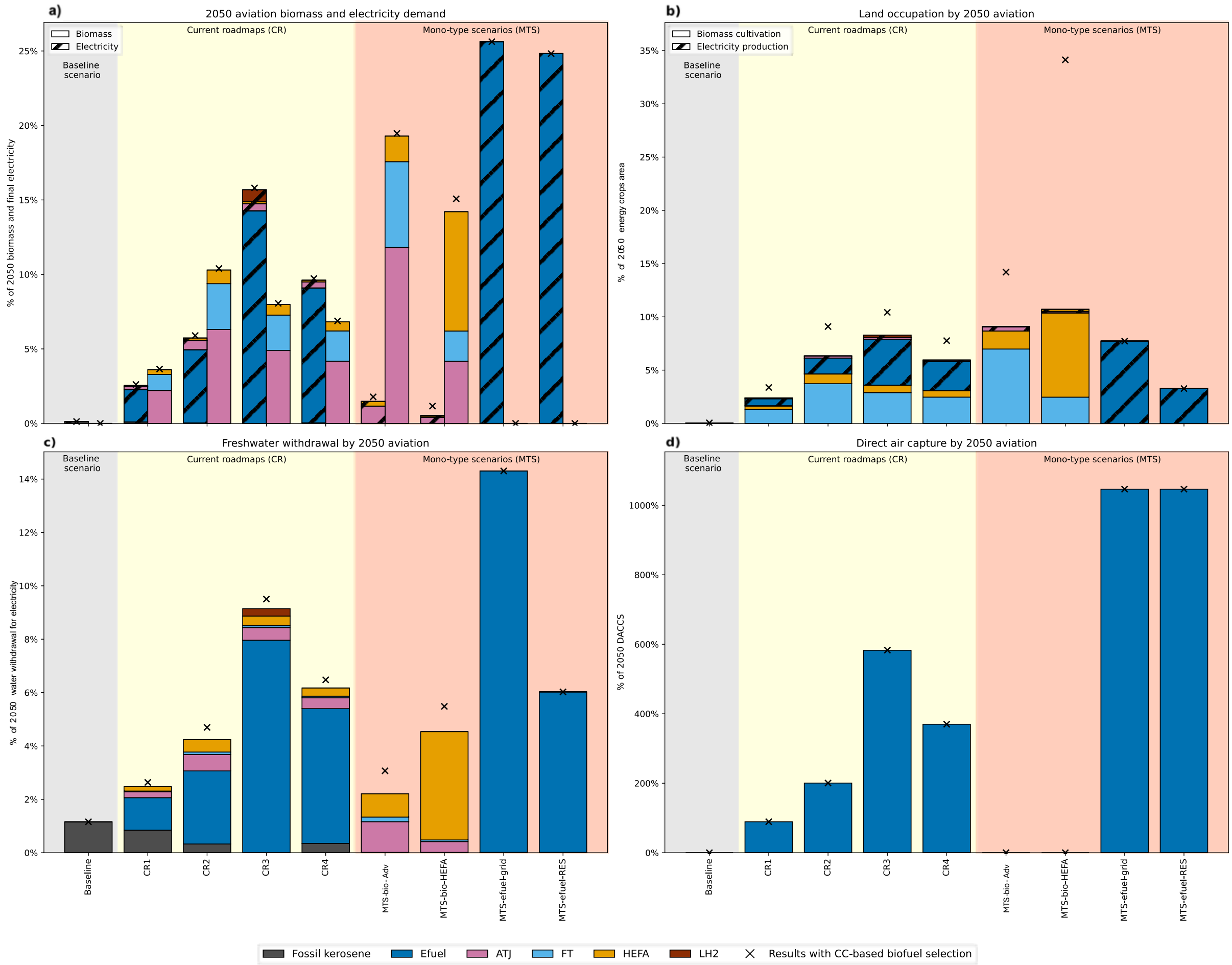

Supplementary Figure 14 - Sensitivity analysis of 2050 aviation relative resource requirements to the biofuel selection.

Sensitivity of the results of Figure 4 in the main text (relative resource requirements) is assessed for a different biofuel selection. Here the same analysis as in Figure 4 is carried out for the relative resource requirements, but using the PB-based biofuel selection instead of the climate-based selection. Black crosses represent results with the climate-based biofuel selection. The PB-based selection results in lower impacts on land occupation and freshwater use, primarily due to the higher surface yield of jatropha compared to pennycress. Panels show results for **a.** biomass and electricity (in EJ); **b.** land occupation (in Mkm^2); **c.** freshwater withdrawal (in km^3); **d.** Direct Air Capture (DAC, in $MtCO_2$).

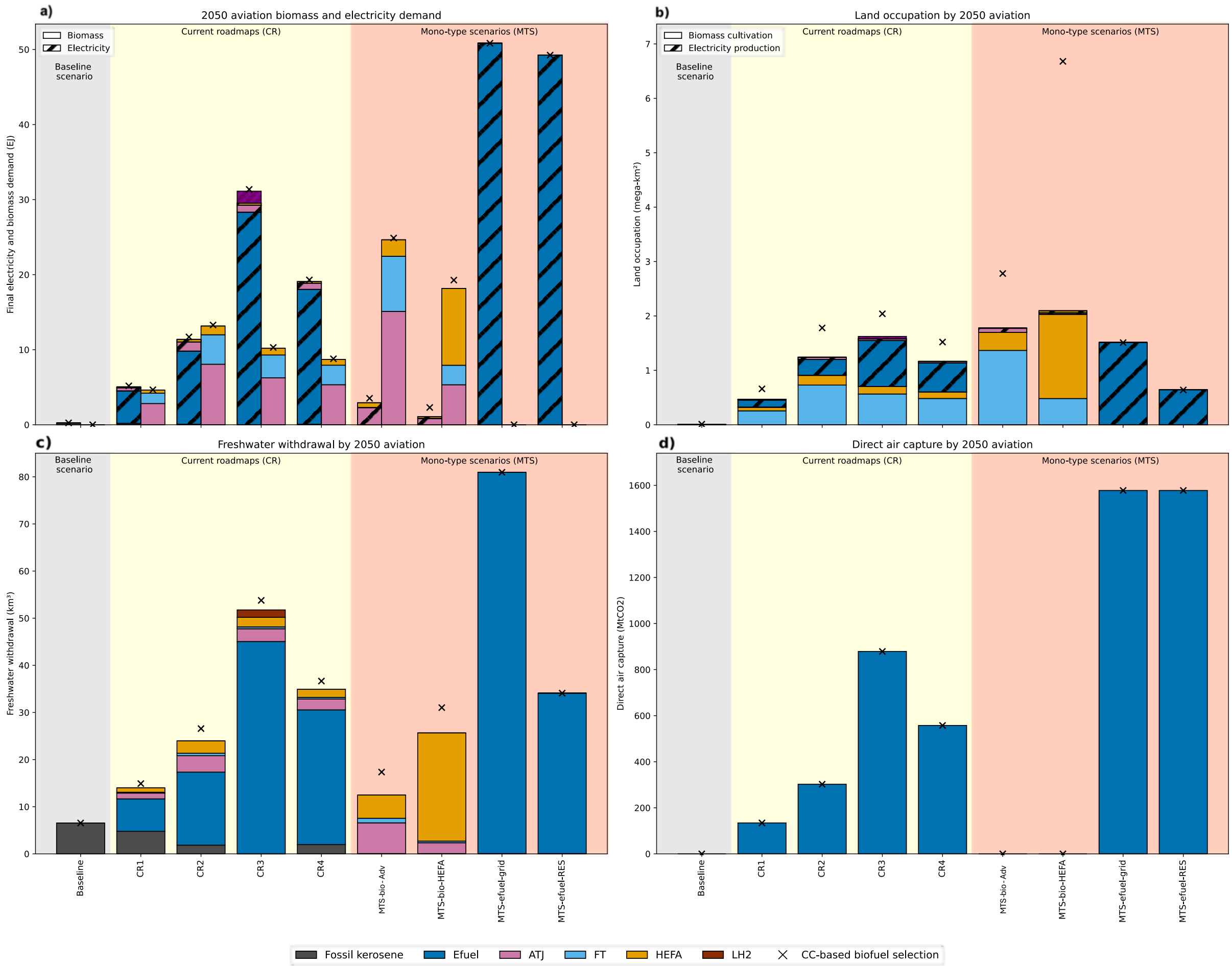

Supplementary Figure 15 - Sensitivity analysis of 2050 aviation absolute resource requirements to the biofuel selection.

Sensitivity of the results of Figure 4 in the main text (absolute resource requirements) is assessed for a different biofuel selection. Here the same analysis as in Figure 4 is carried out for the absolute resource requirements, but using the PB-based biofuel selection instead of the climate-based selection. Black crosses represent results with the climate-based biofuel selection. Panels show results for **a.** biomass and electricity (in EJ); **b.** land occupation (in Mkm^2); **c.** freshwater withdrawal (in km^3); **d.** Direct Air Capture (DAC, in $MtCO_2$).

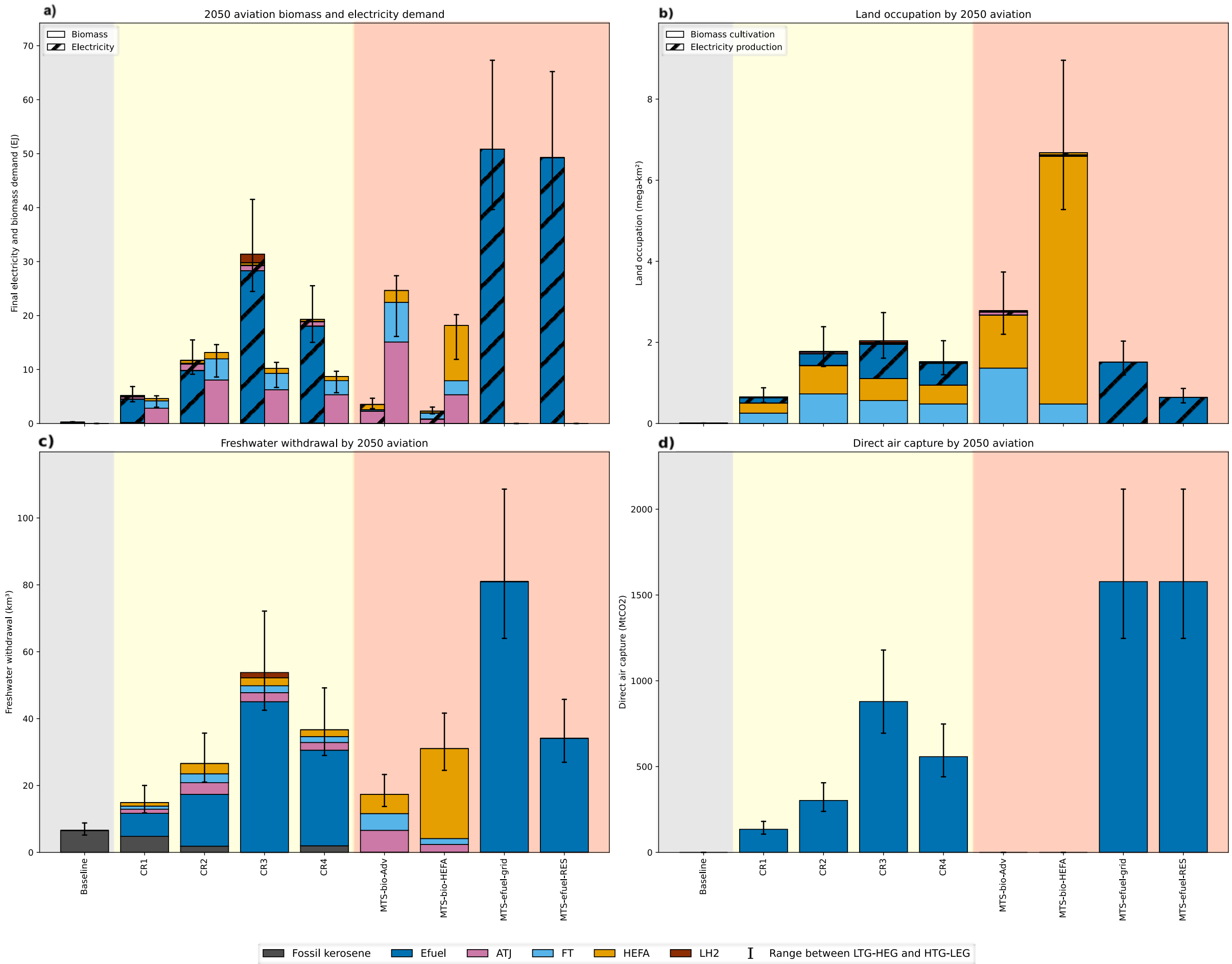

Supplementary Figure 16 - Sensitivity analysis of 2050 aviation absolute resource requirements to traffic growth rates and efficiency gains.

Sensitivity of the results of Figure 4 in the main text (absolute resource requirements) is assessed when the annual traffic growth rate (set at 3.7% yr^{-1} in the main text) and efficiency gains (set at 1.5% yr^{-1} in the main text) is varied. Traffic is here varied between 2.8% yr^{-1} and 4.2% yr^{-1} and efficiency gains between 0.9% yr^{-1} and 1.5% yr^{-1}. Intervals represent the resulting variation of the impact. Panels show results for **a.** biomass and electricity (in EJ); **b.** land occupation (in Mkm^2); **c.** freshwater withdrawal (in km^3); **d.** Direct Air Capture (DAC, in $MtCO_2$).

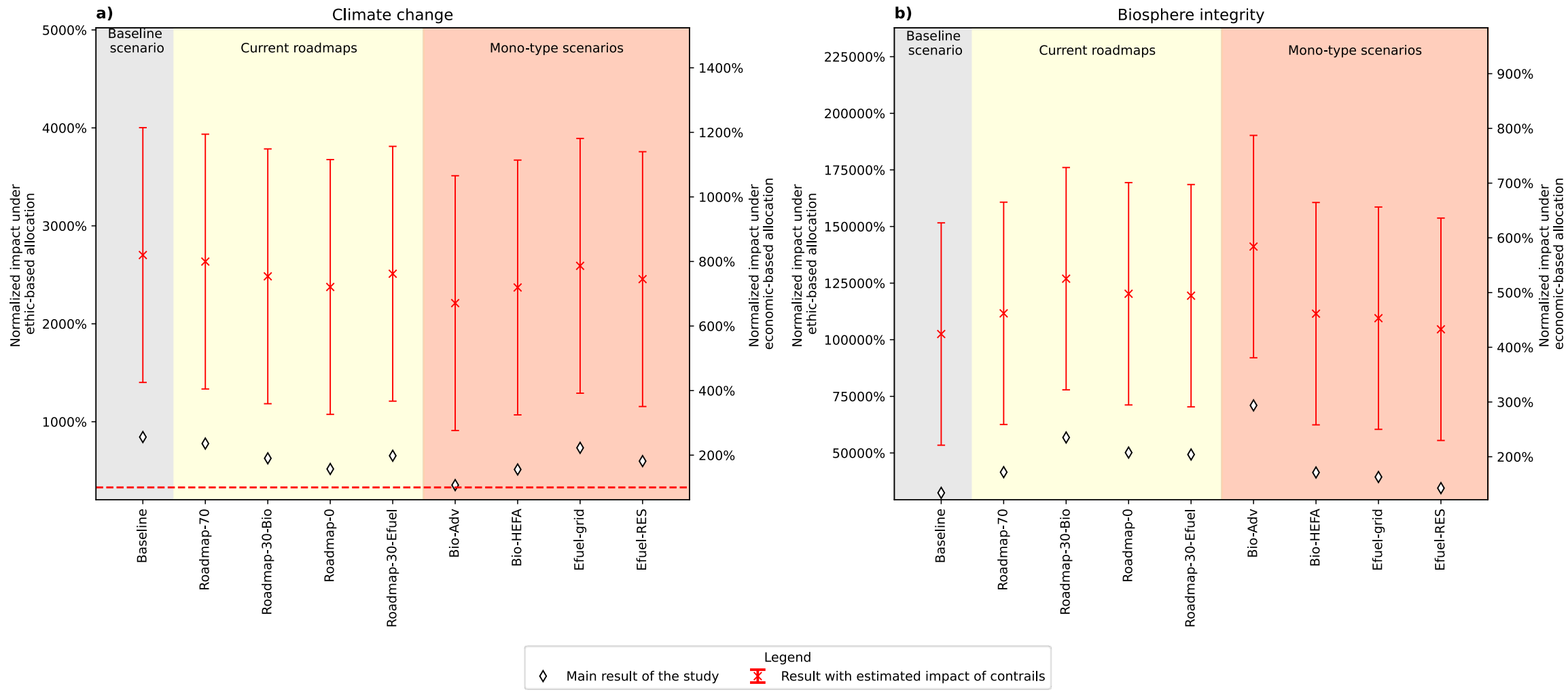

Supplementary Figure 17 – Estimates of impacts on climate change and biosphere integrity when contrail effects are taken into account by applying factors of Lee et al.[33].

Increase of the impact on **a**. climate change and **b**. biosphere integrity when contrail cirrus non-CO_2 effects are taken into account (see details and caveats below). Diamonds represent values reported in the main text (without non-CO_2 effects) while the bars above the diamonds represent the range of impact when contrail cirrus effects are considered. To perform this sensitivity analysis, we used the ERF coefficient for contrail cirrus on a km basis from Lee et al.[33] of $9.36\text{x}10^{-10}$ $mW.m^{-2}.km^{-1}$, applied to the flight-track kilometres of our 2050 scenario (174×10^{9} km). A sensitivity range of −70% to +70% was applied following the uncertainty bounds reported in Lee et al.[33]. Due to lack of results linking aviation non-CO2 impacts to planetary boundaries other than climate change, we only account for contrail cirrus impacts on climate change and biosphere integrity (which is partly driven by climate impacts). These results show that when contrail effects are taken into account, impact on climate change and biosphere integrity may be increased by significant amounts (from +30% to +900% with the above methodology, depending on the scenario and the planetary boundary considered). It is important to keep in mind that applying the contrail cirrus ERF coefficient of Lee et al.[33] to prospective scenarios faces several methodological limitations. In particular, the following effects are known to potentially affect the impacts of non-CO_2 effects: (i) climate impacts of cirrus depend not only on the volume of air traffic, but also on how it is geographically and temporally distributed[42]; and (ii) alternative energy carriers may have different impacts than fossil kerosene on non-CO_2 effects, especially on contrail formation and induced radiative forcing, but there are still significant uncertainties[43]. These potential effects are not assessed in the above estimates, which thus warrants further investigation.

Supplementary Note 7 - Sharing the SOS: Fulfillment of Human Needs

Climate change

The calculation of the share of the CC PB is based on the fact that there is a very strong correlation between consumption and climate impact (greenhouse gases emissions). The methodology for allocating a share to the transport sector follows closely the work of Heide et al.[44]:

$$\text{Share}_{\text{CC,transport}} = \text{SC}_{\text{mean,transport}} \tag{13}$$

$$\text{SC}_{\text{mean,transport}} = \frac{1}{N}\sum_{i}^{N} \frac{\text{Household}_{\text{spendings}_{\text{transport},i}}}{\text{Household}_{\text{spendings}_{\text{total},i}} + \text{Government}_{\text{spendings}_{\text{total},i}}} \tag{14}$$

where $\text{Share}_{\text{CC,transport}}$ is the share of the CC PB allocated to transport, $\text{SC}_{\text{mean,transport}}$ is the mean share of spendings for transport in the reference countries (see Supplementary Table 9), N is the number of reference countries with data available on government and household spendings (11 countries), $\text{Household}_{\text{spendings}_{\text{transport},i}}$ is the household spendings for transports in the country i, $\text{Household}_{\text{spendings}_{\text{total},i}}$ is the total household spendings in the country i and $\text{Government}_{\text{spendings}_{\text{total},i}}$ is the total government spendings in the country i.

The share for transport (and for all other categories) is then adjusted with the share for food from the EAT-Lancet Commission report[45] (see Heide et al.[44] for more details). The share of the CC PB allocated to transport is then equal to 9.5%.

The calculation of the share of aviation within transport must be based on a common unit. Here, we selected the Revenue Passenger Kilometer (RPK). To be consistent with our prospective approach, we calculated the average share of air transport in total transport (in terms of RPK) between 2015 and 2050 modelled by IMAGE-SSP2-RCP2.6 (the air transport RPKs data are not available for REMIND), that is equal to 9.1%.

$$\text{Share}_{\text{CC}} = \text{SC}_{\text{mean,transport}} * \frac{1}{36}\sum_{t=2015}^{2050} \frac{\text{RPK}_{\text{aviation}}(t)}{\text{RPK}_{\text{transport}}(t)} = 0.9\% \tag{15}$$

Other PBs

Here, since the correlation of impacts with expenditure is weaker, we consider the sectoral impacts contribution normalized by the total impacts of countries. For this purpose, Heide et al.[44] use the Input-Output (I/O) data from EXIOBASE, which provides data distributed among 163 different activity sectors. The use of I/O data is crucial here because it allows to obtain consumption-driven impacts (impacts on the territory minus exported impacts plus imported impacts). However, only one of the 24 selected countries has available data in EXIOBASE, and only on three PBs (nitrogen cycle, freshwater, and land-system change).

We fill those gaps by conducting bibliographic research for footprint (I/O) data for each PB and for each country[46–51] (see Supplementary Table 8). Once this data is collected, we perform the following three steps to compute the SoSOS assigned to aviation: (i) calculate the aviation impact of the country (domestic flights + flights departing from the country[52]; see traffic data in Supplementary Table 8) using dynamic PB-LCIA; (ii) determine the ratio of aviation impact to the total impact of the country; (iii) average across all considered countries. The calculation is expressed in the supplementary equation (16):

$$\text{Share}_{\text{PB}} = \frac{1}{N}\sum_{i=1}^{N}\frac{\text{Impact}_{\text{aviation}_{\text{country}_{i,\text{PB}}}}}{\text{Total}_{\text{impact}_{\text{country}_{i,\text{PB}}}}} \quad (16)$$

where Share_{PB} is the share of PB allocated to aviation, $\text{Impact}_{\text{aviation}_{\text{country}_{i,\text{PB}}}}$ is the impact of domestic flights and flights departing from the country *i* on PB (calculated using the data from Supplementary Table 8) and $\text{Total}_{\text{impact}_{\text{country}_{i,\text{PB}}}}$ is the total impact of the country *i* on PB (Supplementary Table 7).

Supplementary Table 7 - Footprint data for each PB and for each reference country.
For each of the 24 reference countries retained under the ethic-based allocation, the table lists the footprints on each planetary boundary. Acronyms: FWU (freshwater use), P (phosphorus cycle), N (nitrogen cycle), SOD (stratospheric ozone depletion), BI (biosphere integrity). Sources for each footprint are indicated at the top of each column.

	FWU	P	N	SOD	BI
**source**	Mekonnen et Hoekstra [46]	Metson, Bennett et Elser[48]	Oita et al.[49]	Tian et al.[50]	Phillips et al.[51]
**unit**	m^3/year	kgP cap^{-1} yr^{-1}	kgN cap^{-1} yr^{-1}	Mt CO_2-eq	BII% yr^{-1}
**year**	2011	2007	2010	2012	Average 2004-2014
**Albania**	4,8E+09	4.0	1.8E+01	6.0E-01	-3.7E-01
**Algeria**	4.9E+10	2.1	1.6E+01	4.8	1.4E-02
**Armenia**	4.4E+09	3.0	1.1E+01	5.0E-01	-1.4E-01
**Costa Rica**	5.9E+09	3.2	1.7E+01	9.0E-01	-2.5E-01
**Dominican Republic**	1.2E+10	2.5	2.0E+01	2.6	-1.5E-01
**Fiji**	1.4E+09	2.3	1.7E+01	2.0E-01	-7.1E-02
**Jordan**	8.3E+09	2.3	1.8E+01	1.1	X
**Philippines**	1.1E+11	1.9	1.2E+01	9.3	-3.9E-01
**Samoa**	3.7E+08	3.3	X	1.0E-01	-1.0E-01
**Sri Lanka**	2.4E+10	1.1	7.3	1.0	-3.9E-01
**Tunisia**	2.1E+10	2.3	1.8E+01	1.9	-2.7E-01
**Botswana**	3.5E+09	1.8	6.1E+01	1.6	-1.9E-02
**Cuba**	1.9E+10	2.6	2.1E+01	3.8	1.4E-01
**Dominica**	1.8E+08	3.8	X	X	-2.0E-01
**Egypt**	9.5E+10	2.2	X	1.6E+01	X
**Gabon**	1.8E+09	2.2	1.5E+01	2.0E-01	-7.8E-03
**Grenada**	2.2E+08	2.4	X	X	2.6E-01
**Indonesia**	2.3E+11	1.2	1.8E+01	5.9E+01	-3.8E-01
**Jamaica**	4.4E+09	2.7	2.1E+01	4.0E-01	6.3E-02
**Peru**	2.8E+10	1.6	2.4E+01	1.3E+01	-1.7E-01
**Republic of Moldova**	5.4E+09	2.4	3.2	3.0E-01	7.3E-02
**Saint Lucia**	2.5E+08	3.8	X	X	X
**Suriname**	6.3E+08	2.2	2.7E+01	1.0E-01	-6.7E-03
**Uruguay**	7.1E+09	3.3	9.4E+01	1.0E+01	-2.5E-01

Supplementary Table 8 - 2019 traffic by country (domestic + flights departing from the country).
Acronyms: ASK=Available Seat Kilometers; RPK=Revenue Passenger Kilometers.

Country	ASK 2019 (AeroSCOPE)[52]	RPK 2019 (AeroSCOPE, load factor =0.824)
**Albania**	2,04E+09	1.63E+09
**Algeria**	1.44E+10	1.15E+10
**Armenia**	3.43E+09	2.74E+09
**Costa Rica**	1.05E+10	8.42E+09
**Dominican Republic**	2.57E+10	2.06E+10
**Fiji**	5.40E+09	4.32E+09
**Jordan**	1.24E+10	9.94E+09
**Philippines**	8.95E+10	7.16E+10
**Samoa**	8.10E+08	6.48E+08
**Sri Lanka**	1.88E+10	1.51E+10
**Tunisia**	1.24E+10	9.92E+09
**Botswana**	3.77E+08	3.02E+08
**Cuba**	1.62E+10	1.29E+10
**Dominica**	2.88E+07	2.31E+07
**Egypt**	5.20E+10	4.16E+10
**Gabon**	1.31E+09	1.05E+09
**Grenada**	6.38E+08	5.10E+08
**Indonesia**	1.51E+11	1.21E+11
**Jamaica**	9.11E+09	7.29E+09
**Peru**	3.88E+10	3.10E+10
**Republic of Moldova**	2.45E+09	1.96E+09
**Saint Lucia**	2.25E+09	1.80E+09
**Suriname**	1.48E+09	1.19E+09
**Uruguay**	3.85E+09	3.08E+09

Supplementary Table 9 - Share of household and government spendings allocated to the transport sector in the reference countries, adjusted with the food share from EAT-Lancet report.

Reference countries	% allocated to transport
Albania	4.7%
Algeria	9.4%
Armenia	5.3%
Costa Rica	10.3%
Dominican Republic	7.5%
Fiji	5.4%
Jordan	14.7%
Philippines	14.2%
Samoa	5.8%
Sri Lanka	17.6%
Tunisia	9.8%
**Average**	**9.5%**

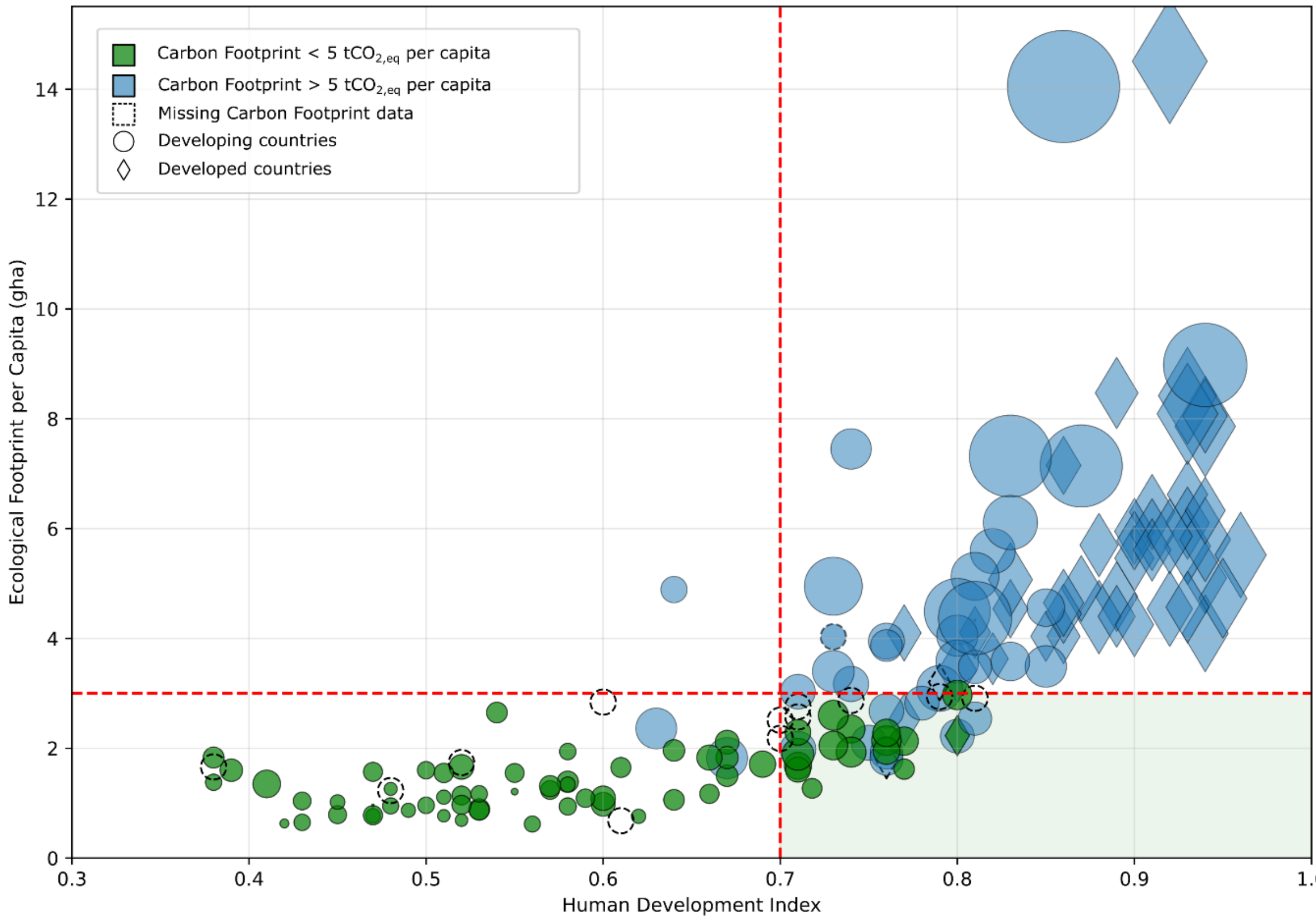

Supplementary Figure 18 - Performance of countries worldwide on the three indicators used for selecting reference countries in FHN.

Each symbol represents a country: diamonds represent developed countries and circles represent developing countries, according to the classification of the UN Conference on Trade and Development. For each country, the x-axis reports the value of the Human Development Index[53], the y-axis the value of the Ecological Footprint (EF) per capita[53] and the color of the symbol indicates whether the limit for the Carbon Footprint per capita[54] (5 $tCO_{2,eq}.cap^{-1}$) is respected (green) or not (blue). Dashedlines represent the thresholds used in the FHN selection process. Thus, the 24 reference countries are the symbols lying in the south-east orthant (HDI > 0.7 and EF per capita < 3 $gha.cap^{-1}$) with a carbon footprint per capita < 5 $tCO_{2,eq}.cap^{-1}$.

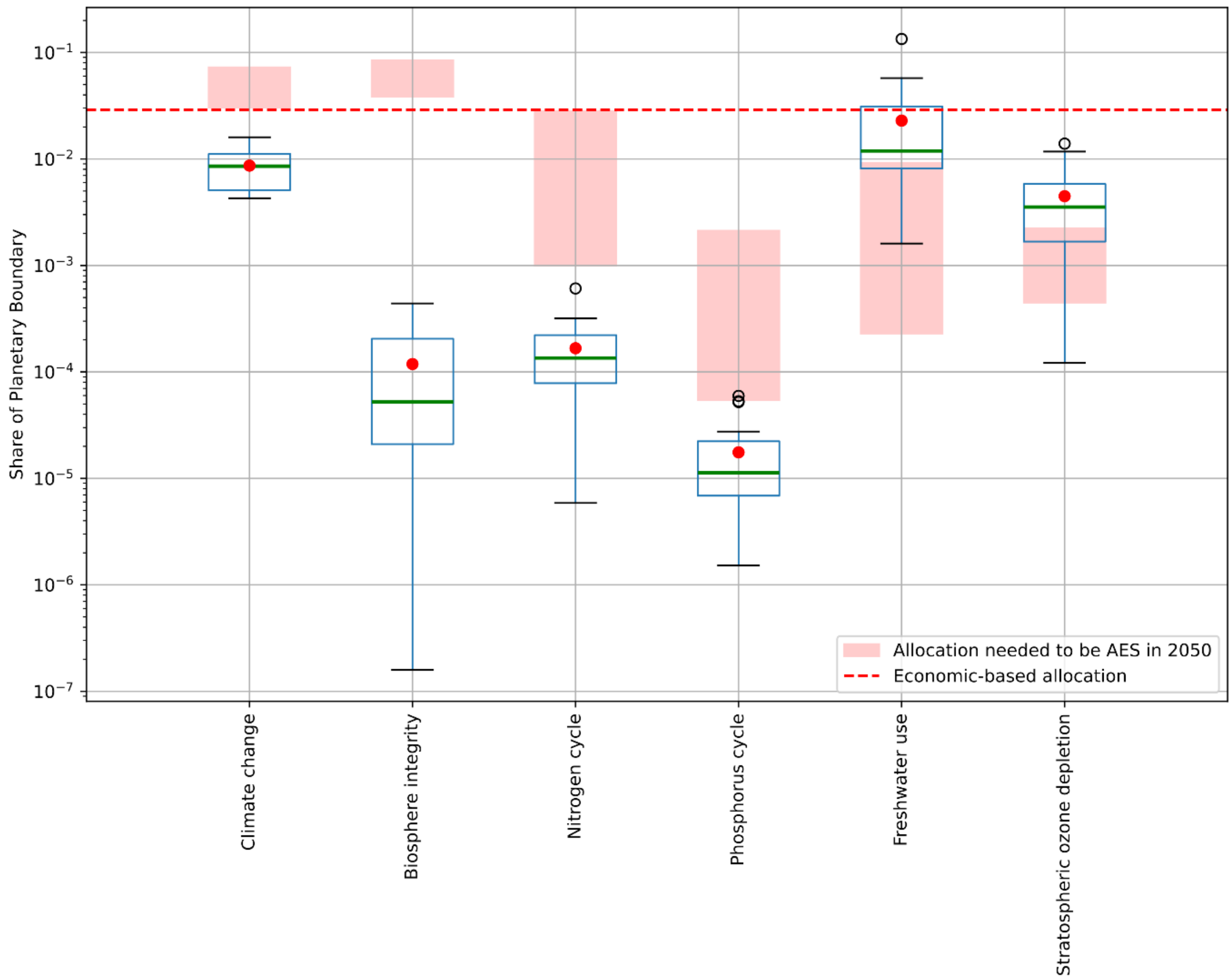

Supplementary Figure 19 - Sensitivity analysis of the FHN share allocated to aviation. The original FHN publication recommends using, as share of safe operating space for each planetary boundary (PB), the average over reference countries of the relative impact of aviation on this PB for this country. This central (average) value is represented by a red dot on the figure, and, in accordance with FHN, is the value used in the main text. For each PB, this figure displays as boxplots the 24 values (corresponding to the 24 countries in Supplementary Table 7) from which each average is computed, thereby analysing the dispersion around the mean. In the box-plots, the centre line represents the median, box limits the 25th and 75th percentiles, whiskers extend to 1.5 times the interquartile range, and individual points beyond the whiskers are shown as outliers. The economic based allocation (2.9%) is indicated by the dashed line, showing that, except for the 5% highest values for freshwater use, the relative impact of aviation across reference countries is lower than the economic based allocation. Furthermore, the pink area represents the minimal share to allocate to the aviation sector in order to respect the given PB, with the range of this area capturing variations across the nine considered scenarios. We see that, except for one outlier for phosphorus, computed shares across reference countries for the transgressed PBs (climate change, biosphere integrity, phosphorus and nitrogen) are systematically lower than the lower range of the pink area. This means that even if one were to consider the largest relative impact instead of the mean, the conclusion of the main text about the transgression of PBs would remain unchanged.

Supplementary References

1. International Civil Aviation Organization (ICAO), Long-Term Aspirational Goal (LTAG). *Report on the Feasibility of a Long-Term Aspirational Goal (LTAG) for International Civil Aviation CO2 Emission Reductions*. https://www.icao.int/environmental-protection/LTAG/Pages/LTAGreport.aspx (2022).
2. ATAG. *Waypoint 2050 - Balancing Growth in Connectivity with a Comprehensive Global Air Transport Response to the Climate Emergency: A Vision of Net-Zero Aviation by Mid-Century*. https://aviationbenefits.org/media/167417/w2050_v2021_27sept_full.pdf (2021).
3. Blanshard, A., McCurdy, M., Reid-Kay, A. & Chokhani, S. *Fueling Net Zero - How the Aviation Industry Can Deploy Sufficient Sustainable Aviation Fuel to Meet Climate Ambitions*. https://aviationbenefits.org/media/167495/fueling-net-zero_september-2021.pdf (2021).
4. Planès, T., Delbecq, S. & Salgas, A. AeroMAPS: a framework for performing multidisciplinary assessment of prospective scenarios for air transport. *J. Open Aviat. Sci.* **1**, (2023).
5. Qiu, Y. *et al.* Environmental trade-offs of direct air capture technologies in climate change mitigation toward 2100. *Nat. Commun.* **13**, 3635 (2022).
6. Meunier, N., Chauvy, R., Mouhoubi, S., Thomas, D. & De Weireld, G. Alternative production of methanol from industrial CO2. *Renew. Energy* **146**, 1192–1203 (2020).
7. Bisinella, V., Hulgaard, T., Riber, C., Damgaard, A. & Christensen, T. H. Environmental assessment of carbon capture and storage (CCS) as a post-treatment technology in waste incineration. *Waste Manag.* **128**, 99–113 (2021).
8. Gerloff, N. Comparative Life-Cycle-Assessment analysis of three major water electrolysis technologies while applying various energy scenarios for a greener hydrogen production. *J. Energy Storage* **43**, 102759 (2021).
9. Antonini, C. *et al.* Hydrogen from wood gasification with CCS – a techno-environmental analysis of production and use as transport fuel. *Sustain. Energy Fuels* **5**, 2602–2621 (2021).

10. Al-Qahtani, A., Parkinson, B., Hellgardt, K., Shah, N. & Guillen-Gosalbez, G. Uncovering the true cost of hydrogen production routes using life cycle monetisation. *Appl. Energy* **281**, 115958 (2021).

11. Antonini, C. *et al.* Hydrogen production from natural gas and biomethane with carbon capture and storage – A techno-environmental analysis. *Sustain. Energy Fuels* **4**, 2967–2986 (2020).

12. Zhang, J., Ling, B., He, Y., Zhu, Y. & Wang, Z. Life cycle assessment of three types of hydrogen production methods using solar energy. *Int. J. Hydrog. Energy* **47**, 14158–14168 (2022).

13. Björnsson, L. & Ericsson, K. Emerging technologies for the production of biojet fuels from wood—can greenhouse gas emission reductions meet policy requirements? *Biomass Convers. Biorefinery* **14**, 7603–7622 (2024).

14. Capaz, R. S., Posada, J. A., Osseweijer, P. & Seabra, J. E. A. The carbon footprint of alternative jet fuels produced in Brazil: exploring different approaches. *Resour. Conserv. Recycl.* **166**, 105260 (2021).

15. Bedin, G. Comparative Life Cycle Assessment of Sustainable Aviation Fuels: Country-based study of Alcohol-to- Jet Pathways at the European level. (Università Degli Studi Di Padova, 2024).

16. Wang, X. *et al.* Process design, modeling and life cycle analysis of energy consumption and GHG emission for jet fuel production from bioethanol in China. *J. Clean. Prod.* **389**, 136027 (2023).

17. Almena, A., Siu, R., Chong, K., Thornley, P. & Röder, M. Reducing the environmental impact of international aviation through sustainable aviation fuel with integrated carbon capture and storage. *Energy Convers. Manag.* **303**, 118186 (2024).

18. Cavalett, O. & Cherubini, F. Unraveling the role of biofuels in road transport under rapid electrification. *Biofuels Bioprod. Biorefining* **16**, 1495–1510 (2022).

19. Rojas-Michaga, M. F. *et al.* Sustainable aviation fuel (SAF) production through power-to-liquid (PtL): A combined techno-economic and life cycle assessment. *Energy Convers. Manag.* **292**, 117427 (2023).

20. Lai, Y. Y., Karakaya, E. & Björklund, A. Employing a Socio-Technical System Approach in Prospective Life Cycle Assessment: A Case of Large-Scale Swedish Sustainable Aviation Fuels. *Front. Sustain.* **3**, (2022).

21. Real Guimarães, H. *et al.* Decentralization of sustainable aviation fuel production in Brazil through Biomass-to-Liquids routes: A techno-economic and environmental evaluation. *Energy Convers. Manag.* **276**, 116547 (2023).

22. Barbera, E. *et al.* Techno-economic analysis and life-cycle assessment of jet fuels production from waste cooking oil via *in situ* catalytic transfer hydrogenation. *Renew. Energy* **160**, 428–449 (2020).

23. Liu, H. *et al.* Environmental and techno-economic analyses of bio-jet fuel produced from jatropha and castor oilseeds in China. *Int. J. Life Cycle Assess.* **26**, 1071–1084 (2021).

24. Mousavi-Avval, S. H. & Shah, A. Life cycle energy and environmental impacts of hydroprocessed renewable jet fuel production from pennycress. *Appl. Energy* **297**, 117098 (2021).

25. Ryberg, M. W., Owsianiak, M., Richardson, K. & Hauschild, M. Z. Development of a life-cycle impact assessment methodology linked to the Planetary Boundaries framework. *Ecol. Indic.* **88**, 250–262 (2018).

26. Shehab, M., Moshammer, K., Franke, M. & Zondervan, E. Analysis of the Potential of Meeting the EU's Sustainable Aviation Fuel Targets in 2030 and 2050. *Sustainability* **15**, 9266 (2023).

27. O'Malley, J., Pavlenko, N. & Searle, S. *Estimating Sustainable Aviation Fuel Feedstock Availability to Meet Growing European Union Demand*. 19 https://theicct.org/sites/default/files/publications/Sustainable-aviation-fuel-feedstock-eu-mar2021.pdf (2021).

28. Trenberth, K. E. & Smith, L. The Mass of the Atmosphere: A Constraint on Global Analyses. *J. Clim.* **18**, 864–875 (2005).
29. Shine, K. P., Fuglestvedt, J. S., Hailemariam, K. & Stuber, N. Alternatives to the Global Warming Potential for Comparing Climate Impacts of Emissions of Greenhouse Gases. *Clim. Change* **68**, 281–302 (2005).
30. Joos, F. *et al.* Carbon dioxide and climate impulse response functions for the computation of greenhouse gas metrics: a multi-model analysis. *Atmos. Chem. Phys.* **13**, 2793–2825 (2013).
31. Ramaswamy, V. *et al.* Radiative Forcing of Climate Change. (2001).
32. IPCC. Climate Change 2021: The Physical Science Basis. 2409 (2021).
33. Lee, D. S. *et al.* The contribution of global aviation to anthropogenic climate forcing for 2000 to 2018. *Atmos. Environ.* **244**, 117834 (2021).
34. IPBES. Global assessment report on biodiversity and ecosystem services of the Intergovernmental Science-Policy Platform on Biodiversity and Ecosystem Services. (2022) doi:10.5281/zenodo.6417333.
35. Galán-Martín, Á. *et al.* Sustainability footprints of a renewable carbon transition for the petrochemical sector within planetary boundaries. *One Earth* **4**, 565–583 (2021).
36. Hanafiah, M. M., Hendriks, A. J. & Huijbregts, M. A. J. Comparing the ecological footprint with the biodiversity footprint of products. *J. Clean. Prod.* **37**, 107–114 (2012).
37. Ryberg, M. W., Wang, P., Kara, S. & Hauschild, M. Z. Prospective Assessment of Steel Manufacturing Relative to Planetary Boundaries: Calling for Life Cycle Solution. *Procedia CIRP* **69**, 451–456 (2018).
38. IPCC. Climate Change 2013: The Physical Science Basis. Contribution of Working Group I to the Fifth Assessment Report of the Intergovernmental Panel on Climate Change. (2013) doi:10.1017/CBO9781107415324.
39. Byers, E. *et al.* AR6 Scenarios Database. (2022).

40. Graham, N. T. *et al.* Water Sector Assumptions for the Shared Socioeconomic Pathways in an Integrated Modeling Framework. *Water Resour. Res.* **54**, 6423–6440 (2018).
41. Sacchi, R. Premise Dashboard. https://premisedash-6f5a0259c487.herokuapp.com/ (2023).
42. Teoh, R. *et al.* Global aviation contrail climate effects from 2019 to 2021. *Atmos. Chem. Phys.* **24**, 6071–6093 (2024).
43. Lee, D. S. *et al.* Uncertainties in mitigating aviation non-CO2 emissions for climate and air quality using hydrocarbon fuels. *Environ. Sci. Atmos.* **3**, 1693–1740 (2023).
44. Heide, M., Hauschild, M. Z. & Ryberg, M. Reflecting the importance of human needs fulfilment in absolute sustainability assessments: Development of a sharing principle. *J. Ind. Ecol.* **27**, 1151–1164 (2023).
45. Willett, W. *et al.* Food in the Anthropocene: the EAT–Lancet Commission on healthy diets from sustainable food systems. *The Lancet* **393**, 447–492 (2019).
46. Mekonnen, M. & Hoekstra, A. National water footprint accounts: The green, blue and grey water footprint of production and consumption. Volume 1: Main Report. *Daugherty Water Food Glob. Inst. Fac. Publ.* https://digitalcommons.unl.edu/wffdocs/85 (2011).
47. Arto, I., Cazcarro, I., Garmendia, E., Ruiz, I. & Sanz, M. J. A new accounting framework for assessing forest footprint of nations. *Ecol. Econ.* **194**, 107337 (2022).
48. Metson, G. S., Bennett, E. M. & Elser, J. J. The role of diet in phosphorus demand. *Environ. Res. Lett.* **7**, 044043 (2012).
49. Oita, A. *et al.* Substantial nitrogen pollution embedded in international trade. *Nat. Geosci.* **9**, 111–115 (2016).
50. Tian, W., Wu, X., Zhao, X., Ma, R. & Zhang, B. Quantifying global CH4 and N2O footprints. *J. Environ. Manage.* **251**, 109566 (2019).

51. Phillips, H. *et al.* The Biodiversity Intactness Index - country, region and global-level summaries for the year 1970 to 2050 under various scenarios. (2021) doi:10.5519/HE1EQMG1.

52. Salgas, A., Sun, J., Delbecq, S., Planès, T. & Lafforgue, G. Compilation and Applications of an Open-Source Dataset on Global Air Traffic Flows and Carbon Emissions. *J. Open Aviat. Sci.* **2**, (2024).

53. Footprint Data Foundation, York University Ecological Footprint University & Global Footprint Network. National Footprint and Biocapacity Accounts. (2025).

54. Moran, D., Kanemoto, K. & Geschke, A. Carbon Footprint of Nations (1990-2018), from the Eora MRIO. (2021).
