# Supplementary material for: Current aviation roadmaps are not within planetary boundaries
Source: Nat Commun. 2026 Oct 13;17:10736. doi: 10.1038/s41467-026-77472-6 (PMC13662196; doi:10.1038/s41467-026-77472-6)
Supplement: Supplementary file 2 [file 41467_2026_77472_MOESM2_ESM.pdf]

nature portfolio

Peer Review File

Current aviation roadmaps are not within planetary boundaries

Corresponding Author: Professor Florian Simatos

This file contains all reviewer reports in order by version, followed by all author rebuttals in order by version.

Version 0:

Reviewer comments:

Reviewer #1

(Remarks to the Author)
Comments:
1. It is recommended to add a sentence in the abstract specifically describing the "model and methodological innovations" of this paper.
2. Climate change is a global issue rather than a problem confined to individual nations. Therefore, there are concerns regarding whether the 24 countries selected for this study adequately represent the overall changes occurring within the aviation industry. Furthermore, all 24 countries chosen based on the Human Development Index, Ecological Footprint, and Carbon Footprint are developing nations. Their representativeness among all countries worldwide also raises questions.
3. This paper primarily allocates the aviation sector's "safety space share" based on GDP contributions, while also considering RPK proportions in climate change allocation shares. However, aviation emissions are not confined to a single country's territory. While takeoff and landing phases can be geographically defined, flights may traverse multiple nations during cruising. Determining how to attribute emissions across different countries makes allocation based solely on GDP and RPK potentially inappropriate.
4. In the Discussion section, the authors emphasize that "non-CO_2 effects are not taken into account." However, this study demonstrates that regardless of the chosen pathway or allocation principle, the two core planetary boundaries—climate change and biosphere integrity—have already been breached. Therefore, incorporating non-CO_2 research raises two issues: (1) Would the conclusions remain unchanged? (2) Given that the CO_2 studies already reached the aforementioned conclusions, does incorporating non-CO_2 data add meaningful value to research on aviation's ability to achieve climate goals? Therefore, it is recommended that the conclusion more explicitly highlight the potential magnitude and practical implications of this limitation.
5. In Fig. 2 (d-f), even if the benchmark for the allocation principle is not exceeded, dashed lines labeled "Share of SOS under ethic-based allocation" and "Share of SOS under economic-based allocation" should still be drawn.
6. In Fig. 3, the bottom border of the legend box is missing, and the border size should be adjusted according to the number of different indicators in the legend.
7. In Table 1, the "Source of LCI" column on the right overlaps with the row numbers. We recommend modifying this.
8. The Supplementary Information document section of this paper is highly detailed. However, while page numbers are indicated for each section within the Summary, the actual materials lack these page references. Consequently, it is impossible to directly locate the required figures and tables based on the page numbers provided in the Summary.
9. Numerous figures and Supplementary Figures appear repeatedly in the Results section, negatively impacting the reading experience. The paper should clearly indicate in advance which materials can be found in the Supplementary Figures. Similar to the previous comment, upon reading the text, one discovers that certain materials are duplicated in the Supplementary Information document. However, without page numbers provided, it remains difficult to locate the required figures or tables promptly.

Reviewer #2

(Remarks to the Author)
The manuscript presents an ambitious and timely study addressing aviation sustainability within the framework of planetary boundaries. The integration of life cycle assessment (LCA) with planetary boundary (PB) concepts is novel and valuable.

However, the paper is difficult to follow due to its complex structure, unclear definitions (especially of roadmaps), and heavy reliance on supplementary materials. Major revision is required before publication.

Major Comments

1. Clarity and Structure: The manuscript contains rich data but is overly complex. Essential definitions and figures are buried in the supplementary materials. Key information—particularly the assumptions and definitions of aviation roadmaps—should be moved into the main text to improve readability.
2. Definition of Roadmaps: The scenarios (e.g., Roadmap-70, Roadmap-0, Roadmap-30-bio) are insufficiently defined. Include a concise table in the main text summarizing each roadmap's assumptions, composition, and distinguishing features.
3. Methodological Transparency: The Dynamic PB-LCIA method is said to build on Ref. 32, but modifications or innovations are unclear. Missing environmental impacts (e.g., contrails, NO_x, ocean acidification, land-system change) raise questions about comprehensiveness. Discuss these limitations and quantify uncertainties where possible.
4. Assumptions and Data Inputs: Some assumptions appear unrealistic—for instance, the claim that cryogenic and conventional aircraft have identical efficiency. Clarify how anomalies (e.g., COVID-19) were treated in the 1940–2022 dataset and explain the meaning of dashed/solid lines and colored regions in Fig. 1.
5. Updated Information: Background details are outdated. As of March 2025, 11 SAF pathways are approved under ASTM D7566/D1655. Please update accordingly.
6. Organization and Formatting: Improve structure and presentation. Move key figures (e.g., Figs. 1–2) into the main text, fix numbering inconsistencies, and ensure captions are complete and self-contained.
7. Conclusions: The paper lacks a clear Conclusion section. Summarize main numerical results, policy implications, and the most viable roadmaps for sustainable aviation.
8. Data Representation: Tables SI-8 and SI-9 focus on underdeveloped regions while omitting major emitters. Clarify the representativeness of the dataset.

Minor Comments

- Ensure that all references (e.g., Lee et al., L321 P7) are properly cited and numbered.
- Clarify what "SI 6a" refers to (figure or table) in Equation (1), P7.
- Improve the readability of Fig. 2, ensuring consistency with the textual discussion.

Reviewer #3

(Remarks to the Author)
Dear Authors,

Thank you for a timely and very interesting manuscript. Your study focuses on one of the fastest-growing sectors globally, as well as a notorious hard-to-abate industry – the discussion about the prospective impacts of aviation is therefore key, and benefits substantially from work like yours. Furthermore, the AESA perspective proves to be a compelling way of analyzing the prospective impacts of commercial aviation. We have comments we hope you can address.

Disclaimer: we were offered by the Editor to write this review as a group, and will therefore use "we" in this review.

General comments:

- The final aviation demand calculated from your assumptions may vary significantly from the demand calculated in the IAMs you use. This is not clear enough in the paper, but to calculate their total energy consumption, IAMs probably have their own assumptions about end-use sectors, is this the case? Your results show that aviation may end up using a substantial share of final energy by 2050 and beyond, so the global energy mix itself may be affected by a variation in demand. We suspect that this may have discernible effects on the results.

- The increase in electrical production necessary to reach levels required for some scenarios does not appear as clearly as it should, as well as the required DACCS amount. We would recommend putting forth the results from Figure 3 more clearly in the paragraph "Roadmaps with ambitious climate targets require significant amounts of resources in 2050" as it will also show even better your transition to reconsider the growth hypothesis.

- On allocation: we recommend that you consider applying dynamic allocation (shares as projections) aligned with IAM assumptions to ensure consistency with the dynamic life cycle inventory. However, we recognize this will add a substantial workload, please at least mention that this could be a relevant addition to the method used.

Detailed comments:

- Line [14] "transgression of six of the nine planetary boundaries"
o Should be seven, as acidification has now also been transgressed. See the recent Planetary Health Check 2025 from the PBScience project.

- Line [31-33] "We advance state-of-the-art methodologies to enable assessment of biosphere integrity and consideration of the entire 2020-2050 timespan, and we perform the first AESA proposing quantitative budget shares calculated on the basis of ethical considerations"
o We see a risk of overclaiming here and suggest softening the text here.

- Line [86-88] “Results are reported for a development storyline broadly consistent with the Paris Agreement goals (i.e., SSP 2-RCP 2.6) while results for three other storylines spanning a wide range of possible futures are considered in the Supplementary Information”

o No clear explanation about the exclusion of other SSPs. SSP2-NDC or PkBudg – could have been interesting to investigate.

- Line [107-108] “We address such shortcomings by evaluating the absolute amount of resources required in the different scenarios in comparison to the amount of resources available in the underlying development storyline.”
o Meaning difficult to follow.

- Line [284-285] “Moreover, we developed a new method to assess impacts on biosphere integrity in this dynamic context (see Dynamic PB-LCIA with biosphere integrity).”
o Claim of a “new method” for biosphere integrity appears overstated. In the SM, it seems to be an updated equation; we suggest rephrasing as a methodological extension to comply with the prospective nature of the study.

- Line[345-348] “The economic-based share of 2.9% corresponds to the direct, indirect and induced contribution of aviation to the global gross domestic product in 201851.”
o We would recommend that, ideally, the economic-based share be considered in terms of its dynamic evolution over time to comply with the dynamic system boundary of the LCI of the study. Since in that case, it will rely on forecasts, (even though retrievable) from the underpinning IAM, it remains an assumption. Adding a remark here that why static allocation was applied could be acceptable as well.

- Line[349-352] “For the computation of the ethic-based shares,…”until the end of the paragraph
o The description here is entirely focused on how the approach was applied. Consider elaborating on why such approach is ethical? And further justification or connection to steps.

- Line[350-352] “based on the following criteria: Human development index > 0.7; Ecological footprint < 3 gha cap-1 yr-1; Carbon footprint < 5 tCO2eq cap-1 yr-1.”
o If there is a basis for the definition of the thresholds, please add to the manuscript. e.g. why is the HDI cut-off threshold 0.7?

- Line[353] “These criteria lead to the selection of 24 countries (Supplementary Table SI 9)”
o Not sure it’s Table SI 9, which contains only 12 countries, can you please check?

- Line [354] “a specific approach for climate change and another approach for the other PBs.”
o Is there a reason why Climate change PB did not follow impact-based allocation as the rest? Maybe for data limitations? Consider stating the reason.

- Citation: As well as for the supplementary material the citation format is not consistent. Acronyms are both implied (10) and spelled out(11).
o We suggest that you use a program to automatically and consistently update citations with the same formatting.

Figures and tables:

- Table 1
o The source of the e-fuel LCI is from 2014. We are wondering if substantial improvements upon the processes compared to 2014 would change the inventory.
o Compared to table SI 6: In the manuscript only the “climate-based” selection is investigated. If there is a specific reason why the “PB-based” solution are not considered, it should be stated clearly.

- Figure 3
o It seems like the legend box is cut, missing the bottom half.

- Figure 4
o The icon for climate change is not the same as the ethic-based allocation, the economic based allocation and the figure legend

Supplementary material:

- Table SI 3: AEC is used for Alkaline Electrolysis Cell, in part two about Alternative Energy Carriers, also abbreviated as AEC.
o We suggest that AEC is reserved for the Altrenative Energy Carriers, as it is used in the manuscript as well.

- Table SI 5: for HEFA, Capaz et al. Used cooking oil sum of rank is 216
o The right sum is 16.

- Figure SI 6: Electricity mixes come from premise dashboard.
o Premise versions have different assumptions and give different results. We suggest that you add the version.

Reviewer #4

(Remarks to the Author)
I co-reviewed this manuscript with one of the reviewers who provided the listed reports. This is part of the Nature Communications initiative to facilitate training in peer review and to provide appropriate recognition for Early Career Researchers who co-review manuscripts.

Reviewer #5

(Remarks to the Author)
I co-reviewed this manuscript with one of the reviewers who provided the listed reports. This is part of the Nature Communications initiative to facilitate training in peer review and to provide appropriate recognition for Early Career Researchers who co-review manuscripts.

Version 1:

Reviewer comments:

Reviewer #1

(Remarks to the Author)
Comments:
1. In the response, the authors state that the selected targets correspond to social groups that perform well in both environmental sustainability and social desirability. However, it remains unclear whether the 24 developing countries selected in the study genuinely satisfy these two criteria. Additionally, it is not demonstrated whether developed countries perform worse than the selected developing countries in these dimensions. Otherwise, this may give readers the misleading impression that developing countries systematically outperform developed countries in both environmental sustainability and social desirability, or that only developing countries are capable of meeting these criteria.
2. The authors acknowledge that the FHN allocation method "tends to maintain the status quo and may not be favorable to the development of future technologies," which is an important and reasonable point. However, the response does not fully address the reviewer's core concern. First, the authors only acknowledge this limitation qualitatively, without assessing the extent to which this methodological issue may affect the study's conclusions. For instance, under scenarios where aviation transitions toward biofuels or e-fuels, its impacts on planetary boundaries such as nitrogen cycles and land use are likely to increase significantly. In this context, the FHN method—by allocating extremely small shares based on the current structure (e.g., 0.002% for phosphorus)—may systematically amplify the extent of "boundary transgression." The authors do not evaluate whether such bias could alter the main conclusions. Second, it would be beneficial to consider introducing alternative allocation methods or conducting sensitivity analyses to improve the robustness of the results.
3. The authors emphasize in their response that the study focuses on the allocation of the Safe Operating Space (SOS), rather than on the attribution of aviation emissions. However, this clarification does not fully address the reviewer's main concern regarding the appropriateness of the chosen allocation metrics (GDP and RPK). Regardless of the object of allocation, the fundamental issue remains how to define the environmental share that should be attributed to the aviation sector. The use of GDP and RPK as proxies for "social utility" and "transport utility," respectively, is debatable. GDP does not reflect the necessity or equity of different economic activities, while RPK merely measures the scale of transport activity, ignoring key aspects such as travel purpose, substitutability, and distributional inequality. In a sector like aviation, which is highly unequal and partially characterized by luxury demand, these indicators may systematically overestimate its justified environmental share.
4. In response to Comment 4, the authors only discuss the impact of non-CO_2 effects qualitatively, without providing any quantitative estimates or range analysis. Even if substantial uncertainties exist, providing a plausible range based on existing literature or conducting a simple sensitivity analysis would be essential for assessing the robustness of the results. The current lack of quantitative information makes it difficult for readers to evaluate the actual impact of this omission on the study's conclusions, particularly regarding the magnitude of boundary transgression and related policy implications.
5. In the main text (Line 60), the authors state that the annual improvement rate of aviation energy efficiency is $1.5\%\cdot yr^{-1}$, indicating that this value corresponds to the median of ICAO scenarios. However, the caption of Figure 1 uses $1.7\%\cdot yr^{-1}$ as the assumed parameter for the same scenario. Since both values are used to describe the same demand pathway for 2023–2050, the authors are encouraged to clarify which value is actually implemented in the model. If different sources or scenario settings are involved, these should be explicitly explained in the main text and presented consistently.
6. In the Supplementary Information, the "Figures" section of the Summary contains a large number of Figure SI items. It is recommended to group these figures into thematic categories (e.g., AESA, resource requirements, etc.) to improve readability. This would also facilitate more precise referencing in the main text.
7. In Figure SI 7 of the Supplementary Information, the legend includes both green and red color ranges, whereas the data displayed in the figure appear to fall entirely within the green range, with no values in the red region. This inconsistency between the legend and the actual data distribution may increase the cognitive burden for readers and potentially lead to confusion regarding the meaning of the unused color range. It is therefore suggested to adjust the color legend accordingly, for example by adopting a single-hue gradient or redefining the color scale based on the actual data range. This would allow the legend to more accurately reflect the data distribution and improve both the readability of the figure and the clarity of differences.

Reviewer #3

(Remarks to the Author)
Dear Authors,

Thank you for a substantially revised manuscript. The methodological novelty is better articulated in the revised version, and the results are clearly presented. The study will be valuable for both policymakers and researchers interested in the AESA framework. The comments were well answered. We have no major concerns; however, we suggest the following minor revisions to further strengthen the manuscript.

General comments:
Scenarios F1-F3 and S1-S3 are referenced often but only explained through the references and citations.
A table or a paragraph linking the scenarios to a quick description would improve the comprehension, understanding that S1 is the baseline and S3 aggressive sustainable aviation fuel deployment and how they can be linked to the Roadmaps.

Detailed comments

- Line [59]: “12 trillion MJ”.
o In Figure 1, the y axis is graduated in steps of 0.5e13 MJ, when it could just be in 5e12 MJ, or even better, use "12 PJ" in the text and 5 PJ for the y-axis ticks.

- Line [60-61]: “These assumptions correspond to the central values of the ICAO scenarios and are consistent with the Waypoint 2050 roadmaps”.
o 3.7%.yr-1 of increase in traffic is indeed a central value of the ICAO and could be considered as consistent with the Waypoint roadmaps, however the 1.5%.yr-1 improvements in efficiency is not a central value of the ICAO but is consistent with the Waypoint roadmaps.
We suggest that the sentence is reworked to better explain the choices of those values and how they fit into the scenarios.

- Line [74] Figure 1 caption: “efficiency improvements (1.7 %.yr-1)”.
o We assume that the actual value is the one cited in the text (1.5 %.yr-1) but it should be the same or explained why the values are different.

- Line [140] Table 3: For the monofuel scenarios, the use of “=” for the roadmap on which the scenarios are based is not clear.
o We suggest that it be replaced by “N/A” if it’s non applicable, or “RefuelEU” if it’s the same scenario as the one above that is used.

- Line [140] Table 3: For Efuel-RES comments “100% efuef from renewable energy” should be corrected as “efuel”.

- Figure 3 and 4: While distinguishable with a screen, the 3 alternative energy carriers in green are very hard to tell apart on paper.
o We recommend that the authors check with black and white filters the readability of the graph.

- Figure 3: The comparison to baseline, using only one decimal makes it so that 3a) is showing x0.8 for significantly different results for Roadmap-30-bio and Roadmap-0.
o We recommend that the comparison be expanded to two decimals, if possible, to better represent the comparison in between scenarios as well.

- Line [664]: “As explained in the original publication, there thresholds”
o Does “the original publication” refers to reference 22? We recommend adding it to clear any doubt the reader might have.
o “There” should probably be “those”.

Reviewer #4

(Remarks to the Author)
I co-reviewed this manuscript with one of the reviewers who provided the listed reports. This is part of the Nature Communications initiative to facilitate training in peer review and to provide appropriate recognition for Early Career Researchers who co-review manuscripts.

Reviewer #5

(Remarks to the Author)
I co-reviewed this manuscript with one of the reviewers who provided the listed reports. This is part of the Nature Communications initiative to facilitate training in peer review and to provide appropriate recognition for Early Career Researchers who co-review manuscripts.

Version 2:

Reviewer comments:

Reviewer #3

(Remarks to the Author)
Dear Authors,

Thank you for the additional revisions. The modifications increase the readability of the paper, and the graphs are now well presented. The comments were well answered. We have no major concerns and no minor revisions on the manuscript.

For the record here's the point-by-point checklist:
1: The addition is satisfactory and help understanding the scenarios without being redundant.
2: The readability has clearly been improved!
3: The clarification is good.
4: Already addressed through Reviewer1
5: Completely clears the confusion
6: Typo corrected
7: Very significant improvement in grayscale, colour and colourblind mode
8: I do believe that it would only benefit a) and not the other graphs, and in the actual paper it doesn’t look weird since the rounding goes to different values
9: OK

Best regards

Reviewer #4

(Remarks to the Author)
I co-reviewed this manuscript with one of the reviewers who provided the listed reports. This is part of the Nature Communications initiative to facilitate training in peer review and to provide appropriate recognition for Early Career Researchers who co-review manuscripts.

Reviewer #5

(Remarks to the Author)
I co-reviewed this manuscript with one of the reviewers who provided the listed reports. This is part of the Nature Communications initiative to facilitate training in peer review and to provide appropriate recognition for Early Career Researchers who co-review manuscripts.

Open Access This Peer Review File is licensed under a Creative Commons Attribution 4.0 International License, which permits use, sharing, adaptation, distribution and reproduction in any medium or format, as long as you give appropriate credit to the original author(s) and the source, provide a link to the Creative Commons license, and indicate if changes were made.
In cases where reviewers are anonymous, credit should be given to 'Anonymous Referee' and the source.
The images or other third party material in this Peer Review File are included in the article’s Creative Commons license,

unless indicated otherwise in a credit line to the material. If material is not included in the article's Creative Commons license and your intended use is not permitted by statutory regulation or exceeds the permitted use, you will need to obtain permission directly from the copyright holder.
To view a copy of this license, visit https://creativecommons.org/licenses/by/4.0/

Current aviation roadmaps are not within planetary boundaries

NCOMMS-25-69806A-Z

Answer to reviewers

Dear Editor,

We thank you for offering us the opportunity to resubmit our work “Current aviation roadmaps are not within planetary boundaries” (manuscript NCOMMS-25-69806A-Z) to Nature Communications. We are also grateful to the referees for their constructive and generally positive feedback, and for acknowledging the high impact and novelty of our study; in particular, Reviewers #2 and #3 describe the paper as ambitious, timely, valuable, novel and very interesting, which we hope indicates that our work is well aligned with the standards of the journal.

We have carefully addressed all of the referees’ comments, which has led to a substantial rewriting of the manuscript and, we believe, a significant improvement both in its structure and in its scientific content. A detailed, point-by-point response is provided below, and we submit two versions of the revised paper and of the supplementary information document (SI), one with all modifications highlighted in red and one clean version without tracked changes.

In revising the manuscript, we have endeavored to interpret the reviewers’ comments as accurately as possible. The initial editorial decision refers to “mixed recommendations” from the referees, yet we did not identify a clear recommendation from Reviewer #1, while Reviewer #2 explicitly suggests a major revision and Reviewer #3 expresses the hope that we can satisfactorily address their comments. Our revision strategy has therefore focused on resolving all methodological and presentation issues raised in the reports.

The decision letter highlights two main limitations of the original submission: concerns about the robustness of the results, particularly regarding the modelling framework, and concerns about the readability of the manuscript. Regarding robustness, we have provided responses to every methodological comment raised by the referees:

- Most comments have led to new analyses and results, notably on uncertainty quantification and on the comparison with the underlying integrated assessment models (IAMs) (e.g., Reviewer #2, Comment #3 or Reviewer #3, General Comment #1). These additional results are presented in the Supplementary Information, as some reviewers already noted the richness and complexity of the main results, and we wished to avoid overloading the main text;
- Some comments include relevant suggestions that, to the best of our knowledge, cannot yet be implemented because of insufficient data or the absence of established methods; for these, we added explanations in the main text explaining the limitations and, where appropriate, outlining how future work could address them (e.g., Reviewer #3, General Comment #3);
- Some comments originated from misunderstandings of our approach, which we have resolved by adding clearer explanations and definitions in the main text, and by detailing these clarifications in our point-by-point response (e.g., Reviewer #1, Comments #2 and 3 or Reviewer #2, Comment #8).

Concerning readability, we have also implemented substantial changes. The presentation of our methodological approach (especially the assumptions and definitions underlying the scenarios) has been thoroughly edited and reorganized, with numerous sections rewritten for clarity and cohesion. We have also restructured and simplified several figures, and we have rewritten the Discussion section to better guide the reader through the key insights and implications of our work.

We hope that these revisions satisfactorily address the referees' and the editor's concerns, and we remain at your disposal for any further clarification.

Best regards.

The authors
B. Pais, A. Gondran, L. Hamelin and F. Simatos

Detailed point by point response

Reviewer #1

<u>Comment #1</u>
It is recommended to add a sentence in the abstract specifically describing the "model and methodological innovations" of this paper.

<u>Answer</u>
As explained in our answer to Reviewer #2, Comment #3, the main methodological innovation probably lies in the adaptation of the PB-LCIA methodology to assess a dynamic trajectory rather than an endpoint. Thus, we have added the following sentence to the abstract:

Here we integrate prospective life-cycle inventories with the planetary boundary framework to evaluate aviation roadmaps absolute impacts as time-dependent trajectories, rather than endpoint snapshots.

Note that, beyond this change, the abstract has been largely rewritten.

<u>Comment #2</u>
Climate change is a global issue rather than a problem confined to individual nations. Therefore, there are concerns regarding whether the 24 countries selected for this study adequately represent the overall changes occurring within the aviation industry. Furthermore, all 24 countries chosen based on the Human Development Index, Ecological Footprint, and Carbon Footprint are developing nations. Their representativeness among all countries worldwide also raises questions.

<u>Answer</u>
We thank the reviewer for this comment. The selection of countries pertains to the Fulfilment of Human Needs (FHN) allocation principle, referred to in the paper as the ethic-based allocation. It is important to realize that the core idea of this approach is to select countries based on well-established social and environmental indicators (namely, the human development index and the ecological and carbon footprints). The aim is not to obtain a sample representative of the aviation sector or of all countries worldwide, but rather of societies that are both environmentally sustainable (through the ecological and carbon footprints) and socially desirable (through the human development index). Within these countries, the allocation of environmental budgets across sectors is interpreted as one possible example of a sustainable distribution of environmental space. The choice of indicators used to select countries is open to discussion, but once they are chosen, the resulting set of countries follows mechanically. It is, in our view, interesting in itself, and indeed somewhat surprising (as the reviewer notes), that only developing countries simultaneously reach high standards on the three selected indicators. To clarify these points, we have added the following sentences in the methodology section when presenting the FHN principle (section "computing the ethic-based allocated shares").

It has to be noted that these countries are not representative of countries worldwide, nor do they capture changes occurring in the aviation industry. Rather, the selection process from the three social and environmental indicators aims to get countries representative of sustainable countries, from a social and environmental perspective.

This being said, we concur with the reviewer that the fact that FHN does not reflect potential future changes in the aviation industry is a shortcoming of the approach since it favors a status quo situation. This shortcoming was already mentioned in the manuscript as follows:

The very small shares observed under the ethic-based allocation for biosphere integrity, nitrogen and phosphorus cycles reflect that aviation currently relies on fossil kerosene with very low impacts on these PBs. This can be seen as a limitation of the method already pointed out in its original conceptual description, which tends to perpetuate a status quo situation potentially adverse to new technologies appearing in the future.

Despite this shortcoming, FHN is, to our knowledge, the only explicitly actionable allocation principle grounded in ethical considerations and currently available for operational AESA studies. It provides a valuable and complementary perspective to the economic allocation based on contribution to GDP and has recently stimulated intense activity within the AESA community. Our paper is the first to implement FHN at the scale of a large business sector, which we believe gives it potential as a benchmark for future work.

Comment #3

This paper primarily allocates the aviation sector's "safety space share" based on GDP contributions, while also considering RPK proportions in climate change allocation shares. However, aviation emissions are not confined to a single country's territory. While takeoff and landing phases can be geographically defined, flights may traverse multiple nations during cruising. Determining how to attribute emissions across different countries makes allocation based solely on GDP and RPK potentially inappropriate.

Answer

In the present work, we do not “attribute emissions across different countries” and so, although we totally concur with the reviewer that the question of attributing CO_2 emissions of international aviation is disputable, it does not concern our study. Rather than attributing emissions, the problem that we face is (as mentioned by the reviewer) to compute the share of the climate change safe operating space allocated to the aviation sector. For this purpose, two methods are considered: from a high-level perspective, both aim to capture the “usefulness” of the aviation sector, and none relies on attributing emissions.

In the economic-based approach, the share is simply equal to the contribution of aviation to global GDP. This approach is grounded in the classical economic interpretation of GDP as wealth, and so the contribution of an activity sector can be interpreted as its usefulness to society. Moreover, it has the very desirable feature of being easy to implement. On the downside, the usefulness interpretation is prone to critics (some useless or even arguably negative activities are accounted for in GDP), and this economic approach lacks ethical justifications and does not differentiate different planetary boundaries.

In the ethic-based approach, the basic idea is also to capture the utility of aviation to individuals. The difference is that instead of the contribution to GDP, one considers the share of household and government spendings in a set of sustainable countries meeting high environmental and social performance. However, data is only available for the amount of spendings for the whole transport sector, and not specifically for aviation. Thus, available data allows to compute a share $S_{transport}$ allocated to the transport sector, and the ratio $RPK_{aviation}/RPK_{transport}$ is then used as a proxy to define the share of $S_{transport}$ that is futher allocated to the aviation sector specifically, within the larger transport sector. The rationale for

this ratio $RPK_{aviation}/RPK_{transport}$ is that, ideally, the allocation should capture the utility to individuals. Although it is very difficult to give a single definition and metric of utility for different activity sectors, for transport, the distance travelled is a good proxy for utility as the goal of the transport sector is to move people and goods around. In the end, the share $S_{transport}$ x ($RPK_{aviation}/RPK_{transport}$) is meant to capture the utility of aviation through a mix of economic ($S_{transport}$) and social ($RPK_{aviation}/RPK_{transport}$) indicators. We note that a similar approach is made in the original publication proposing the FHN principle[1] where the share allocated to "peanuts" is a product of a share affected to the food sector, multiplied by the contribution of peanuts to the average daily coverage.

To clarify these different points, we have added the following sentences in the methods section when presenting the FHN principle (section "computing the ethic-based allocated shares").

Note that this share $Share_{CC}$ is meant to capture the utility of the aviation sector to individuals. For climate change, the original FHN publication recommends to compute it based on household and government spendings. However, this data is only available for the whole transport sector (which allows to compute the term $SC_{mean,transport}$) and so the ratio $RPK_{aviation}/RPK_{transport}$ is here to evaluate the proportion of this share that is allocated to the aviation sector specifically, within the larger transport sector. Considering RPKs for this is in line with the aim of capturing utility to individuals, since for the transport sector, this metric can indeed be seen as the end-product delivered.

Comment #4

In the Discussion section, the authors emphasize that "non-CO_2 effects are not taken into account." However, this study demonstrates that regardless of the chosen pathway or allocation principle, the two core planetary boundaries—climate change and biosphere integrity—have already been breached. Therefore, incorporating non-CO_2 research raises two issues: (1) Would the conclusions remain unchanged? (2) Given that the CO_2 studies already reached the aforementioned conclusions, does incorporating non-CO_2 data add meaningful value to research on aviation's ability to achieve climate goals? Therefore, it is recommended that the conclusion more explicitly highlight the potential magnitude and practical implications of this limitation.

Answer

We thank the reviewer for the interesting comment. Concerning question (1): yes, the conclusions regarding the transgression or not of the core planetary boundaries would remain essentially unchanged since these boundaries are transgressed, and non-CO_2 effects would only increase the impact. However, the magnitude of the transgression would be affected by the incorporation of non-CO_2 effects. Concerning question (2): yes, we believe that incorporating non-CO_2 data would be meaningful. In particular, it has been demonstrated in earlier studies that non-CO_2 effects affect the way one should define aviation's climate goals[2], and that mitigating non-CO_2 effects requires the deployment of carbon capture technologies[3].

---

[1] Heide, M., Hauschild, M. Z. & Ryberg, M., 2023, Reflecting the importance of human needs fulfilment in absolute sustainability assessments: Development of a sharing principle, Journal of Industrial Ecology

[2] See for instance Brazzola, N., Patt, A. & Wohland, J. Definitions and implications of climate-neutral aviation. Nat. Clim. Chang. 12, 761–767 (2022).

[3] See for instance Sacchi, R. et al. How to make climate-neutral aviation fly. Nat Commun 14, 3989 (2023) or Brazzola, N. et al. The role of direct air capture in achieving climate-neutral aviation. Nat Commun 16, 588 (2025)

Concerning the potential magnitude of non-CO_2 effects, we stress that they are very difficult to estimate in prospective scenarios. Indeed, it has been demonstrated that non-CO_2 effects depend on the precise traffic distribution (so that only describing traffic growth is not enough)[4] and on the evolution of atmospheric composition, which depends on the underlying RCP pathway[5]. Moreover, efuels and biofuels are expected to decrease CO_2 effects, but the precise quantification of this improvement is still uncertain[6].

Following the reviewer's suggestion, we have added the following sentences in the conclusion.

Accounting for non-CO_2 effects would not change our conclusions regarding the transgression of the core planetary boundaries, since they would only increase impacts. However, quantifying the magnitude of the transgression would help assess the amount of carbon capture technologies needed to offset aviation climate impact and reach climate goals. Quantifying non-CO_2 effects in prospective scenarios faces significant challenges because they depend on precise traffic distribution and on the evolution of ambient atmospheric composition, and also because the impacts of alternative energy carriers on non-CO_2 effects currently face significant uncertainties.

Comment #5

In Fig. 2 (d-f), even if the benchmark for the allocation principle is not exceeded, dashed lines labeled "Share of SOS under ethic-based allocation" and "Share of SOS under economic-based allocation" should still be drawn.

Answer

We thank the reviewer for the recommendation. However, we face a problem of scales:

- The line "Share of SOS under ethic-based allocation" is present in Fig. 2 (d) (in this case, the benchmark for the allocation principle is exceeded): it is drawn at the level of y = 100% on the left axis, but since values on this axis range up to 14000%, it is squized at the bottom and barely visible;
- For the rest, the reviewer is correct and, although the benchmark is not exceeded, the lines are absent. This owes to the fact that they would need to be drawn at levels y = 100% while maximal values reach 50%. If scales were changed to incorporate the y = 100% range, the bars would be significantly smaller which would make them difficult to read, especially when the maximal y-range is below 10% where their height would be divided tenfold.

Instead of drawing the lines, we have added the following precisions in the captions. We hope that this will still satisfactorily accommodate the reviewer's comment.

The dotted green line for the ethic-based share of SOS is barely visible in figures c and d because it represents the y=100% line, while the scale of the left y-axis ranges up to values of 15000%. In figures e and f, the dotted lines are not represented because impacts are below the threshold.

Comment #6

---

[4] Teoh et al., 2024, Global aviation contrail climate effects from 2019 to 2021, Atmospheric Chemistry and Physic

[5] Skowron, A. et al. Greater fuel efficiency is potentially preferable to reducing NOx emissions for aviation's climate impacts. Nat Commun 12, 564 (2021)

[6] Lee, D. et al. Uncertainties in mitigating aviation non-CO_2 emissions for climate and air quality using hydrocarbon fuels. Environmental Science: Atmospheres (2023)

In Fig. 3, the bottom border of the legend box is missing, and the border size should be adjusted according to the number of different indicators in the legend.

Answer
We thank the reviewer for the comment and have made the recommended changes (see the new Figure 4).

Comment #7
In Table 1, the "Source of LCI" column on the right overlaps with the row numbers. We recommend modifying this.

Answer
We thank the reviewer for the comment and have made the recommended changes (see the new Table 2).

Comment #8
The Supplementary Information document section of this paper is highly detailed. However, while page numbers are indicated for each section within the Summary, the actual materials lack these page references. Consequently, it is impossible to directly locate the required figures and tables based on the page numbers provided in the Summary.

Answer
We apologize for the inconvenience. As requested by the reviewer, we have added page numbers in the Supplementary Informations.

Comment #9
Numerous figures and Supplementary Figures appear repeatedly in the Results section, negatively impacting the reading experience. The paper should clearly indicate in advance which materials can be found in the Supplementary Figures. Similar to the previous comment, upon reading the text, one discovers that certain materials are duplicated in the Supplementary Information document. However, without page numbers provided, it remains difficult to locate the required figures or tables promptly.

Answer
We thank the reviewer for this feedback. Concerning the negative reading experience, upon re-reading the Results section, we felt that the problem was with the numerous references to the figure as in the sentence "*the climate change (Fig. 2a) and biosphere integrity (Fig. 2b) PBs are transgressed for all scenarios, while the stratospheric ozone depletion (Fig. 2f) and freshwater use (Fig. 2e) PBs are respected*" which, we definitely agree, is not enjoyable to read. In order to alleviate this problem, we have proceeded to a careful and thorough rewriting of many sentences in the Results section, trying to avoid this repetition. In particular, references to figures now appear much less often than in the first version of the paper.

Concerning the materials that can be found in the Supplementary Figures, we have added sentences to indicate them in advance, for instance the two sentences *"The sensitivity of these results to the choice of biofuel production pathways, assumptions on future traffic growth rate and efficiency gains, uncertainties in historical impacts. the underlying development storyline and background technosphere is assessed in Figures SI 7-11 of the Supplementary Information, and shows that our results are robust to these variations*" (line 189) and "*The same sensitivity analysis as above is performed on these results in Figures SI 12-16 of the*

Supplementary Information, and confirms the robustness of our results” (line 319) that announce the majority of figures from the SI.

Concerning the duplication of materials in the SI, we have also performed a careful inspection. The only redundancy that we could identify in the first version was between Figure 1 and Figure SI 1 that display the evolution of the aviation fuel mix over time for the scenarios analysed in the main text (Figure 1) and the institutional and industrial roadmaps that we seek to model (Figure SI 1). The reviewer is right that one roadmap was completely duplicated in the main text, namely the RefuelEU roadmap that corresponds to the Roadmap-30-Efuel scenario: therefore, we have suppressed the subfigure corresponding to RefuelEU from Figure SI 1. Nonetheless, we have chosen to keep the other figures (namely, the evolution of the fuel mix in the ATAG and ICAO scenarios) because they complement the new Figure 2 in the main text in two ways: 1/ they display the ATAG scenarios and 2/ although the ICAO roadmaps correspond to Roadmap-70, Roadmap-30-Bio and Roadmap-0 in the main text (this is now made clearer in the new version of the paper, see the new Table 3), they bring additional information by displaying the evolution of LCAF which, in the main text, is assimilated with fossil kerosene.

Finally, the reviewer also points to the problem of the absence of page numbering in the SI, which has been addressed in the previous comment.

Reviewer #2

General comment

The manuscript presents an ambitious and timely study addressing aviation sustainability within the framework of planetary boundaries. The integration of life cycle assessment (LCA) with planetary boundary (PB) concepts is novel and valuable. However, the paper is difficult to follow due to its complex structure, unclear definitions (especially of roadmaps), and heavy reliance on supplementary materials. Major revision is required before publication.

Answer

We thank the reviewer for the very positive feedback concerning the relevance of our paper. Addressing the comments of all reviewers led to a significant revision of the paper. The presentation of scenarios as well as the conclusion have been completely rewritten, figures have been re-organized and legends rewritten, additional information has been added in order to clarify the methodological approach, uncertainty quantification has been strengthened, and we have performed a global polishing of the paper in terms of writing in order to improve readability. Detailed answers to the reviewer's comments are provided below, and we hope that this major revision will satisfactorily address them.

Comment #1

Clarity and Structure: The manuscript contains rich data but is overly complex. Essential definitions and figures are buried in the supplementary materials. Key information—particularly the assumptions and definitions of aviation roadmaps—should be moved into the main text to improve readability.

Answer

Concerning the complexity of the paper, we have performed a thorough editing to simplify numerous formulations: we hope that the new version will be more accessible.

The reviewer also asks us to move key information related to the definitions of aviation roadmaps from the SI to the main text. Anticipating the next comment, we were also asked to clarify the definition of scenarios. These two comments led us to significantly rewrite the sections "Scenarios overview" and "Scenarios modeling current roadmaps" that introduce and define the scenarios analysed. In the first version of the paper, our goal in these sections was to define these scenarios in a synthetic yet complete way, referring to the SI for a justification of their assumptions based on the institutional and industrial roadmaps that we aim to model. To address the reviewer's comments #1 and #2, we have reversed the presentation: now the definition of scenarios starts from the real institutional and industrial roadmaps, and then introduces the scenarios modeling them while clarifying the modeling assumptions. To be more precise, we have moved the old Table SI 1 to the main text, and we have also updated it: the new Table 1 thus presents the main characteristics of the institutional and industrial roadmaps. The link between these roadmaps and the modeling scenarios is explicitly given in the new Table 3, which aims to address the next comment by summarizing the roadmap's assumptions. We hope that this new version, which both moves key information from the SI to the main text and clarifies the scenario definitions will satisfactorily address the reviewer's comments.

In our view, the material that is left in the SI is not essential to the understanding of the results in the main text: this material mostly presents further details on the institutional and industrial roadmaps (Fig. SI 1), precisions about underlying data (related to life cycle analysis in Fig. SI

2 and Tables SI 1-5, to the underlying integrated assessment in Fig. SI 5-6 and Table SI 6 and to the ethic-based allocation in Tables SI 7-9) and sensitivity analysis results (Figs. SI 7-16).

Comment #2

Definition of Roadmaps: The scenarios (e.g., Roadmap-70, Roadmap-0, Roadmap-30-bio) are insufficiently defined. Include a concise table in the main text summarizing each roadmap's assumptions, composition, and distinguishing features.

Answer

As explained in the answer to the previous comment, the sections "Scenarios overview" and "Scenarios modeling current roadmaps" have been rewritten to address this comment. The new Table 3 explains the link with the institutional and industrial roadmaps, which should clarify the roadmap's assumptions. Moreover, in order to highlight the composition and distinguishing features of the scenarios, we have separated the old Figure 1 into two figures: one presenting the evolution of global aviation fuel demand (new Figure 1), which is common to all scenarios, and one presenting the evolution of the AEC mix fulfilling this demand for each scenario (new Figure 2), and we have also rewritten these figures legends. We hope that these changes satisfactorily address the reviewer's comment.

Comment #3

Methodological Transparency: The Dynamic PB-LCIA method is said to build on Ref. 32, but modifications or innovations are unclear. Missing environmental impacts (e.g., contrails, NO_x, ocean acidification, land-system change) raise questions about comprehensiveness. Discuss these limitations and quantify uncertainties where possible.

Answer

We thank the reviewer for this comment, which allowed to add useful clarifications to the paper. Concerning modifications to the PB-LCIA method, we have:

- Modified the framework in order to analyse the sustainability of a dynamic trajectory, and not only of a given endpoint assumed to perpetuate for a long time;
- Included historical responsibility in the analysis;
- Incorporated the biosphere integrity assessment method of Galan-Martin et al. (in the original method of Ref. 32, biosphere integrity was not assessed), and adapted it to a dynamic setting;
- Coupled it with prospective LCA.

To make this clear, we have added the following sentences in the "Dynamic AESA methodology" section.

For the impact assessment step, we built on the PB-LCIA methodology which we extended (compared to the original publication[32]) along several directions by: 1/ adapting it to a dynamic setting; 2/ assessing impacts on biosphere integrity in this dynamic context; 3/ accounting for historical impacts; 4/ coupling it with prospective LCIs. More precisely, in the original publication, PB-LCIA was designed to evaluate a static situation (in particular, historical impacts were not accounted for) and biosphere integrity was not included in the analysis[32]. Our main methodological contribution is to extend this framework so that it can be applied to the analysis of dynamic scenarios as those we are interested in. This methodological development relies on the use of the Bern Simple Carbon Model for climate change, and the extension of the approach as Galan-Martin et al.[47] to include biosphere integrity in this dynamic context (see

“dynamic PB-LCIA with biosphere integrity” below). Analysing a dynamic trajectory allows for the incorporation of historical impacts in the assessment (see “impact assessment of historical aviation (1940-2022)” below).

Concerning the comprehensiveness of the results, we definitely agree that some aspects are left out of the analysis. Concerning non-CO_2 effects (contrails and NO_x), we refer the reviewer to our answer to Reviewer #1, Comment #4: including them would be relevant, but is very challenging in a prospective context because they depend on precise traffic distribution and on the evolution of ambient atmospheric composition, and also because the impacts of alternative energy carriers on non-CO_2 effects currently face significant uncertainties. In order to address this comment from Reviewer #1, we have added the following sentence in the conclusion, which we hope also addresses the present comment.

Accounting for non-CO_2 effects would not change our conclusions regarding the transgression of the core planetary boundaries, since they would only increase impacts. However, quantifying the magnitude of the transgression would help assess the amount of carbon capture technologies needed to offset aviation climate impact and reach climate goals. Quantifying non-CO_2 effects in prospective scenarios faces significant challenges because they depend on precise traffic distribution and on the evolution of ambient atmospheric composition, and also because the impacts of alternative energy carriers on non-CO_2 effects currently face significant uncertainties.

Concerning stock planetary boundaries such as ocean acidification, analysing them in a dynamic setting requires a dynamic model to assess their evolution over time, and in particular the persistent influence of past impacts. For climate change for instance, emitted compounds fade out with time, yielding a decreasing impact which we quantified thanks to the Bern Simple Carbon Model. However, we did not find similar models for ocean acidification. Moreover, we would not know how to compute the share of safe operating space allocated to this PB in the ethic-based allocation. Land-system change was also left out of the analysis because of a lack of robust LCA data. Indeed, as mentioned in the paper, it is important to realize that within the planetary boundaries framework, land-system change only accounts for deforestation. Although robust LCA data are available concerning land occupation, it is very uncertain to assess the part of this occupation that is linked to deforestation. We have found partial data concerning photovoltaic farms[7], but it is not enough for a full analysis as we would typically need similar data for wind turbines. It therefore seemed preferable to us to leave it out of the study. More generally, not covering all planetary boundaries is standard practice in the AESA literature. As illustrated by our compilation of PB-based and LCIA-based AESA studies published between 2019 and 2025 (subsequent figure), no existing study simultaneously assesses all planetary boundaries. Climate change and freshwater use are the most frequently covered boundaries. Ocean acidification is also commonly included in AESA studies, but exclusively in static frameworks. Land-system change is likewise often assessed; however, existing applications rely on highly uncertain inventory data which results in very low estimated impacts and limits the interpretability of the results. In contrast, atmospheric aerosol loading and the introduction of novel entities remain only sporadically or never addressed. Our boundary coverage is therefore consistent with the current state of the art in AESA research, while explicitly documenting and discussing the implications of omitted boundaries.

[7] Zhang et al., 2024, Revisiting the land use conflicts between forests and solar farms through energy efficiency, Journal of Cleaner Production

In order to address the reviewer's comment, we have:

- added the following sentence at the end of the first paragraph of the "Dynamic AESA methodology" section:
  Note that, despite the inclusion of biosphere integrity, our results do not cover the ocean acidification and land-system change PBs for reasons elaborated below.
- expanded the sentence where we mentioned the absence of ocean acidification (section "dynamic PB-LCIA with biosphere integrity"), which now reads:
  As we did not find corresponding short-term dynamic model for ocean acidification, we do not assess impacts on this PB. Moreover, to the best of our knowledge, data is not available to compute the ethic-based share of safe operating space for this PB (see "Computing allocated shares"). Note however that ocean acidification is strongly correlated with climate change.
- expanded the sentence where we mentioned the absence of land-system change (section "dynamic PB-LCIA with biosphere integrity"), which now reads:
  Impacts on land-system change are not assessed due to highly uncertain LCIs. While land occupation data are generally robust and consistent for specific technologies or processes, translating these into impacts on land-system change such as deforestation requires assumptions about land type, location, and baseline conditions that are highly uncertain at global scales. Partial data to estimate the amount of land surface that can be linked to deforestation is available for photovoltaic farms, but is not enough to carry on a full analysis.

Finally, the reviewer asked to "*quantify uncertainties where possible"*. To address this comment, we have performed Monte-Carlo simulations by drawing 1,000 samples from the uncertainty distributions provided by ecoinvent on the background technosphere. Results are displayed in the new Figure SI 7 from the Supplementary Informations where for each scenario and each PB, we have reported the probability that variations in background technosphere led to a different conclusion (in terms of transgression or respect of a PB) than in Figure 3 in the main text. We find that in the vast majority of cases (98 out of the 108 combinations of scenario/PB considered), none of the conclusion is reversed among the 1,000 draws; 7 combinations have led to reverse conclusions less than 10% of the times; and 3 combinations were more sensitive to this uncertainty, with results affected more than 15% of the times, and only under the economic-based allocation. We thus consider that these results testify for the robustness of our conclusions, and hope that this will satisfactorily address the reviewer's comment.

<u>Comment #4</u>

Assumptions and Data Inputs: Some assumptions appear unrealistic—for instance, the claim that cryogenic and conventional aircraft have identical efficiency. Clarify how anomalies (e.g., COVID-19) were treated in the 1940–2022 dataset and explain the meaning of dashed/solid lines and colored regions in Fig. 1.

<u>Answer</u>

We agree with the reviewer that the assumption about cryogenic aircraft is unrealistic. An assumption had to be made because this information is lacking in the roadmaps considered. Note however that this assumption is optimistic, in that recent studies suggest that potential

future cryogenic aircraft will likely be less efficient than conventional aircraft[8]. Moreover, this assumption has only a marginal impact on our results: cryogenic aircraft is only present in Roadmap-0, and even then it plays a minor role, accounting for 4% of the aviation 2050 energy mix. To clarify this point, we have added the following sentence in the section "scenarios modeling currend roadmaps":

Note that this is an optimistic assumption, as current forecasts for the gravimetric index of liquid hydrogen tank would lead to cryogenic aircraft being less efficient than current conventional aircraft.

Concerning the 1940-2022 dataset, we have used real traffic data from the International Air Transport Association[9], which includes the post-pandemic period since 2020. Thus, we do not see the need for any specific treatment.

Finally, we agree that the meaning of dashed and solid lines in Fig. 1 was confusing, especially since the meaning of the dashed line changed between the historical and prospective periods. We have updated the figure (which is now the new Figure 1) and the legend accordingly.

Comment #5

Updated Information: Background details are outdated. As of March 2025, 11 SAF pathways are approved under ASTM D7566/D1655. Please update accordingly.

Answer

We thank the reviewer for pointing this out. However, we could not find where this information was used in our paper.

Comment #6

Organization and Formatting: Improve structure and presentation. Move key figures (e.g., Figs. 1–2) into the main text, fix numbering inconsistencies, and ensure captions are complete and self-contained.

Answer

We have tried to comply to the reviewer's comment: all figures and tables have now been moved in the main text, and we have rewritten their captions so that they are complete and self-contained. Moreover, we have performed a careful proofreading to fix numbering inconsistencies.

Comment #7

Conclusions: The paper lacks a clear Conclusion section. Summarize main numerical results, policy implications, and the most viable roadmaps for sustainable aviation.

Answer

We thank the reviewer for the comment, which lead to a significant rewriting of the Discussion (see red text in new version).

[8] According to Adler, E. & Martins, J. Hydrogen-powered aircraft: Fundamental concepts, key technologies, and environmental impacts. Progress in Aerospace Sciences 141, 100922 (2023), gravimetric index is the main feature governing the efficiency of potential cryogenic aircraft. Values currently envisioned by 2050 suggest that cryogenic aircraft will be less efficient than conventional aircraft.

[9] Taken from IATA's Industry Statistics Fact Sheet.

Concerning the summary of the main numerical results, we have chosen to recall results for the the most ambitious roadmap for climate (Roadmap-0): this seems a relevant choice as current roadmaps are designed to mitigate climate change. Once these main numerical results are recalled, we then proceed to describing the most viable roadmaps. To do so, we have identified four main caracteristics of a sustainable roadmap based on our results: 1/ priorizing electricity production from renewable energy sources; 2/ balancing biofuel feedstock selection to optimize trade-offs across PBs; 3/ deploying CCS technologies to compensate historical impacts; and 4/ incorporating demand-side mitigation measures. We then discuss the policy implications of such roadmaps.

We stress that we did our best to accommodate the reviewer's comments while sticking to the journal's guidelines, which explicitly asks for a succinct discussion and not a conclusion[10]. Despite this tension between the reviewer's comment and the journal's guidelines, we hope that our changes will be deemed satisfactory by the reviewer.

Comment #8

Data Representation: Tables SI-8 and SI-9 focus on underdeveloped regions while omitting major emitters. Clarify the representativeness of the dataset.

Answer

We thank the reviewer for this comment: it parallels the Comment #2 from Reviewer #1, and so we refer the reviewer to our answer to this comment above.

Minor comment #1

Ensure that all references (e.g., Lee et al., L321 P7) are properly cited and numbered.

Answer

We have carefully proofread the new version, and hope that this problem has been addressed.

Minor comment #2

Clarify what "SI 6a" refers to (figure or table) in Equation (1), P7.

Answer

We thank the reviewer for this feedback: we clarified that this referred to the whole section 6a of the SI, and not a figure or table.

Minor comment #3

Improve the readability of Fig. 2, ensuring consistency with the textual discussion.

Answer

We thank the reviewer for the feedback: while re-reading the text, we realized that it was indeed sometimes difficult to find the corresponding numbers in the figure (note that the old Fig. 2 from the initial version is now the new Fig. 3). To fix this issue, we replaced the mention of the average values in the text by the range of values (the average cannot be found directly on the figure, while the range can); and we have also increased the font of the numbers indicating the comparison to baseline (as well as other fonts), so they are easier to find. We hope that this satisfactorily addresses the reviewer's comment.

---

[10] "The main text of an Article should begin with a section headed Introduction[…], followed by sections headed Results, Discussion (if appropriate) and Methods (if appropriate). […] The Discussion should be succinct and may not contain subheadings", see https://www.nature.com/ncomms/submit/article

Reviewer #3

General comment

Thank you for a timely and very interesting manuscript. Your study focuses on one of the fastest-growing sectors globally, as well as a notorious hard-to-abate industry – the discussion about the prospective impacts of aviation is therefore key, and benefits substantially from work like yours. Furthermore, the AESA perspective proves to be a compelling way of analyzing the prospective impacts of commercial aviation. We have comments we hope you can address.

Answer

We thank the reviewers for this very positive feedback, and we share the hope of having addressed their comments satisfactorily. Concerning the comments of Reviewer #3, we believe that we have addressed the General comment #2 but in our view, General comments #1 and 3 rather point to limitations of state-of-the-art AESA methodologies. Trying to address them would require significant methodological developments that reach far beyond the present paper, probably necessitating distinct publications. We have added some clarifications in the paper to emphasize these limitations, and hope that our detailed answer below will satisfy the reviewers.

General comment #1

The final aviation demand calculated from your assumptions may vary significantly from the demand calculated in the IAMs you use. This is not clear enough in the paper, but to calculate their total energy consumption, IAMs probably have their own assumptions about end-use sectors, is this the case? Your results show that aviation may end up using a substantial share of final energy by 2050 and beyond, so the global energy mix itself may be affected by a variation in demand. We suspect that this may have discernible effects on the results.

Answer

We thank the reviewer for this highly relevant comment, to which we fully concur. In the current state-of-the-art, prospective LCA frameworks do not account for feedback effects of the studied sectoral trajectories on the underlying IAM, which clearly creates a potential inconsistency when studying macroscopic sectors such as aviation. Through discussions at recent conferences, we know that some people are working on this issue, e.g., by trying to incorporate the studied trajectory within the underlying IAM. However, this is a highly non-trivial task that calls for significant developments, as IAMs are very complex software difficult to interact with (notwithstanding the fact that they are not open-source, so that partnerships and collaborations with suitable research groups are required). This limitation is common to all large-scale prospective LCA studies, including papers recently published in Nature Communications[11].

IAMs indeed make assumptions on the evolution of final energy demand across end-use sectors. However, for aviation, these assumptions remain relatively coarse. While air traffic data can be retrieved from IAM outputs, we did not identify explicit assumptions regarding the detailed energy demand of the aviation sector or its internal fuel composition in the IAM scenarios used as background.

---

[11] See for instance Sacchi, R. et al., 2023, How to make climate-neutral aviation fly, Nat Commun 14, 3989

In order to evaluate whether the aviation demand trajectory assumed in this study departs significantly from IAM assumptions, we now provide in the Supplementary Information a dedicated quantitative comparison in a new section *« 4.e -"Comparison of aviation traffic assumptions with IAM scenarios »*. Using aviation traffic projections from the AR6 Scenario Explorer[12], this section compares our assumptions with IMAGE 3.2 scenarios and reports the resulting differences in traffic and energy demand in the following table :

Scenario	Traffic growth rate (% yr^{-1})	Traffic in 2050 relative to 2023	Global aviation fuel demand in 2050 relative to 2023*	Cumulative fuel demand 2023-2050 relative to 2023*	Source
This study	3.7	2.67	1.78	37.96	-
IMAGE SSP2_2.6	3.32	2.42	1.62	35.97	Byers et al., 2022[45]
IMAGE SSP2-baseline	3.59	2.59	1.73	37.34	
IMAGE SSP1-1.9	1.94	1.68	1.12	29.70	

*Table SI 6 – Comparison of current roadmaps and IAMs assumptions. * assuming identical efficiency gains of 1.5% yr^{-1} in all scenarios*

In IMAGE SSP2-2.6 (named SSP2_SP12_26I_D) and SSP2-baseline, aviation traffic grows at average rates of 3.32% yr^{-1} and 3.59% yr^{-1}, respectively, compared to 3.7% yr^{-1} assumed in this study based on industry roadmaps. In contrast, in the stringent mitigation pathway IMAGE SSP1-1.9 (named SSP1_SPA1_19U_D), aviation traffic growth is substantially lower, at 1.94% yr^{-1}.

As documented in Section 4.e and Table SI 7, although SSP2 growth rates are close in annual terms, compounding over 2023–2050 leads to non-negligible differences. Relative to identical traffic in 2023, the assumed growth rate of 3.7% yr^{-1} results in a traffic level in 2050 about 10% higher than in SSP2-2.6 and about 3% higher than in SSP2-baseline, while it is about 59% higher than in SSP1-1.9. Assuming identical efficiency gains of 1.5% yr^{-1} in all scenarios, these differences translate directly into differences in aviation energy demand. Over the full period 2023–2050, the cumulative aviation energy demand modelled in this study is about 5.5% higher than in SSP2-2.6, 1.6% higher than in SSP2-baseline, and 28% higher than in SSP1-1.9.

This comparison highlights that aviation demand assumptions span a wide range across IAM narratives, and that high-growth aviation trajectories such as those considered here imply substantially higher long-term energy and resource requirements than mitigation-consistent pathways. This therefore reinforces the relevance of explicitly assessing the additional energy and resource demand induced by aviation relative to IAM-consistent trajectories. We hope that the addition of Section 4.e and Table SI 6 clarifies this point and satisfactorily addresses the reviewer's concern.

General comment #2

[12] Byers et al., 2022, AR6 Scenarios Database, Integrated Assessment Modeling Consortium & International Institute for Applied Systems Analysis

The increase in electrical production necessary to reach levels required for some scenarios does not appear as clearly as it should, as well as the required DACCS amount. We would recommend putting forth the results from Figure 3 more clearly in the paragraph "Roadmaps with ambitious climate targets require significant amounts of resources in 2050" as it will also show even better your transition to reconsider the growth hypothesis.

Answer

We the reviewers for the comment. In the original version, results from Figure 3 for the current roadmaps were already discussed in the main text. For instance, we already mentioned that "*ambitious aviation roadmaps would simultaneously require increasing electricity production capacities by 16%, bioenergy production and freshwater withdrawal for electricity by 10%, mobilizing a land area larger than the total area dedicated to growing energy crops, and quadrupling the DAC capacity*". In order to avoid repetition, we have added a last paragraph to discuss more specifically the efuel scenarios, which were not discussed, and where the effect pointed out by the reviewers (high electricity and DACCS requirements) are the most pronounced. Moreover, this gives more coherence with the reference to the Sacchi et al. paper[13] since they also consider a 100%-efuel scenario. The last paragraph of this section now reads as follows:

Although we concluded above based on systemic environmental performance that efuels may be preferable over biofuels, these results suggest that aggressive efuel deployment leads to questionable resource requirements. In mono-type scenarios where all the demand is met through efuel (scenarios Efuel-grid/RES), aviation needs to mobilize around 25% of the total electricity produced and almost six times the amount of DACCS in the underlying storyline. This corroborates previous findings where efuel-scenarios were studied at the European level, and seriously questions the feasibility of current roadmaps.

General comment #3

On allocation: we recommend that you consider applying dynamic allocation (shares as projections) aligned with IAM assumptions to ensure consistency with the dynamic life cycle inventory. However, we recognize this will add a substantial workload, please at least mention that this could be a relevant addition to the method used.

Answer

We thank the reviewers for the very interesting suggestion, and we concur with them that dynamic allocation is an attractive idea. However, we see several major difficulties that prevent us from considering it in our paper.

First, there is a problem of data. It is important to realize that the computation of current FHN shares does not rely on IAM output, but rather on specific publications (see Table SI 7). Finding the relevant data for the current situation was already the output of a significant literature search, and, to our knowledge, similar data projected on various IAMs is not available. In the same vein, the selection of countries in FHN may vary with time as environmental and social performance of countries evolve, but we would not know how to project the human development index and ecological footprint. The economic-based allocation faces similar (albeit weaker) obstacles: if global GDP is an output of IAM, it would still be difficult to assess

[13] Sacchi, R. et al., 2023, How to make climate-neutral aviation fly, Nat Commun 14, 3989

the specific contribution of aviation, especially since we consider its direct, indirect and induced contribution.

Second, we believe that dynamic allocation is not scientifically mature yet. For instance, a dynamic allocation may go against the goal of AESA to inform public decision: indeed, how to design a proper strategy if the constraint evolves over time? Drawing a parallel with allocating carbon budgets, how would a country design a proper decarbonization strategy if its budget were to potentially change every year? Moreover, we also see a potential problem of circular reasoning. Indeed, shares are computed depending on the behavior of the aviation sector (whether through its economic contribution or its environmental impact). Thus, in a dynamic allocation, the shares would potentially be determined by the very trajectory one seeks to analyse, which potentially goes against the principle of AESA to make a distinction between impact assessments and budget allocation. To the best of our knowledge, such issues (and, more generally, dynamic allocation) have not been considered yet in the scientific literature. Lacking a solid scientific foundation for considering dynamic allocation, and considering that the robustness of our results was one of the reasons for rejecting our paper, we think it is preferable not to consider it. Nevertheless, we concur that it represents a promising idea and so, following the reviewers recommendation, we have added the following sentence in the paper.

Note that all the shares computed above are constant with time: considering dynamic allocation aligned with assumptions of the underlying storyline could be interesting to ensure consistency with the prospective LCA approach. However, no such allocation schemes have been proposed to date, and so this represents an interesting venue for future research.

<u>Detailed comment #1</u>

- Line [14] "transgression of six of the nine planetary boundaries"

o Should be seven, as acidification has now also been transgressed. See the recent Planetary Health Check 2025 from the PBScience project.

<u>Answer</u>

We have updated accordingly.

<u>Detailed comment #2</u>

- Line [31-33] "We advance state-of-the-art methodologies to enable assessment of biosphere integrity and consideration of the entire 2020-2050 timespan, and we perform the first AESA proposing quantitative budget shares calculated on the basis of ethical considerations"

o We see a risk of overclaiming here and suggest softening the text here.

<u>Answer</u>

We thank the reviewers for the comment. We have rephrased this sentence in the following way:

To perform this AESA, we mobilized the recently established methodology developed by Ryberg et al. and Bjørn et al. consisting to combine Life Cycle Impact Assessment (LCIA) with the PB framework (PB-LCIA). Here, we adapted the PB-LCIA methodology to add (i) temporality, thereby allowing to consider the entire 2020-2050 timespan for prospective assessments, (ii) the assessment of biosphere integrity, a core PB, and (iii) rules rooted in ethical considerations to determine how to allocate a share of the safe operating space of each PB to the aviation sector.

Detailed comment #3

- Line [86-88] "Results are reported for a development storyline broadly consistent with the Paris Agreement goals (i.e., SSP 2-RCP 2.6) while results for three other storylines spanning a wide range of possible futures are considered in the Supplementary Information"

o No clear explanation about the exclusion of other SSPs. SSP2-NDC or PkBudg – could have been interesting to investigate.

Answer

We thank the reviewer for this comment and for suggesting additional SSP pathways. Our selection of storylines was guided by the objective of ensuring both consistency with aviation decarbonization narratives and a reasonable balance between scenario coverage and methodological complexity.

In the main text, we focus on SSP2–RCP2.6 because it represents a central « middle-of-the-road » socio-economic pathway combined with a mitigation stringency broadly consistent with the Paris Agreement goals, and it is widely used as a reference background in prospective LCA applications. This scenario therefore provides a natural benchmark for assessing the sustainability of aviation transition pathways under policy-relevant climate constraints.

In the Supplementary Information, we considered three additional storylines in order to span a wide range of contrasted futures. These include SSP2–NPI (REMIND), representing a weak-policy baseline with significant residual emissions, and SSP1–1.9 (REMIND), representing a stringent mitigation pathway compatible with a 1.5°C target. We also considered SSP2–2.6 variants from both REMIND and IMAGE in order to test the robustness of our results across IAM implementations under comparable mitigation stringency.

Importantly, the SSP2–2.6 and SSP1–1.9 scenarios from REMIND considered in this study are peak-budget (PkBudg) formulations, explicitly constrained by a cumulative carbon budget. These scenarios therefore already capture the dynamics associated with carbon-budget–driven mitigation pathways, which directly address the reviewer's suggestion to investigate PkBudg-type scenarios.

We did not include additional SSP2 variants such as SSP2–NDC or alternative PkBudg realizations in order to limit the number of background scenarios and preserve the readability and tractability of the analysis (recall that complexity was a criticism addressed at our paper). Moreover, the selected ensemble already covers (i) a central policy-relevant mitigation pathway (SSP2–2.6), (ii) a weak-policy baseline (SSP2–NPI), and (iii) a stringent 1.5 °C-consistent pathway (SSP1–1.9), while allowing for cross-IAM comparison.

We therefore believe that the selected set of storylines provides a representative and sufficiently contrasted range of futures to assess the robustness of our conclusions, while maintaining consistency with aviation decarbonization narratives and preserving both the readability and the tractability of the scenario ensemble.

Detailed comment #4

- Line [107-108] "We address such shortcomings by evaluating the absolute amount of resources required in the different scenarios in comparison to the amount of resources available in the underlying development storyline."

o Meaning difficult to follow.

Answer
We have rephrased the end of the paragraph, which now reads as follows:

To help evaluate the feasibility of these roadmaps, we complement the AESA by an assessment of resource requirements in terms of energy (electricity and bioenergy), land occupation, freshwater withdrawal and carbon sequestrated by direct air capture (DAC), which is required to produce efuels. The absolute amount of these resources required in 2050 is computed for each scenario, and is compared to the global availability of this resource in the underlying development storyline in order to get insight into the significance these absolute amounts (see Methods and Supplementary Figs. SI 12-16).

Detailed comment #5
- Line [284-285] "Moreover, we developed a new method to assess impacts on biosphere integrity in this dynamic context (see Dynamic PB-LCIA with biosphere integrity)."

o Claim of a "new method" for biosphere integrity appears overstated. In the SM, it seems to be an updated equation; we suggest rephrasing as a methodological extension to comply with the prospective nature of the study.

Answer
The reviewers are right that our biosphere integrity assessment method is not entirely new, and so we agree that qualifying it as a "new method" is overstated and apologize for this. The sentence that the reviewers point to was rephrased in order to comply with Comment #3 from Reviewer #2: we now mention that "we extended" the PB-LCIA methodology, and when talking about biosphere integrity, we talk about "the extension of the approach of Galan-Martin et al. to include biosphere integrity in this dynamic context", see the first paragraph in the "Dynamic AESA methodology", and our answer to Reviewer #2, Comment #3.

Detailed comment #6
- Line[345-348] "The economic-based share of 2.9% corresponds to the direct, indirect and induced contribution of aviation to the global gross domestic product in 2018."

o We would recommend that, ideally, the economic-based share be considered in terms of its dynamic evolution over time to comply with the dynamic system boundary of the LCI of the study. Since in that case, it will rely on forecasts, (even though retrievable) from the underpinning IAM, it remains an assumption. Adding a remark here that why static allocation was applied could be acceptable as well.

Answer
We refer the reviewers to our detailed answer to General comment #3 about dynamic allocation.

Detailed comment #7
- Line[349-352] "For the computation of the ethic-based shares,…"until the end of the paragraph

o The description here is entirely focused on how the approach was applied. Consider elaborating on why such approach is ethical? And further justification or connection to steps.

Answer

The ethic-based approach actually consists in implementing the fulfillment of human needs (FHN) sharing principle proposed by Heide et al.[14]. As is explicitly explained in the original paper, the goal of FHN is to operationalize the sufficientarianism principle of distributive justice theory, which aims that everyone gets enough: “The FHN sharing principle for AESAs was developed to reflect the importance of the necessities delivered to users. This principle is a first attempt to operationalize the distributive justice theory sufficientarianism for AESA. The FHN principle highlights the sectors and products that should reduce their environmental footprint most, based upon how essential the need delivered to the users is. This motivates companies to focus on producing the “right things” since non-essential products would receive an insufficient share.”

To make this clear we have added the following sentence in the “Computing allocated shares” section:

The ethic-based allocation implements the fulfillment of human needs (FHN) principle. FHN operationalizes the sufficientarianism principle of distributive justice theory (everyone should get enough) by allocating environmental burdens according to how essential the needs satisfied by different products and sectors are to users.

Detailed comment #8

- Line[350-352] “based on the following criteria: Human development index > 0.7; Ecological footprint < 3 gha cap-1 yr-1; Carbon footprint < 5 tCO2eq cap-1 yr-1.”

o If there is a basis for the definition of the thresholds, please add to the manuscript. e.g. why is the HDI cut-off threshold 0.7?

Answer

These thresholds were proposed in the original FHN publication. The authors first considered more stringent thresholds, justified in the following way:

- a threshold of 0.8 for HDI, motivated by the Human Development Report (2020) in which this threshold served to defined the highest group in terms of human development (“very high human development”)
- a threshold of 1.72 Gha/cap/yr for the ecological footprint, considered in O’Neill et al.[15] aiming at quantifying resource use associated with meeting basic human needs;
- a threshold of 985 kgCO_2-eq/cap/yr for CO_2 emissions, considered in Bjørn and Hauschild[16] as the carrying capacity for climate change.

Thus, these values of thresholds (0.8, 1.72 Gha/cap/yr and 985 kgCO_2-eq/cap/yr) are justified. However, no country satisfies these three thresholds simultaneously, which is why higher thresholds are considered in the FHN publication. In order to clarify this, we have added the following sentence in the “computing allocated shares” section:

As explained in the original publication, there thresholds correspond to proposed values for sustainability (for instance, a human development index above 0.8 corresponds to the “very

---

[14] Heide, M., Hauschild, M. Z. & Ryberg, M., 2023, Reflecting the importance of human needs fulfilment in absolute sustainability assessments: Development of a sharing principle, Journal of Industrial Ecology

[15] O'Neill, D. et al., 2018, A good life for all within planetary boundaries, Nature Sustainability 1

[16] Bjørn, A. and Hauschild, M., 2015, Introducing carrying capacity-based normalisation in LCA: framework and development of references at midpoint level, Life Cycle Sustainability Assessment 20

high human development group"), which have been relaxed in order to get a sufficient number of countries simultaneously passing the bar for the three indicators.

Detailed comment #9

- Line[353] "These criteria lead to the selection of 24 countries (Supplementary Table SI 9)"

o Not sure it's Table SI 9, which contains only 12 countries, can you please check?

Answer

We thank the reviewers for the careful reading, there was a mistake corrected in the new version of the paper.

Detailed comment #10

- Line [354] "a specific approach for climate change and another approach for the other PBs."

o Is there a reason why Climate change PB did not follow impact-based allocation as the rest? Maybe for data limitations? Consider stating the reason.

Answer

We thank the reviewer for this comment. The use of a specific approach for climate change follows directly the sharing framework proposed by Heide et al.[17], which distinguishes climate change from the other PBs. In their methodology, climate change allocation is based on sustainable consumption patterns and economic expenditure, because energy use accounts for the majority of global greenhouse gas emissions and CO_2 emissions are strongly correlated with consumption across most sectors. In contrast, impacts on the other PBs vary strongly across sectors, making a uniform allocation across PBs inappropriate. For this reason, Heide et al. propose to use expenditure-based sharing for climate change and impact-based sharing for the other PBs using the status quo principle. Since the robustness of our paper was already questioned in some comments and by the editor, we prefer to stick to the original, peer-reviewed framework rather than making another methodological development. We have clarified these points in the revised manuscript by adding the following sentence in the « Computing the ethic-based allocated shares » section:

This distinction follows the original framework of Heide et al., who propose an expenditure-based sharing for climate change because CO_2 emissions are strongly correlated with consumption across most sectors, while impacts on the other planetary boundaries exhibit large sectoral heterogeneity and therefore require impact-based sharing to reflect these differences.

Detailed comment #11

- Citation: As well as for the supplementary material the citation format is not consistent. Acronyms are both implied (10) and spelled out(11).

o We suggest that you use a program to automatically and consistently update citations with the same formatting.

Answer

[17] Heide, M., Hauschild, M. Z. & Ryberg, M., 2023, Reflecting the importance of human needs fulfilment in absolute sustainability assessments: Development of a sharing principle, Journal of Industrial Ecology

We thank the reviewers for this comment: we have carefully proofread the new version and hope that citation format is now consistent.

<u>Detailed comment #12</u>

- Table 1

o The source of the e-fuel LCI is from 2014. We are wondering if substantial improvements upon the processes compared to 2014 would change the inventory.

o Compared to table SI 6: In the manuscript only the "climate-based" selection is investigated. If there is a specific reason why the "PB-based" solution are not considered, it should be stated clearly.

<u>Answer</u>

We thank the reviewer for these comments. Concerning the first question, while the foreground structure of the e-fuel life cycle inventory originates from a 2014 reference, the overall environmental performance of synthetic fuels in our framework is primarily driven by upstream energy supply (most notably electricity production) rather than by the fuel processing step itself (here Fischer–Tropsch). This reflects the fact that the dominant energy requirements stem from water electrolysis and carbon capture, which largely determine electricity, heat and water consumption.

To clarify this point quantitatively, we provide below an additional contribution analysis (not included in the main text or the SI) for the production of 1 MJ of e-fuel in 2023 (with the background technosphere being ecoinvent 3.9.1) and 2050 (with the background technosphere being consistent with REMIND SSP2-RCP2.6), decomposed into electricity production, processing, and other processes. This figure shows that the dominant driver of both absolute impacts and their evolution over time is the electricity production mix, whereas the contribution of the processing step remains comparatively smaller and changes only marginally over time.

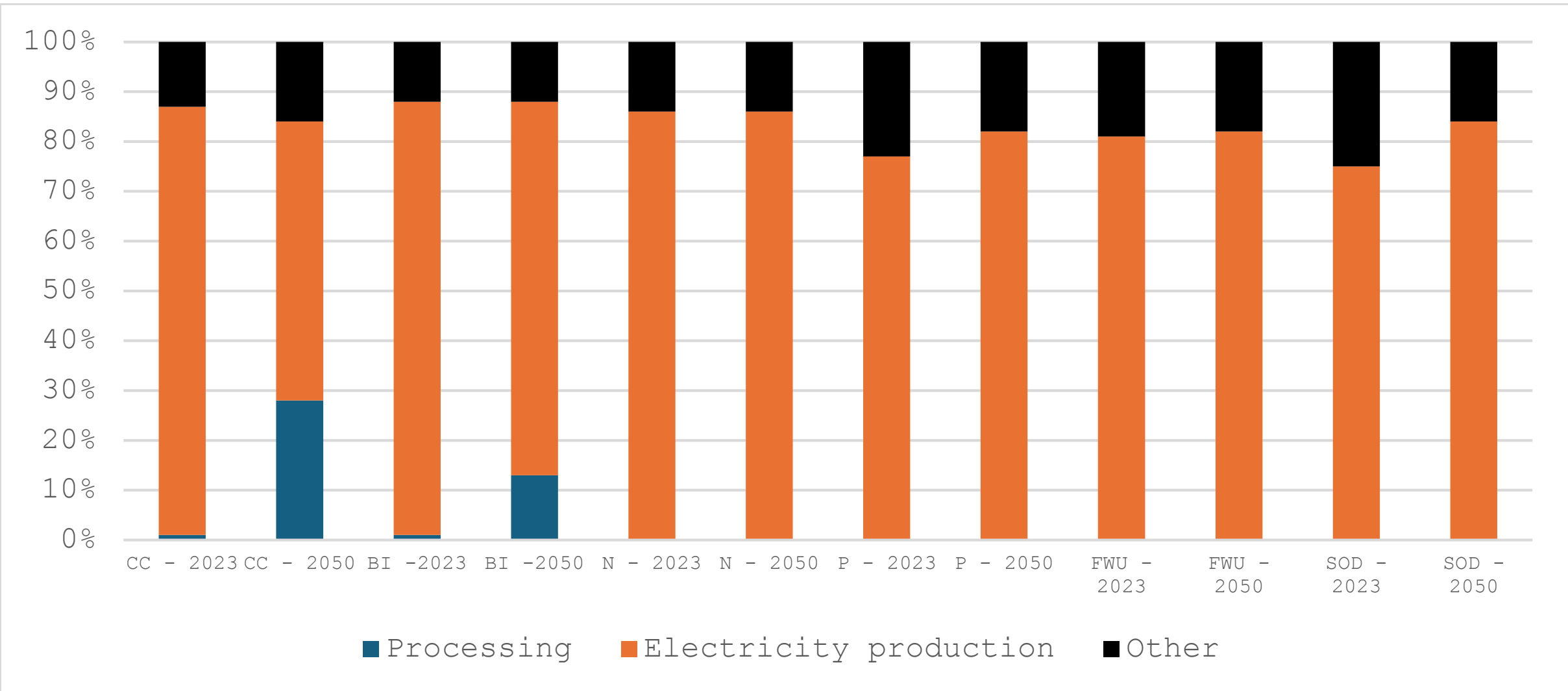

Figure 1 - Contribution analysis for the production of 1 MJ of e-fuel in 2023 and 2050. CC = Climate change, BI = Biosphere integrity, N = Nitrogen cycle, P = Phosphorus cycle, FWU = Freswhater use, and SOD = Stratospheric ozone depletion.

Importantly, in our prospective framework, the technological evolution of the background technosphere is represented dynamically through the background systems generated by *premise* consistent with IAM scenarios. Therefore, although the foreground e-fuel process structure is based on an earlier reference, the key drivers of environmental performance evolve over time in our model. Using more recent foreground inventories would mainly affect secondary process parameters and absolute impact levels, but is not expected to alter the relative ranking of scenarios nor the qualitative conclusions regarding planetary boundary transgressions.

Concerning the second question: as explained in the text, the climate-based selection is the most relevant as the roadmaps analysed were designed to mitigate climate change. We view the other PB-based selection as a way to assess the sensitivity of our results to this choice, and since all sensitivity analysis is carried out in the Supplementary Information, it seems logical that results for the PB-based selection are also reported in the SI. Moreover, moving these results to the main text would only make the paper more complex, but this was already pointed as a limitation of our paper by Reviewer #2 (Comment #2). To make this clear we have added the following sentence at the end of the "scenarios overview" section:

To assess the sensitivity of our results to this choice of production pathways, a different choice of biofuel production pathways geared at mitigating impacts in a balanced way across PBs is also considered in the Supplementary Information (Supplementary Table SI 5 and Fig. SI 8, 14 and 15).

Detailed comment #13

- Figure 3

o It seems like the legend box is cut, missing the bottom half.

Answer

We thank the reviewer for pointing this out. The legend box in Figure 3 was indeed partially cut in the previous version. This has now been corrected, and the full legend is properly displayed in the revised manuscript (Fig. 3 in the first version has become Fig. 4 in the new version).

Detailed comment #14

- Figure 4

o The icon for climate change is not the same as the ethic-based allocation, the economic based allocation and the figure legend

Answer

We thank the reviewer for the comment and have made the necessary harmonization of the icons.

Detailed comment #15

- Table SI 3: AEC is used for Alkaline Electrolysis Cell, in part two about Alternative Energy Carriers, also abbreviated as AEC.

o We suggest that AEC is reserved for the Altrenative Energy Carriers, as it is used in the manuscript as well.

Answer

Thank you, we have removed the acronym AEC in reference to Alkaline Electrolysis Cell.

Detailed comment #16

- Table SI 5: for HEFA, Capaz et al. Used cooking oil sum of rank is 216

o The right sum is 16.

Answer

Thank you, we have corrected the typo.

Detailed comment #17

- Figure SI 6: Electricity mixes come from premise dashboard.

o Premise versions have different assumptions and give different results. We suggest that you add the version.

Answer

We thank the reviewer for this comment. We have now specified the version of *premise* used to generate the electricity mixes. All results are based on *premise* version 2.2.0, which has been added in the caption of Figure SI 6 and in the methodological description.

Detailed point by point response

Reviewer #1

Comment #1

In the response, the authors state that the selected targets correspond to social groups that perform well in both environmental sustainability and social desirability. However, it remains unclear whether the 24 developing countries selected in the study genuinely satisfy these two criteria. Additionally, it is not demonstrated whether developed countries perform worse than the selected developing countries in these dimensions. Otherwise, this may give readers the misleading impression that developing countries systematically outperform developed countries in both environmental sustainability and social desirability, or that only developing countries are capable of meeting these criteria.

Answer

We thank the reviewer for asking this clarification. First, we wish to clarify that no known allocation principle of the share of safe operating space is without critics, and we are fully aware of this, as also well demonstrated in earlier work[1]. Acknowledging this, we here decided to consider two contrasted principles; by GDP and by FHN. The general idea of the ethic-based allocation (FHN) is indeed to select countries that perform well from an environmental and social perspective, but measuring this performance necessarily hinges upon the indicators considered. A specific stream of research is devoted to studying such indicators, with for instance more than 700 indicators proposed to measure social foundations[2]. For this reason, we believe that "demonstrating" or "genuinely" showing that a country satisfies these two criteria is a very difficult (if even possible) endeavor reaching far beyond the scope of the present paper. To address this methodological complexity, we therefore need to build on state-of-the-art results. Here we have chosen to use the recent methodology of Heide et al. that proposes to assess the environmental and social performance through three widely accepted indicators (human development index, carbon footprint per capita and ecological footprint per capita)[3]. This choice of indicators is not biased toward or against certain categories of countries and the resulting set of reference countries directly follows from it. To illustrate the performance of developed vs developing countries, we have added a new figure in the SI

---

[1] Ryberg, M. et al. Downscaling the planetary boundaries in absolute environmental sustainability assessments – A review. Journal of Cleaner Production (2020); see also the recent publication from the European Commission, Guidance for applying absolute environmental sustainability assessment on activities at different scales (2025).

[2] Li, J. *et al*. Revisiting social foundations and well-being indicators for sustainability: Insights from a systematic literature review. Ecological Indicators (2025)

[3] Heide, M. *et al*. Reflecting the importance of human needs fulfilment in absolute sustainability assessments: Development of a sharing principle. Journal of Industrial Ecology (2023)

(Figure SI 18) showing how countries perform across these three indicators. To introduce this figure, we have added the following sentence in the Method section of the main text:

"The resulting reference countries tend to be developing countries due to the fact that developed countries usually fail to satisfy both environmental criteria: the performance of developed vs developing countries is analyzed in more details in Fig. SI 18 (SI, Section 6)" (Line [667])

This figure corroborates earlier findings showing that developed countries tend to have good social performance at the expense of bad environmental performance, while the situation is reversed for developing countries[4]. Such behavior may explain the potential misleading impression mentioned by the reviewer and we hope that the new Figure SI 18 will help avoiding it.

Comment #2

The authors acknowledge that the FHN allocation method "tends to maintain the status quo and may not be favorable to the development of future technologies," which is an important and reasonable point. However, the response does not fully address the reviewer's core concern. First, the authors only acknowledge this limitation qualitatively, without assessing the extent to which this methodological issue may affect the study's conclusions. For instance, under scenarios where aviation transitions toward biofuels or e-fuels, its impacts on planetary boundaries such as nitrogen cycles and land use are likely to increase significantly. In this context, the FHN method - by allocating extremely small shares based on the current structure (e.g., 0.002% for phosphorus) - may systematically amplify the extent of "boundary transgression." The authors do not evaluate whether such bias could alter the main conclusions. Second, it would be beneficial to consider introducing alternative allocation methods or conducting sensitivity analyses to improve the robustness of the results.

Answer

We thank the reviewer for this important comment. We fully concur with the shortcoming of the FHN approach pointed out by the reviewer, which we already acknowledge in the paper. Concerning the first point on whether this bias could alter the main conclusions: actually, our main conclusion is that the two core planetary boundaries (climate change and biosphere integrity) are systematically transgressed, irrespectively of the allocation principle considered. This conclusion is clearly stated in the following passages of the article:

[4] O'Neill, D. *et al*. A good life for all within planetary boundaries. Nature Sustainability (2018)

- in the abstract: "we find persistent exceedance of the climate change and biosphere integrity core boundaries";
- in the introduction: "Our findings demonstrate that none of the currently proposed roadmaps for aviation climate mitigation remains within planetary boundaries, with systematic transgression of both climate change and biosphere integrity";
- in the section *Current aviation roadmaps are not absolutely sustainable*, the conclusion that aviation roadmaps are not absolutely sustainable is based on the transgression of the two core planetary boundaries: "Given the prime importance of the climate change and biosphere integrity, these being core PBs, we thus conclude that current roadmaps are not absolutely sustainable.";
- in the discussion, only the transgression of the two core planetary boundaries is discussed.

In contrast, nowhere in the paper do we conclude about absolute unsustainability based on the consideration of the phosphorus or nitrogen PBs which, as rightly mentioned by the reviewer, is quite fragile. Therefore, we consider that our main conclusion is robust with respect to the bias introduced by FHN. In order to accommodate the reviewer's comment, we have added the following sentence in the section *Current aviation roadmaps are not absolutely sustainable* to put more emphasis on the fact that the transgression of the phosphorus and nitrogen PBs depends on the allocation principle:

"In particular, we conclude that the transgression or respect of these two planetary boundaries depends on the allocation principle considered." (Line [201])

Concerning the second point raised by the reviewer about considering alternative allocation methods or conducting sensitivity analysis. In the paper, the allocated share is the mean across reference countries of the relative impact in each country: to assess the robustness of our results to this variation, we have added a new figure in the SI (Figure SI 19) that shows more information by displaying boxplots across reference countries, thereby conveying orders of magnitude about the dispersion around the mean. This figure demonstrates that our results are robust to this variation. Finally, concerning the consideration of alternative allocation methods, we note that considering two allocation methods based on very different premises already advances the state-of-the-art compared to papers published in high-impact journals. For instance, a paper published last month in *Nature Communications* assessing the absolute sustainability of hydrogen production only considers one allocation principle (which, moreover, is quite crude as it assigns the same share to every PB)[5]. Other examples of papers published in high-impact journals and considering only one allocation principle include Algunaibet et al. on power

[5] Lejeune, M. *et al*. Pathways to global hydrogen production within planetary boundaries. Nature Communications (2026)

systems[6], Bachmann et al. on plastic production[7] and Springmann et al. on food production[8]. Therefore, we believe that our results match (or even go beyond) the best current scientific practices.

We hope that the clarification about the scope of our main conclusions and the addition of the new figure assessing the robustness of our results to variation of the FHN shares will satisfactorily address the reviewer's comment.

Comment #3

The authors emphasize in their response that the study focuses on the allocation of the Safe Operating Space (SOS), rather than on the attribution of aviation emissions. However, this clarification does not fully address the reviewer's main concern regarding the appropriateness of the chosen allocation metrics (GDP and RPK). Regardless of the object of allocation, the fundamental issue remains how to define the environmental share that should be attributed to the aviation sector. The use of GDP and RPK as proxies for "social utility" and "transport utility," respectively, is debatable. GDP does not reflect the necessity or equity of different economic activities, while RPK merely measures the scale of transport activity, ignoring key aspects such as travel purpose, substitutability, and distributional inequality. In a sector like aviation, which is highly unequal and partially characterized by luxury demand, these indicators may systematically overestimate its justified environmental share.

Answer

We could not agree more with the reviewer's comment, but we are back our answer to the Comment #1: any metric has its pros and cons and is debatable. Most papers published to date only consider one metric, and most of the time this is an economic metric such as GDP (see our answer to the Comment #2). Therefore, we believe that our results at least match the best current scientific practices. Actually, defining actionable allocation principles taking the key aspects mentioned by the reviewer (travel purpose, inequality, etc) represents important research gaps on which many members of the AESA community are currently working. This being said, despite their shortcomings, considering GDP and RPK has significant merits:

1. GDP and FHN are in line with the AESA guidelines of the European Commission[9];

---

[6] Algunaibet, I. *et al*. Powering sustainable development within planetary boundaries. Energy & Environmental Science (2019)
[7] Bachmann, M. *et al*. Towards circular plastics within planetary boundaries. Nature Sustainability (2023)
[8] Springmann, M. *et al*. Options for keeping the food system within environmental limits. Nature (2018)
[9] Bjørn, A. *et al.* Guidance for applying absolute environmental sustainability assessment on activities at different scales (BETA version). *JRC Publications Repository*

2. GDP (or a related economic indicator such as gross added value) constitutes an important benchmark as it is considered in many previous AESA studies;
3. FHN is the only allocation principle that we know of which is based on ethical consideration and can be applied to the aviation sector;
4. Most previous AESA studies only consider one allocation principle (see answer to Comment #2);
5. Beyond GDP (or related economic indicator) and RPK, we are not aware of other metrics that could be used as proxy for "utility". In particular, to our knowledge, no actionable metric captures key aspects such as those mentioned by the reviewer.

Thanks to the reviewer comment, we realized that these shortcomings and merits were not sufficiently discussed in our paper, although they constitute important research questions for future research. Therefore, we have added the following paragraph in the discussion, which partly uses the reviewer's comment as such:

"We note finally that, as in any AESA, our results depend on the allocation methods considered. By considering two methods rooted in different principles, our results cover a large span of possible allocations which make them robust, but there are still shortcomings. First, both approaches suffer from a status quo problem, whereby the current situation is used for allocation in the future. Especially for the ethic-based approach, this can be adverse to the development of new technologies. Moreover, the two approaches ultimately rely on metrics (gross domestic product [GDP] for the economic-based approach and revenue passenger kilometer [RPK] for climate change allocation in the ethic based approach) meant to be proxies of a notion of "utility" for the aviation sector, but these metrics are debatable: GDP does not reflect the necessity or equity of different economic activities, while RPK merely measures the scale of transport activity, ignoring key aspects such as travel purpose, substitutability, and distributional inequality. In a sector like aviation, which is highly unequal and partially characterized by luxury demand, these indicators may systematically overestimate its justified environmental share. Therefore, designing allocation schemes accounting for these key aspects also represents an important research direction." (Line [519])

Comment #4

In response to Comment 4, the authors only discuss the impact of non-CO_2 effects qualitatively, without providing any quantitative estimates or range analysis. Even if substantial uncertainties exist, providing a plausible range based on existing literature or conducting a simple sensitivity analysis would be essential for assessing the robustness of the results. The current lack of quantitative information makes it difficult for readers to evaluate the actual impact of this omission on the study's conclusions, particularly regarding the magnitude of boundary transgression and related policy implications.

Answer

We thank the reviewer for this comment. We have added in the SI (new Fig. SI 17) a discussion about potential impacts of contrail effects, which are the main driver of non-CO_2 effects. Since we are not aware of any result linking aviation non-CO_2 impacts to planetary boundaries other than climate change, we only account for contrail impacts on climate change and biosphere integrity, the latter being partly driven by climate impacts.

To account for climate impacts of contrail formation, we use the methodology of Lee et al.[10]: first, we assess the amount of kilometers flown by aircraft in 2050 (which, in our work, amounts to 174×10^9 km), and then multiply this amount by the ERF coefficient on a kilometer basis, whose central estimate is $9.36.10^{-10}$ $mW.m^{-2}.km^{-1}$. As in Lee et al., we then assess the sensitivity of this central estimate by considering a range of +/-70%: Results are displayed in the new Fig. SI 17, and show that contrail effects may increase impacts on climate change and biosphere integrity by significant amounts, from +30% to +900% with the above methodology, depending on the scenario and the planetary boundary considered.

It is important to keep in mind that applying the methodology of Lee et al. to prospective scenarios faces several methodological limitations. In particular, the following effects are known to potentially affect the impacts of non-CO_2 effects:

1. Climate impacts of cirrus depend not only on the volume of air traffic, but also on how it is geographically and temporally distributed[11];
2. Alternative energy carriers may have different impacts than fossil kerosene on non-CO_2 effects, especially on contrail formation and induced radiative forcing, but there are still significant uncertainties[12].

These potential effects are not assessed in the above estimates, which thus warrant further investigation.

Comment #5

In the main text (Line 60), the authors state that the annual improvement rate of aviation energy efficiency is 1.5%·yr^{-1}, indicating that this value corresponds to the median of ICAO scenarios. However, the caption of Figure 1 uses 1.7%·yr^{-1} as the assumed

---

[10] Lee, D. S. *et al*. The contribution of global aviation to anthropogenic climate forcing for 2000 to 2018. *Atmos. Environ*. **244**, 117834 (2021). To our knowledge, this source is the most up-to-date reference for the assessment of aviation non-CO_2 impacts. For instance, it constitutes the basis of the recent report from the Aviation non-CO_2 expert network (ACEN) of the EASA (European Union Aviation Safety Agency) published in March 2026 (*Gaps and uncertainties in aviation non-CO2 effects on climate,* Task 4 report, March 2026).

[11] Teoh, R. *et al*. Global aviation contrail climate effects from 2019 to 2021, Atmospheric Chemistry and Physic (2024)

[12] Lee, D. *et al*. Uncertainties in mitigating aviation non-CO_2 emissions for climate and air quality using hydrocarbon fuels. Environmental Science: Atmospheres (2023)

parameter for the same scenario. Since both values are used to describe the same demand pathway for 2023–2050, the authors are encouraged to clarify which value is actually implemented in the model. If different sources or scenario settings are involved, these should be explicitly explained in the main text and presented consistently.

Answer

We thank the reviewer for pointing out this inconsistency. The value of 1.7%·yr^{-1} reported in the caption of Figure 1 was a typographical error. The correct value implemented in the model is 1.5%·yr^{-1}, as stated in the main text.

We have corrected this inconsistency in the revised version of the Figure 1 caption, and we confirm that all results presented in the manuscript are based on the 1.5%·yr^{-1} efficiency improvement assumption.

Comment #6

In the Supplementary Information, the "Figures" section of the Summary contains a large number of Figure SI items. It is recommended to group these figures into thematic categories (e.g., AESA, resource requirements, etc.) to improve readability. This would also facilitate more precise referencing in the main text.

Answer

We thank the reviewer for this helpful suggestion. We have revised the Supplementary Information accordingly by organizing the figures into thematic sections, and updated the corresponding references in the main text to reflect this structure.

Comment #7

In Figure SI 7 of the Supplementary Information, the legend includes both green and red color ranges, whereas the data displayed in the figure appear to fall entirely within the green range, with no values in the red region. This inconsistency between the legend and the actual data distribution may increase the cognitive burden for readers and potentially lead to confusion regarding the meaning of the unused color range. It is therefore suggested to adjust the color legend accordingly, for example by adopting a single-hue gradient or redefining the color scale based on the actual data range. This would allow the legend to more accurately reflect the data distribution and improve both the readability of the figure and the clarity of differences.

Answer

We thank the reviewer for this helpful comment. We have updated the figure to adopt a single-hue gradient.

Reviewers #3, 4 and 5

General comment

Dear Authors,
Thank you for a substantially revised manuscript. The methodological novelty is better articulated in the revised version, and the results are clearly presented. The study will be valuable for both policymakers and researchers interested in the AESA framework. The comments were well answered. We have no major concerns; however, we suggest the following minor revisions to further strengthen the manuscript.

Answer

We thank the reviewer for this positive and encouraging feedback on the revised manuscript. In response to the remaining minor suggestions, we have made additional revisions to further improve the clarity and robustness of the manuscript, as detailed below.

Comment #1

Scenarios F1-F3 and S1-S3 are referenced often but only explained through the references and citations. A table or a paragraph linking the scenarios to a quick description would improve the comprehension, understanding that S1 is the baseline and S3 aggressive sustainable aviation fuel deployment and how they can be linked to the Roadmaps.

Answer

We thank the reviewer for this helpful suggestion. Concerning the link between Roadmaps and scenarios, we believe that it is already very explicit in the current version of the manuscript, and we did not find ways to accommodate the reviewer's comment without adding undesirable redundancy in the text. More precisely, this link is made explicit in the Table 3: there the link between Roadmaps and the ICAO F1-F3 scenarios is very clear, while the link with Waypoint scenarios is maybe less apparent in the "Comments" column. But we also mention in the main text (Line [105]) that "Roadmap-70, Roadmap-30-Bio and Roadmap-0 closely follow the three Mid LTAG scenarios F1, F2 and F3 of ICAO[12], respectively, while Roadmap-30-Efuel considers the evolution of the energy mix of the ReFuelEU Aviation initiative. The Waypoint 2050 scenarios S1, S2 and S3[11] assume 10% of residual fossil kerosene in 2050 and thus lie in-between Roadmap-30 and Roadmap-0, and their shares of biofuels and efuels also vary in ranges covered by the Roadmap scenarios (SI Section 1, Fig. SI1). »

This being said, we concur with the reviewer that these scenarios could be better described. Thus, in order to address the reviewer, we have added the following sentence in the section *Scenarios modeling current roadmaps*:

"ICAO's LTAG scenarios are labelled F1 to F3: F1 emphasizes low-cost greenhouse gas reduction, F2 prioritizes effective greenhouse gas reduction and F3 maximizes CO_2 reduction. The ATAG Waypoint scenarios are labelled S1 to S3: S1 pushes technology and operations, S2 considers aggressive sustainable fuel deployment and S3 aspirational and aggressive technology perspective." (Line [50])

We hope that this clarification and addition will satisfactorily address the reviewer's comment.

Comment #2

Line [59]: "12 trillion MJ".

In Figure 1, the y axis is graduated in steps of 0.5e13 MJ, when it could just be in 5e12 MJ, or even better, use "12 PJ" in the text and 5 PJ for the y-axis ticks.

Answer

We thank the reviewer for this helpful suggestion. We agree that expressing energy values in MJ reduces readability at this scale. We have therefore revised the manuscript and Figure 1 to express energy in exajoules (EJ), which are more appropriate for values of this magnitude.

The value in the main text is now reported in EJ, and the y-axis in Figure 1 has been adjusted accordingly to improve readability and consistency

---

Comment #3

Line [60-61]: "These assumptions correspond to the central values of the ICAO scenarios and are consistent with the Waypoint 2050 roadmaps". 3.7%.yr-1 of increase in traffic is indeed a central value of the ICAO and could be considered as consistent with the Waypoint roadmaps, however the 1.5%.yr-1 improvements in efficiency is not a central value of the ICAO but is consistent with the Waypoint roadmaps. We suggest that the sentence is reworked to better explain the choices of those values and how they fit into the scenarios.

Answer

We thank the reviewer for this important clarification. We agree that the original sentence could be misleading. While the traffic growth rate (3.7%·yr^{-1}) corresponds to the central value of the ICAO scenarios, the efficiency improvement rate (1.5%·yr^{-1}) does not fall within the ICAO central range (0.7-1.3%·yr^{-1}), but is consistent with the values reported in the Waypoint 2050 scenarios (1.4-1.9%·yr^{-1}).

We have revised the sentence accordingly to clarify the respective origins of these assumptions and improve consistency:

"The traffic growth rate corresponds to the central value of the ICAO scenarios, while the efficiency improvements rate lies within the range reported in the Waypoint 2050 scenarios. Together, these assumptions are consistent with industrial and academic forecasts." (Line [62])

Comment #4

Line [74] Figure 1 caption: "efficiency improvements (1.7 %.yr-1)". We assume that the actual value is the one cited in the text (1.5 %.yr-1) but it should be the same or explained why the values are different.

Answer

We thank the reviewer for pointing out this inconsistency. As explained in our response to Reviewer #1, Comment #5, the value of 1.7%·yr^{-1} reported in the caption of Figure 1 was a typographical error. The correct value implemented in the model is 1.5%·yr^{-1}, as stated in the main text.

This has now been corrected in the revised version of Figure 1 caption.

Comment #5

Line [140] Table 3: For the monofuel scenarios, the use of "=" for the roadmap on which the scenarios are based is not clear. We suggest that it be replaced by "N/A" if it's non applicable, or "RefuelEU" if it's the same scenario as the one above that is used.

Answer

We thank the reviewer for this suggestion. We agree that the use of "=" in Table 3 for the mono-type scenarios was unclear. These scenarios are not directly based on existing institutional or industrial roadmaps, but are instead illustrative scenarios designed to explore the influence of specific energy pathways.

We have therefore replaced "=" with "N/A" in Table 3 to reflect that no corresponding roadmap applies to these scenarios.

Comment #6

Line [140] Table 3: For Efuel-RES comments "100% efuef from renewable energy" should be corrected as "efuel".

Answer

We thank the reviewer for pointing out this typographical error. The term “efuef” in Table 3 has been corrected to “efuel” in the revised manuscript.

Comment #7

Figure 3 and 4: While distinguishable with a screen, the 3 alternative energy carriers in green are very hard to tell apart on paper. We recommend that the authors check with black and white filters the readability of the graph.

Answer

We thank the reviewer for this helpful suggestion. We agree that the distinction between alternative energy carriers was not sufficiently clear in print, particularly under grayscale conditions.

We have therefore revised Figures 2, 3 and 4, as well as the figures in the Supplementary Information, by adopting a colorblind-friendly palette with improved contrast between categories.

In addition, we have verified the readability of all figures using grayscale and color-vision deficiency filters, ensuring that all categories remain clearly distinguishable when printed or viewed by readers with color vision impairments.

Finally, we have added black outlines to the graphical elements of Figure 3 to further enhance contrast and readability in print.

Comment #8

Figure 3: The comparison to baseline, using only one decimal makes it so that 3a) is showing x0.8 for significantly different results for Roadmap-30-bio and Roadmap-0. We recommend that the comparison be expanded to two decimals, if possible, to better represent the comparison in between scenarios as well.

Answer

We thank the reviewer for this helpful comment. This remark allowed us to identify and correct numerical inaccuracies in Figure 3, as well as an inconsistency in panel (a), where arrows did not point to the net result.

Following the reviewer’s suggestion, we produced an updated version of the figure with values reported to two decimal places (see below), which indeed provides a more precise comparison between scenarios. However, we found that this level of numerical detail substantially increases visual clutter and reduces the overall readability of the figure in

the main manuscript. Moreover, once the numerical errors are corrected and the arrows point to the net value (represented by diamonds), it seems to us that the problem pointed out by the reviewer between Roadmap-30-bio and Roadmap-0 is not so salient anymore: 0.74 for Roadmap-30-bio is rounded to 0.7 and 0.77 for Roadmap-0 to 0.8, but since the height difference between the net values (the diamonds) is more pronounced than between the gross values (the height of the bars), we believe that this is not problematic anymore. Note also that the absolute and precise values are provided in the data spreadsheets. For these reasons we chose to retain one decimal place in the revised manuscript. In our view, this provides the best balance between precision and readability. However, if preferred by the editor and reviewers, we would be happy to replace it with the two-decimal version below.

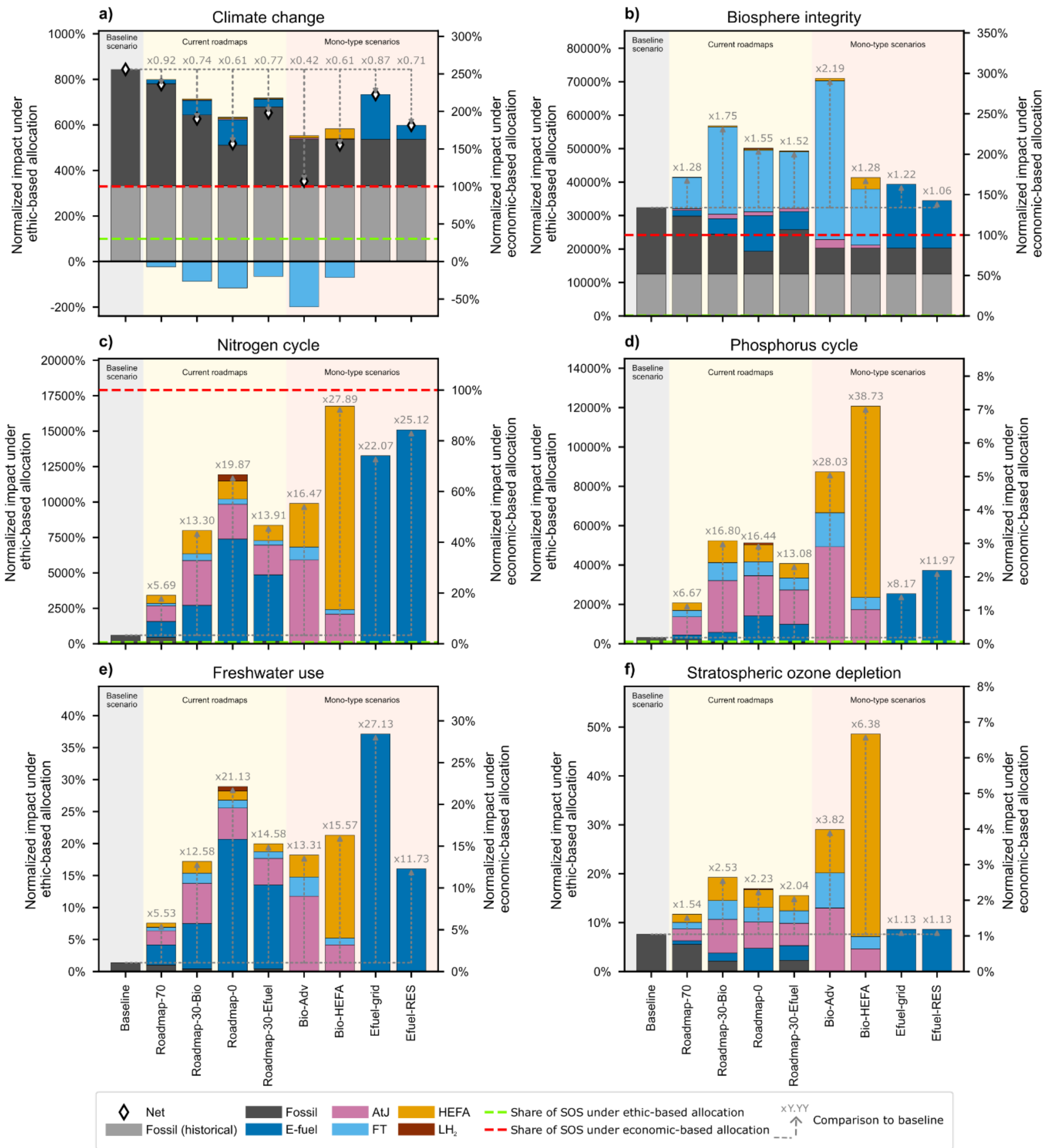

Comment #9

Line [664]: "As explained in the original publication, there thresholds" Does "the original publication" refers to reference 22? We recommend adding it to clear any doubt the reader might have. "There" should probably be "those".

Answer

We thank the reviewer for this clarification. We confirm that "the original publication" refers to reference 22, and we have made this explicit in the revised manuscript to avoid any ambiguity. We have also corrected the typographical error by replacing "there thresholds" with "those thresholds".
